# Comprehensive characterization of fetal and mature retinal cell identity to assess the fidelity of retinal organoids

**DOI:** 10.1016/j.stemcr.2022.12.002

**Published:** 2023-01-10

**Authors:** Hani Jieun Kim, Michelle O’Hara-Wright, Daniel Kim, To Ha Loi, Benjamin Y. Lim, Robyn V. Jamieson, Anai Gonzalez-Cordero, Pengyi Yang

**Affiliations:** 1 Computational Systems Biology Group, Children’s Medical Research Institute, The University of Sydney, Westmead, NSW 2145, Australia; 2 School of Mathematics and Statistics, The University of Sydney, Sydney, NSW 2006, Australia; 3 School of Medical Sciences, Faculty of Medicine and Health, The University of Sydney, Camperdown, NSW 2006, Australia; 4 Stem Cell Medicine Group, Children’s Medical Research Institute, The University of Sydney, Westmead, NSW 2145, Australia; 5 Specialty of Genomic Medicine, Faculty of Medicine and Health, University of Sydney, Westmead, NSW 2145, Australia; 6 Eye Genetics Research Unit, Children’s Medical Research Institute, Sydney Children’s Hospitals Network, Save Sight Institute, The University of Sydney, Westmead, NSW 2145, Australia

**Keywords:** cell identity, retinal organoids, pluripotent stem cells, retina, eye, maturation, human eye, fidelity

## Abstract

Characterizing cell identity in complex tissues such as the human retina is essential for studying its development and disease. While retinal organoids derived from pluripotent stem cells have been widely used to model development and disease of the human retina, there is a lack of studies that have systematically evaluated the molecular and cellular fidelity of the organoids derived from various culture protocols in recapitulating their *in vivo* counterpart. To this end, we performed an extensive meta-atlas characterization of cellular identities of the human eye, covering a wide range of developmental stages. The resulting map uncovered previously unknown biomarkers of major retinal cell types and those associated with cell-type-specific maturation. Using our retinal-cell-identity map from the fetal and adult tissues, we systematically assessed the fidelity of the retinal organoids in mimicking the human eye, enabling us to comprehensively benchmark the current protocols for retinal organoid generation.

## Highlights

• Extensive meta-atlas characterization of cellular identities of the human eye
• Uncovered previously unknown biomarkers of major retinal cell types
• Discovered genes associated with cell-type-specific maturation of the human eye
• Systematically assessed the fidelity of the retinal organoids in mimicking the human eye

## Graphical abstract

**Figure:**
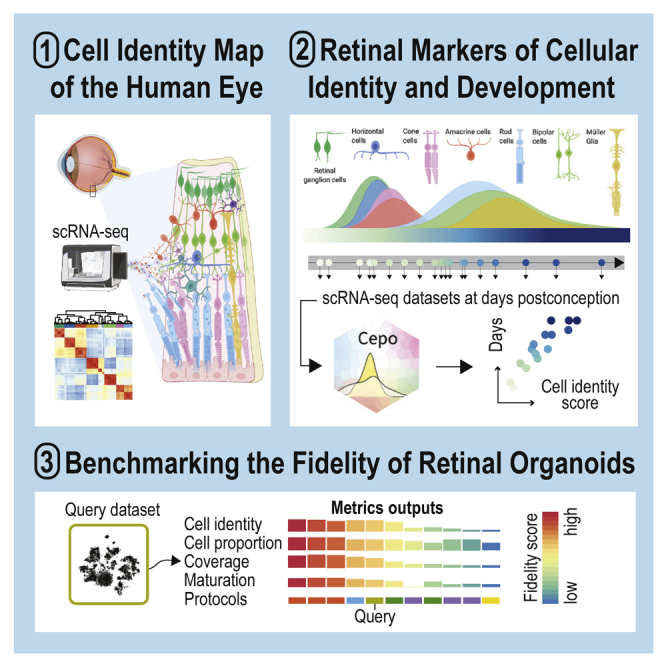

## Teaser

Human retinal organoids derived from pluripotent stem cells (PSCs) offer unprecedented opportunities to investigate early retinal development and diseases. Systematic assessment of the retinal organoids to recapitulate the human eye is critical for their successful application. Kim et al. performed an extensive meta-atlas characterization of cellular identities of the human eye to generate a retinal cell-identity reference map. They subsequently developed a computational framework and novel metrics for assessing the fidelity of retinal organoids based on this retinal cell-identity reference map.

## Introduction

The human retina is a complex tissue and comprises various cell types that function together to convert light into biological signals. To understand the development and diseases of the human eye requires the characterization of molecular and cellular programs that define the identity of cells in the human retina. While access to the human retinal tissues is limited, human retinal organoids derived from pluripotent stem cells (PSCs) offer unprecedented opportunities to investigate early retinal development and therapeutic applications such as cell transplantation (Chahine Karam et al., 2022; Ribeiro et al., 2021; West et al., 2022). Early studies have used human embryonic stem cell (hESC)-derived embryoid bodies followed by two-dimensional (2D) culturing to generate retinal precursors, which were then isolated for directed and undirected differentiation into ganglion and amacrine cells and, to a lesser extent, photoreceptor precursor cells (Lamba et al., 2006). While the efficient production of the retinal progenitors under 2D conditions enabled useful initial applications in cell therapy studies (Lamba et al., 2006, 2010; Meyer et al., 2009; Osakada et al., 2008), these systems lack the capacity to recapitulate the three-dimensional (3D) features of the native retinal cells *in vivo*. This has led the field to develop advanced 3D *in vitro* structures that can recapitulate the physiological, morphological, and spatiotemporal patterns of the developing retina (Eiraku et al., 2011; Meyer et al., 2011; Nakano et al., 2012; Zhong et al., 2014).

The advances in the retinal organoid field have led to the development of state-of-the-art protocols that allow efficient and rapid formation of retinal organoids comprising all the retinal cell types: ganglion, amacrine, bipolar, horizontal, Müller glial, and photoreceptor (Afanasyeva et al., 2021). These organoids have been shown to generate mature features such as ribbon synapses (Artero Castro et al., 2019) and outer segments with physiological response to light stimuli (Wahlin et al., 2017; Zhong et al., 2014), showing remarkable functional similarity to the eye (Gonzalez-Cordero et al., 2017). A mixture of 2D and 3D protocols that do not require the addition of small molecules allows the generation of mature and light-sensitive photoreceptors with rudimentary outer segments (Zhong et al., 2014). Other protocols that involve a stepwise 2D-to-3D culture enable the formation of the embryoid body to be bypassed (Reichman et al., 2014). Other protocols have incorporated differentiation factors such as serum, retinoic acid (RA), taurine, and supplements N2 and B27 (Gonzalez-Cordero et al., 2017) and antioxidants and lipids (West et al., 2022) that have significantly improved the generation of photoreceptor outer segments. Most of these protocols share common medium components, such as BMP4 and IGF-1, but differ in their timing in the switch from 2D to 3D culture and/or the addition of certain molecules.

An increasing number of studies have begun profiling the human organoids derived from these protocols at single-cell resolution to investigate retinal development and disease (O’Hara-Wright and Gonzalez-Cordero, 2020). These studies have provided an unprecedented opportunity to investigate the heterogeneity of the retinal cell types and uncovered several new insights into retinal development, such as the discovery of a potentially novel regulator of cone fate (Kallman et al., 2020), a population of post-mitotic transitional cells (Sridhar et al., 2020), and the convergence of retinal organoid transcriptomes toward peripheral retinal cell types (Cowan et al., 2020). While these advancements have enhanced our understanding of retinal biology, in particular our understanding of the differences between retinal cell types between subdomains of human tissue (Voigt et al., 2021; Yan et al., 2020), the growing single-cell resource of the human retina and retinal organoids has yet been probed to systematically evaluate the state-of-the-art protocols for their capacity to produce organoids faithful to their *in vivo* counterpart.

Here, we performed an extensive curation of single-cell RNA-sequencing (scRNA-seq) datasets from human retinal tissue and organoids derived from a variety of differentiation protocols, generated a comprehensive map of retinal cellular identities of the mature and fetal eye, and benchmarked the fidelity of the human retinal organoid models in faithfully recapitulating the human eye. The extensive meta-atlas characterization of the retinal cellular identities enabled the discovery of an array of previously unknown marker genes of retinal cell types and those associated with cell-type-specific retinogenesis. Moreover, these cellular identities resolved by age were used to systematically benchmark the current protocols for their capacity to generate cell types that closely emulate their *in vivo* counterparts in terms of cell identity, cell-type proportion, and coverage. Finally, we developed a user-friendly application called Eikon (https://shiny.maths.usyd.edu.au/Eikon/) that helps users assess the fidelity of their retinal organoids.

## Results

### Generating a cell-identity map of the human retina

**Figure 1:**
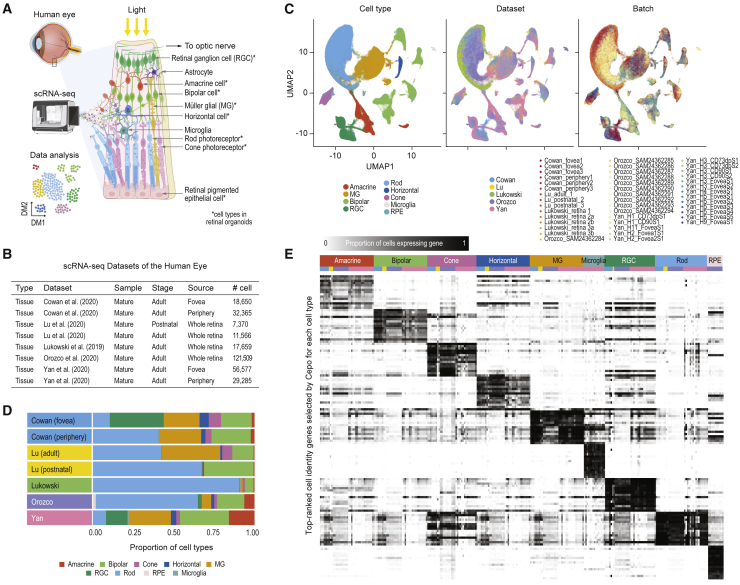
Curation of scRNA-seq datasets generated from the mature retinal tissue (A) Schematic of single-cell transcriptomic profiling by scRNA-seq and the major cell types of the human retina. (B) Summary of scRNA-seq datasets collected from the mature retinal tissues. (C) UMAP representation of the transcriptomes of single cells. Cells are colored by their type (left), dataset of origin (middle), and batch in each dataset (right). Abbreviations: MG, Muller glial; RGC, retinal ganglion cell; RPE, retinal pigment epithelium. (D) Proportions of cell types (color coded) and total number of cells in each batch and dataset. (E) Expression patterns of known retinal cell-type marker genes across datasets and batches. As in (C), the color annotation denotes dataset and batch information.

Single-cell transcriptome profiling was applied to resolve the cellular identities in the retinal tissue (Figure 1A). To create a cell-identity map of the human retina, we began by compiling a collection of scRNA-seq datasets generated from the human retinal tissue in “mature” samples, including those from the postnatal stage and the adult retina (Figures 1B and S1A) (Cowan et al., 2020; Lu et al., 2020; Lukowski et al., 2019; Orozco et al., 2020; Yan et al., 2020).

We visualized the integrated datasets by cell type, dataset, and batch within each dataset using uniform manifold approximation projection (UMAP) (Figure 1C) and analyzed the number of cells in each cell type and their proportions in each dataset and batch (Figures 1D, S1B, and S1C). We observed that, while most of the major cell types are identified in the datasets and the proportions of cell types are largely consistent across batches within each dataset, the proportions of some cell types showed large variability across datasets (Figure S1C). Evaluating known retinal cell-type gene markers showed that their expression is highly cell-type specific (Figure 1E). To ensure the derivation of a high-quality reference, we performed re-annotation of all the cells in our retinal tissue meta-atlas using scReClassify, which is a semi-supervised learning method for assessing cell-type annotation accuracy in the original classification and for deriving classification accuracy probabilities (Kim et al., 2019). Keeping only the cells that had been correctly assigned their original annotations, we found that most cells in the reference were annotated with very high confidence (Figure S1D). To generate the gold standard reference for use in our downstream analyses, we included only the cells annotated with “very high” confidence (probabilities greater than 0.9, where 1 denotes the highest level of confidence). We show that, in most datasets (except Lukowski et al., 2019), greater than 90% of cells were assigned with high to very high confidence, enabling us to retain the majority of the cells for downstream analyses. Together, this large resource of scRNA-seq datasets profiling the human eye forms the basis for the characterization of the human retina cell types and for assessing the retinal cell identity and the fidelity of human retinal organoids in mimicking the *in vivo* identities.

### Deriving robust cell-identity scores of genes for retina cell types

**Figure 2:**
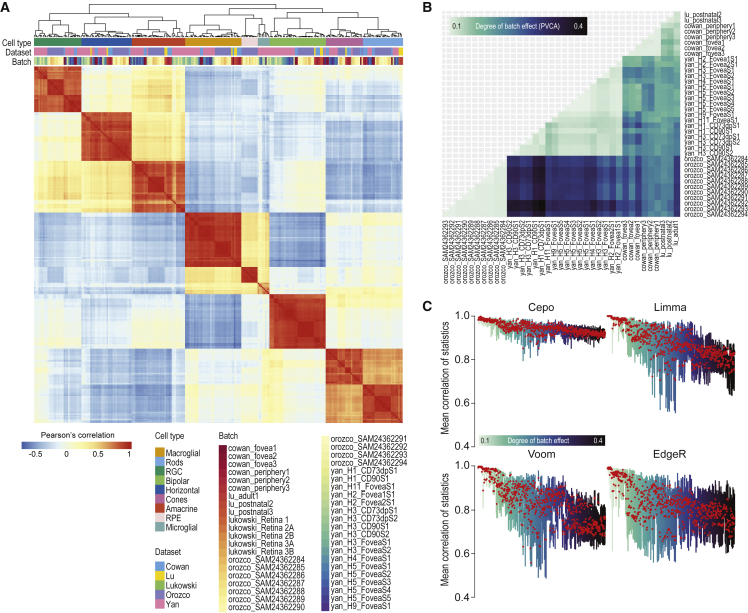
Comparative analysis of cell-type-specific gene statistics across datasets and batches (A) Correlation heatmap of cell-identity gene statistics generated from Cepo (Kim et al., 2021) for each cell type across datasets and batches. The heatmap is hierarchically clustered by the similarity of correlation profiles. (B) Pairwise assessment of batch effect using principal variance component analysis (PVCA). The proportion of variance contributed by batch in each pair of datasets is visualized. A darker color denotes stronger batch effect. (C) Boxplots of mean correlation of gene statistics from all retinal cell types for each pair of datasets illustrated in (B) using Cepo, Limma, Voom, and EdgeR statistics.

To resolve genes that underlie retinal cellular identity, we computed a cell-type-specific cell-identity score for each gene by dataset and batch using Cepo, a computational method for detecting cell-identity genes (Kim et al., 2021). The clustering of samples from across datasets and batches using Pearson’s correlation of Cepo-derived gene statistics shows strong grouping by cell type irrespective of the origin of dataset and batch (Figure 2A).

To systematically quantify the influence of the total number of batches and the batch source on Cepo-derived cell-identity gene statistics, we conducted three assessments. First, we evaluated the stability of the Cepo statistics by randomly subsampling from 50% to 90% of all data batches for inclusion in the generation of the averaged Cepo scores. We then calculated Pearson’s correlation between these scores against those generated from the entire batches. Our findings show that even with subsampling of up to 50% of the batches, the Cepo statistics remain highly reproducible and stable, showing only 0.01 loss in correlation across the cell types (Figure S2A). Next, we applied different clustering methods on the samples and compared their concordance with respect to three sources of variation: cell type, dataset, or batch (see supplemental information). We found that the Cepo-derived cell-identity gene statistics enabled accurate clustering of samples by their cell type label, whereas both dataset and batch source had minimal influence on the clustering (Figure S2B). These findings were consistent across a varying number of genes (Figure S2B) and demonstrate the high reproducibility of the cell-type identity statistics calculated using Cepo for genes across our retinal resource.

Finally, we performed principal variance component analysis (Li et al., 2009) on all pairs of batches across datasets to quantify the degree of variance contributed by batch source (Figure 2B). Computing the degree of batch effect present in all pairs of batches generates a set of batch pairs ranging from those that exhibit low batch effect to those that exhibit high batch effect. As expected, batch pairs with both originating from the same dataset demonstrate lower batch effect, while those originating from different datasets demonstrate higher batch effect. We then computed the concordance of Cepo-derived gene statistics between the dataset pairs for each of the cell types and then evaluated these statistics against the degree of batch effect present in the data (Figures 2C and S2C). We found that the concordance in Cepo-derived gene statistics between the same cell types was largely retained across increasing batch effects (Figure S2C, first column). When comparing the robustness of other measures of cell-type-specific gene statistics (Limma, Voom, and EdgeR) against batch effect, we found that there was a gradual loss in concordance with increasing presence of batch effect across most cell types in these methods, with the most pronounced decrease in the statistics for the amacrine and bipolar cells (Figures 2 and S2C). Collectively, these results strongly support the high reproducibility of the Cepo-derived cell-identity gene statistics across the retina scRNA-seq datasets, highlighting their robustness against batch effect.

### The retinal-cell-identity map uncovers novel cell-identity genes

**Figure 3:**
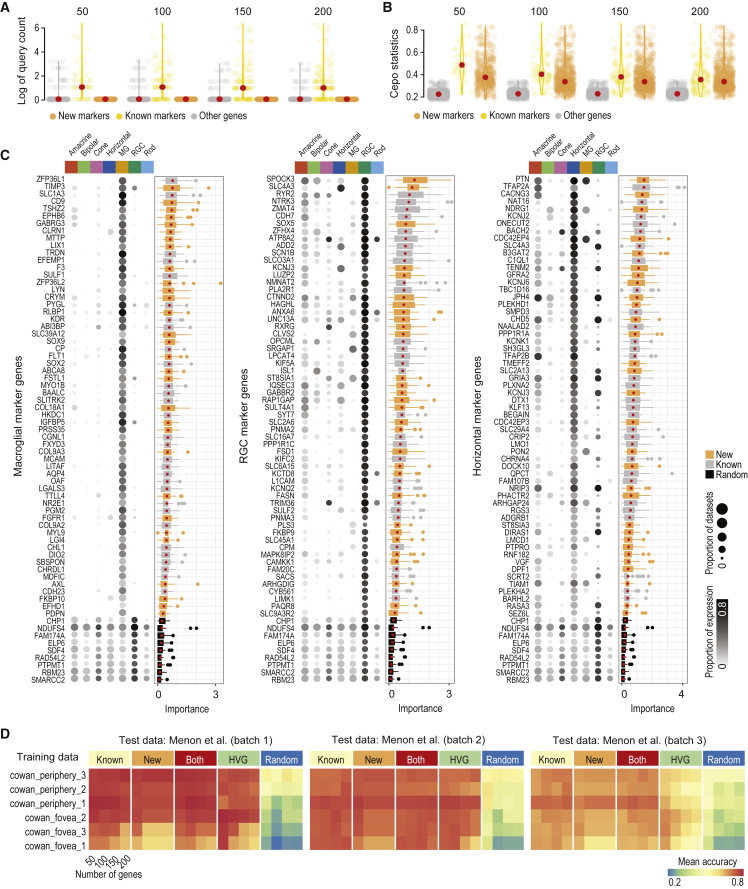
Identification and validation of novel cell-type-specific gene markers of the retina (A) Scatter violin plots of log of query count of the top 50, 100, 150, and 200 genes categorized into known or new genes. The scatterplot visualizes the PubMed queries for the results from all cell types and those from non-marker genes for comparison. (B) Scatter violin plots of the same query results as in (A) but of the respective Cepo statistics. (C) Cell-type-specific gene markers identified by Cepo. Proportion of cells expressing each marker in each cell type is represented by the gradient color and the proportion of datasets having each marker expressed is represented by the size of the balloons. Importance scores of gene markers are derived from random forest classification of cells using these markers. Novel markers are highlighted in orange and known markers are in gray. Randomly selected genes (in black) are included as controls. (D) Classification accuracy of independent test data (Menon et al., 2019) from *k*NN classifiers trained on each of the Cowan datasets using known or new gene markers or their combination, highly variable genes (HVG), and randomly selected genes.

To uncover potential new markers of cell types, we performed a systematic query search on PubMed on the genes highly ranked by Cepo (see supplemental information). We considered a gene as “known” if its query returned any publication or as “novel” if its query returned no searches (Figure 3A). Genes found to be neither a known nor a novel gene were defined as “others.” We found that, while many cell-identity genes uncovered by Cepo are known gene markers for their respective cell type, many genes that were not previously associated with the retinal cell types were ranked highly by their Cepo statistics (Figure 3B). Indeed, both known and new cell-type markers uncovered by Cepo showed expression specific to that cell type, whereas the randomly selected stable genes showed non-specific expression across all cell types, as demonstrated by the exclusive expression patterns of the known and new markers in Figures 3C and S3A. In agreement, known and new gene markers, but not the randomly selected stable genes, showed utility in classifying their respective cell types as indicated by their feature importance score computed from the random forest algorithm (Yang et al., 2021) (Figures 3A and S3B).

To further evaluate the utility of known and novel cell-identity genes in delineating the retinal cell types, we performed cell-type classification by training a *k*-nearest neighbor (*k*NN) classifier on different numbers of top genes ranked by Cepo and classifying an independent retina scRNA-seq dataset (Menon et al., 2019) (Figures 3D and S3B). We also assessed the classification accuracy using highly variable and randomly selected genes. We found that the known and new marker genes led to similar classification accuracy of cell types with an average >0.8. As expected, highly variable genes showed better than random classification accuracy but were much lower than Cepo-selected cell-identity genes. Last, the combination of known and new marker genes resulted in the best classification in most cases across all three batches of the data (Figure S3B). Taken together, these analyses support the discovery of new gene markers for each of the major retinal cell types (Table S1) and demonstrate their efficacy in delineating their respective cell types in the retina.

### Identifying genes associated with human retina maturation

**Figure 4:**
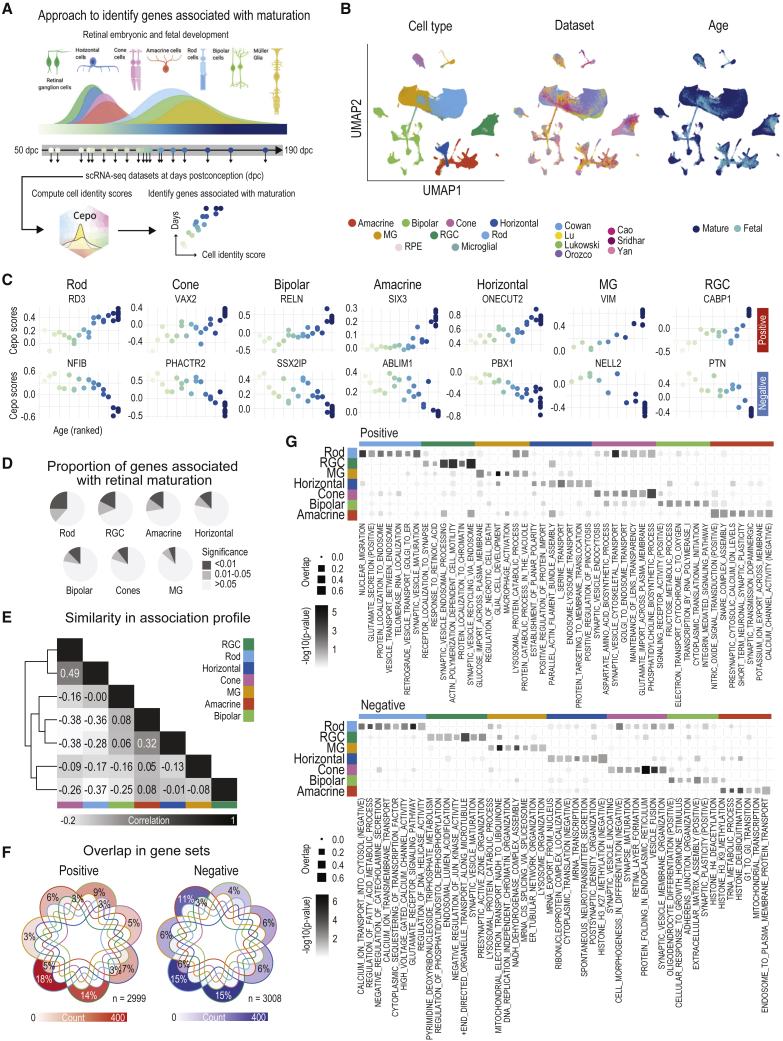
The retinal cell-identity scores uncover genes associated with retinal maturation (A) Schematic workflow to derive cell-type-specific maturation-associated genes. Scaling by day post-conception (dpc). (B) UMAP of cell transcriptome profiles combining both adult and fetal tissues. Cells are colored by their type (left), dataset (middle), and sample type (right). (C) Scatterplots of developmental age (x axis) and Cepo statistics (y axis). Points denote individual samples and are colored by their ranked developmental time point. The top and bottom show genes that are positively and negatively associated with age, respectively. (D) Proportions of highly significant, significant, and insignificant maturation-associated genes for each cell type. Highly significant, false discovery rate [FDR]-adjusted p <0.01; significant, FDR-adjusted p between 0.01 and 0.05; and insignificant, FDR-adjusted p >0.05. (E) Similarity in maturation association profiles between the cell types in terms of Pearson’s correlation coefficient. (F) Overlap among the positively (left) and negatively (right) significant genes (FDR-adjusted p <0.05). The total percentage of overlap is highlighted for intersections greater than 2% of overlaps. The color scale denotes the absolute number of genes in each gene set. (G) Enrichment of gene sets positively and negatively correlated with age.

Several recent studies have profiled the developing human eye using scRNA-seq (Cao et al., 2020; Lu et al., 2020). To characterize these fetal samples and identify genes that are associated with maturation of each retina cell type (Figure 4A), we curated these datasets, each profiling a wide range of developmental stages (Figure S4A), and combined this resource with the mature retinal atlas. The UMAP revealed that the major cell types of the retina clustered together (Figures 4B and S4B). In line with the developmental birth order of retinal neurons and the Müller glial, we observed that fetal samples from up to approximately 100 days post-conception contained high proportions of retinal ganglion cells, horizontal cells, and cones (in relation to rod cells) (Figure S4C) (Shiau et al., 2021). Rods, amacrine, bipolar, and Müller cells demonstrated increased proportions after the 100-day time point, in agreement with the knowledge that these cell types are late-born cells. The final proportions of the fetal and mature cells in the combined retinal atlas demonstrate the inclusion of both age groups in all the cell types (Figure S4D).

To discover genes associated with human retinal maturation, we investigated the correlation of cell-type-specific Cepo gene statistics and the developmental age of the retinal samples. This analysis therefore enabled the generation of cell-type-specific scores that denote whether a gene exhibits a gain or a loss in cell-type-specific expression over time and the discovery of genes associated with maturation (Figure 4C), many of which have been established in the literature as associated with retinal development and disease (Azadi et al., 2010; Sapkota et al., 2014; Sinha et al., 2016; Zhu et al., 2002). Cepo statistics were computed as previously described for each cell type and batch, and the clustering results from their pairwise correlation relationships were visualized as a heatmap (Figure S4E). We found that the cell types were associated with varying numbers and proportions of maturation-related genes (Figures 4D and S4F) and that these maturation profiles are highly cell-type specific. These findings not only demonstrate the presence of cell-type-specific maturation programs but also highlight the capacity of our Cepo statistics to identify highly cell-type-specific genes (Figures 4E and 4F). Collectively, these findings reveal that substantive re-wiring occurs during development, whereby the re-wiring leads to distinct gene expression patterns between cell types.

Next, we asked whether the genes associated with maturation of the major cell types of the retina represented those relevant to their respective cell types. To test this, we performed overrepresentation analyses using the gene sets shown in Figure S4F. Consistent with the neuronal identity of many of the retinal cell types, we observed that the most enriched pathways among the gene sets positively associated with retinal maturation were related to the formation and regulation of the synapses (Figure 4G). For example, consistent with the fact that amacrine cells are the dopaminergic neurons of the eye, a positive amacrine maturation profile was strongly enriched for the “regulation of synaptic transmission dopaminergic” pathway (Dacey, 1990).

### Benchmarking framework to evaluate diverse retinal organoid differentiation protocols

**Figure 5:**
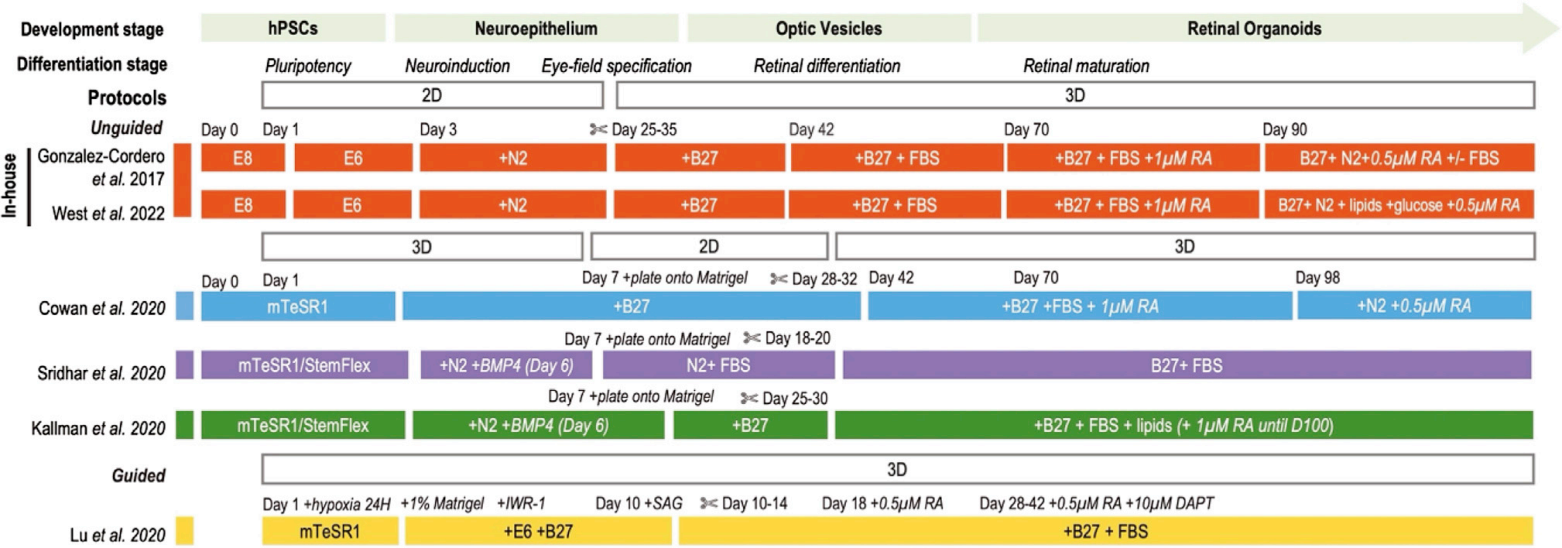
Protocols of human retinal organoids Overview of the culture systems for generating human retinal organoids. The schematic illustrates the timeline of retinal induction, differentiation, and maturation steps, outlining 2D and 3D stages, key supplements, and factors added to culture conditions across the published protocols. Abbreviations: hPSCs, human pluripotent stem cells; E8, Essential 8; E6, Essential 6; RA, retinoic acid; H, hour; DAPT, N-[N-(3,5-difluorophenacetyl)-*l*-alanyl]-S-phenyl glycine t-butyl ester) γ-secretase inhibitor; ✄, dissection/dislodgment.

**Figure 6:**
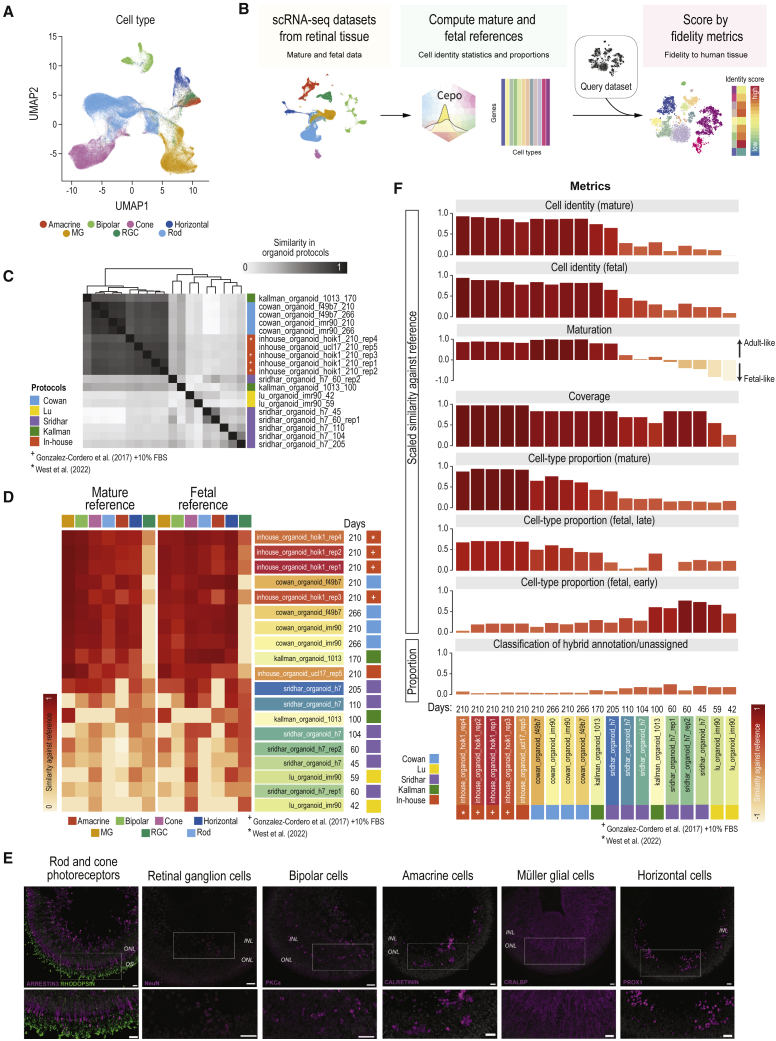
Benchmarking the human retinal organoid protocols in terms of their fidelity to the retinal tissue (A) UMAP of cell transcriptome profiles from human retinal organoids. (B) Schematic of the benchmarking procedure and evaluation metrics. (C) Similarity in cell-identity profiles between the scRNA-seq datasets generated from various organoid protocols. Similarity is measured in terms of Pearson’s correlation coefficient. (D) Benchmarking results of the fidelity of the individual cell types to either the mature or the fetal reference. The cell-identity metric gives a scaled score between 0 and 1, where 1 denotes a high capacity and 1 denotes a low capacity of the protocol to generate cell types that closely match their tissue counterparts. (E) Representative images of retinal cell types in human retinal organoids at 210 days of age. Abbreviations: ONL, outer nuclear layer; INL, inner nuclear layer; OS, outer segments. Scale bars: 20 μm. (F) Final benchmarking results of the human retinal organoid protocols ranked by the combined score from six evaluation metrics: cell identity (mature), cell identity (fetal), maturation, coverage, cell-type proportion (mature), and proportion of potential off-target cells. The scores, except the maturation and proportion off-targets, have been scaled from 0 to 1 within each evaluation metric, where a value of 1 denotes a high capacity of the protocol to mimic the reference and 0 denotes low capacity.

Recent advances have led to the development of several state-of-the-art protocols for generating human retinal organoids that largely resembles the endogenous retina (Figure 5) (Afanasyeva et al., 2021; Berber et al., 2021; Llonch et al., 2018; Mellough et al., 2019; Völkner et al., 2016; Zhang et al., 2021). We curated public single-cell transcriptomics datasets obtained from these protocols (Cowan et al., 2020; Kallman et al., 2020; Lu et al., 2020; Sridhar et al., 2020), as well as generating our in-house data (Gonzalez-Cordero et al., 2017; West et al., 2022) (Figures 6A and S5A–S5D). While functional and molecular studies have evaluated the efficacy of these organoid protocols for efficient and robust generation of retinal cell types, no studies have systematically evaluated these protocols by comparing their global cellular and molecular profiles with human retinal tissue.

To address this gap, we devised a framework to systematically assess these retinal organoid protocols. In brief, leveraging the mature and fetal retinal atlas, we derived references denoting the cell-identity scores, maturation, the cell-type proportions, and the cell-type coverage aspired for the organoids. Importantly, we implemented six metrics based on the retinal atlas that measure the capacity of the protocols (1) to mimic the cellular identities of the mature and (2) the fetal retinal tissue; (3) to recapitulate the developmental stage; (4) to generate the cell-type proportions found in the tissue; (5) to generate all the major cell types in the retina; and finally (6) to generate the smallest amount of off-target cells (Figure 6B). The full description of how the references were generated and the metrics were computed can be found in the supplemental information.

### Benchmarking the fidelity of human retinal organoids to the human tissue

Using this benchmarking framework, we evaluated the capacity of each protocol to generate cell types that closely mimic the human eye. We first evaluated the similarity between the protocols by comparing the cell-type identity scores generated for each dataset and batch. We found that our in-house-generated retinal organoids (Gonzalez-Cordero et al., 2017; West et al., 2022) and those of Cowan et al. (2020) demonstrated the highest degree of similarity and also the highest level of consistency between batches (Figure 6C). Next, for each of the datasets we computed the cell-identity metric to assess the fidelity of each cell type of the organoids against the mature and fetal references. Ordering the samples by their similarity to the reference revealed that the organoids derived from our in-house protocol closely followed by the Cowan protocol, consistently outperforming those at similar ages derived from other protocols in terms of achieving a high fidelity in cellular identity against both references (Figure 6D). Immunohistochemistry analysis indeed confirmed the presence of all the major retinal cell types in day 210 human retinal organoids (Figure 6E, n = 15 organoids; N = 3 differentiation batches; generated using West et al., 2022).

To perform a cross-laboratory and cross-protocol benchmarking of the human retinal organoids, we applied the six fidelity metrics to the organoid datasets. The cell-identity metric was computed as the average of the cell-type-specific scores described in Figure 6D. Among organoids grown for 100 days or more, the overall score showed that our protocols (Gonzalez-Cordero et al., 2017; West et al., 2022) generated retinal cell types most faithful to both mature and fetal tissue, closely followed by those from the Cowan protocol. The organoids from the Kallman and Sridhar protocols at similar culture periods (grown until 170 and 205 days, respectively) performed less in terms of the cell-identity metrics. While it is not appropriate to directly compare the organoids from the early and late culture stages, it is worth noting that these organoids show minimal resemblance to the fetal reference, suggesting either that progenitor cells present at these early time points of organoid are not captured in our fetal reference due to difference in age or that these organoids do not robustly recapitulate the fetal retinal cell types.

Next, the coverage metric was computed as the proportion of the major cell types of the retina present in the organoid data. We observed that our protocols achieved the highest coverage of the major cell types of the retina consistently across five batches that differed in organoid cell line, organoid batch, and in-house protocols (West et al., 2022; Gonzalez-Cordero et al., 2017, ±FBS) (Figure 6F). This includes retinal ganglion cells (RGCs), which are known to decrease in numbers with extended organoid culturing. We observed RGCs in our cultures, albeit in small numbers, making a small proportion of the total cells (Figure S5B). This was confirmed by the presence of THY1 (CD90)- and NeuN-positive cells in the 210-day-old organoids (Figures 6E and S6E). Furthermore, gene expression of *THY1* confirmed the specificity of this marker to RGCs (Figure S6F).

To investigate the developmental relevancy of the organoids, the maturation metric computed from all the individual cell-identity scores from the mature and fetal data was used. Briefly, we assessed the capacity of the organoids to either positively (more adult-like) or negatively (more fetal-like) correlate with the time-resolved cell-identity profiles as a proxy for their developmental relevancy (Figure S6A). Our findings show that young organoids (≤60 days) demonstrate a strong negative correlation against the age-ranked references, whereas organoids older than 170 days showed a strong correlation against the age-ranked references. Thus, the overall scores, which have been averaged for each cell type, show either a strong adult- or a strong fetal-like profile of organoids at the bookends of the protocols (Figure 6F).

The metric—the proportion of cell types—was computed from the mature and fetal reference, the latter of which is also subdivided into two references denoting early and late fetal maturation. We found that, again, our protocols excelled among the protocols in generating cell-type proportions that represent those found in the mature human eye and not the fetal eye (Figure 6F). Intriguingly, even though the early-stage organoids poorly recapitulated the cellular identities of the fetal tissue, we found that their cell-type proportions better reflected those of the fetal eye (Figure 6F).

The final metric, the proportion of off-targets, was computed for all the samples using scClassify, a multi-scale classification algorithm that identifies intermediate and unassigned cells (Lin et al., 2020). The average proportion of cells classified into either of these categories was used as the proxy for the generation of off-target cells, which is important to minimize when generating cells for transplantation. We show that, as expected, the mature organoids generated fewer off-target cells (<10%) than younger organoids, which may consist of cells undergoing development (Figure S6B), and that most protocols demonstrated a high capacity to generate on-target cells (Figure 6F).

Overall, our benchmarking study revealed that our in-house protocols (Gonzalez-Cordero et al., 2017; West et al., 2022) generate cell types of the retina with the highest fidelity to the human adult retina in terms of their cellular identity, cell-type proportion, and coverage (Figures 6F and S6C). To facilitate future benchmarking of PSC-derived human retinal organoids generated from various differentiation protocols, we have implemented our retinal cell-type identity map and the benchmarking framework as an online resource (https://shiny.maths.usyd.edu.au/Eikon/) where the fidelity and quality of organoids can be explored, assessed, and scored.

## Discussion

A key contribution of this study is the discovery of potential novel markers of the major retinal cell types and of retinogenesis. To elucidate this array of cell-type-specific markers, we integratively analyzed the scRNA-seq datasets of the fetal and the mature retina. This is now possible with the advent of single-cell technologies, which has enabled the extensive exploration of retinogenesis at a single-cell resolution. For example, recent studies have begun elucidating the trajectories of retinal-derived photoreceptor differentiation, highlighting markers that distinguish rod versus cone specification during development (Kallman et al., 2020). Our study takes advantage of the rich resource of scRNA-seq datasets of the developing and mature retina to interrogate genes that are associated with retinogenesis. We uncovered intrinsic differences in the maturation profile between all the major cell types of the retina and showed that these specification profiles were largely cell-type specific. While we anticipate that these genes can be used as a resource for further studies to investigate retinogenesis in development and disease, we note that future studies are required to validate these findings in *in vivo* and *in vitro* systems.

Another contribution is our effort to benchmark human retinal organoids generated by diverse differentiation protocols against the *in vivo* human retina. This has revealed that unguided human retinal organoids generated using our in-house differentiation protocol and that of Cowan et al. were top performers (Cowan et al., 2020; Gonzalez-Cordero et al., 2017; West et al., 2022). Notably, these two protocols have similar time courses of RA addition and supplementation. All differentiation protocols showed efficient generation of organoids; however, our 2D-3D is able to generate organoids from PSC confluence in a simpler workflow (Figure 5) (Gonzalez-Cordero et al., 2017; Reichman et al., 2014; West et al., 2022). Furthermore, we have recently shown that optimization of late-stage culture conditions with lipid supplementation enhances maturation of photoreceptor cells (West et al., 2022). Among the benchmarked protocols, our protocol generates cell types of the retina with the highest fidelity to the human retinal tissue across various evaluation metrics (Figures 6D–6F). These culture improvements possibly explain the presence of a small number of RGCs in the mature in-house organoids only.

While our benchmarking study highlighted that some of the protocols can successfully develop retinal organoids closely resembling many aspects of the human retina, it also highlighted remaining challenges. We observe that the organoids, in particular those collected at earlier maturation stages (50–170 days), do not fully recapitulate the developmental stages of the human retina (Figures 6D and 6F): a feature to be taken into consideration when investigating human eye development and retinogenesis using human retinal organoids. Furthermore, none of the protocols can currently generate retinal pigment epithelium (RPEs), which is a clear shortcoming of the protocols. Future benchmarking efforts will require the incorporation of studies that generate single-cell transcriptomic profiles of RPEs across development (Hu et al., 2019; Petrus-Reurer et al., 2022).

One of the key limitations of the study is that the fidelity metrics do not address important features of the retina, such as the profiles of the other omics layers, spatial patterning, retinal organoid functionality, and domain-specific characteristics. Recently, a few studies have begun profiling the retinal organoid using multi-modal single-cell technologies (Thomas et al., 2022; Wahle et al., 2022). The study by Thomas et al. mapped the *cis*-regulatory elements of the developing and mature human retina and showed that human retinal organoids are capable of emulating the DNA accessibility of the human retina (Thomas et al., 2022). The spatial organization of the retina is an important component to evaluate for tissues, like the retina, that have highly complex, ordered, and specialized structures. Toward this end, spatial transcriptomics that can profile the transcriptomes while retaining the spatial coordinates of the single cells would enable us to assess the capacity of protocols to generate organoids that are spatially organized to closely emulate that of *in vivo* tissue (Rao et al., 2021). Therefore, future studies will be required to develop computational methods that can quantify the reference spatial organization for each cell type of the retina and measure the fidelity of organoids to conform to the reference.

## Experimental procedures

### Resource availability

#### Corresponding author

Further information and requests for recourses and reagents should be directed to and will be fulfilled by the corresponding authors, Anai Gonzalez-Cordero (agonzalez-cordero@cmri.org.au) and Pengyi Yang (pengyi.yang@sydney.edu.au).

#### Materials availability

This study did not generate new unique reagents.

#### Data and code availability

The sequencing data generated in this study have been deposited in the Gene Expression Omnibus (GEO) under accession no. GSE201356. The code generated during this study is available upon reasonable request to one of the corresponding authors. Eikon (https://shiny.maths.usyd.edu.au/Eikon/) is available as an interactive web application to explore the fidelity of retinal organoids.

### Cell culture and retinal organoid generation

#### Human induced pluripotent stem cells

HPSI0314i-hoik_1 (RRID:CVCL_AE82) was obtained from ECCAC. UCLOOi017-A-1 was derived from healthy donor peripheral blood mononuclear cells (PBMCs) as described previously (Fernando et al., 2022). PBMCs were isolated using density gradient centrifugation. Briefly, 25 mL of whole blood diluted 1:1 with PBS was layered on top of 15 mL of Ficoll-Paque Premium and centrifuged with brake and accelerator off at 500*g* for 30 min, and the cloudy interphase containing PBMCs was collected. Two million cells were cultured for 6 days in hematopoietic expansion medium StemSpan H3000, with the addition of EPO, IL-3, dexamethasone, ascorbic acid, SCF, and IGF-1. Following expansion, 200,000 cells were nucleofected using an Amaxa 4D nucleofector with Addgene plasmids. The nucleofected cells were plated in a well of a six-well plate coated with Geltrex matrix and transitioned to Essential 8 medium.

#### Human induced pluripotent stem cell maintenance and differentiation

Cells were incubated at 37°C in 5% CO_2_. Human induced PSCs (hiPSCs) were grown and expanded under feeder-free conditions using Essential 8 medium (E8; Life Technologies) on six-well plates coated with Geltrex (Invitrogen) at a concentration of 1:100. The medium was replaced daily and cells were passaged at 70% confluency via 5–10 min of 37°C incubation with Versene solution (0.48 mM) (Life Technologies) to detach clumps of cells. Cell clumps were resuspended at a ratio of 1:6–1:12 in E8 with 10 μM ROCK inhibitor (Y-27632 dihydrochloride; Tocris) and seeded in fresh Geltrex-coated six-well plates. For differentiation, hiPSCs were grown to 90%–100% confluency.

#### Generation of retinal organoids from hiPSCs

Retinal organoids were differentiated as previously described (Gonzalez-Cordero et al., 2017; West et al., 2022) with some modifications. Briefly, at 90%–100% confluency (denotated day 1), hiPSC medium was replaced with Essential 6 (E6; Life Technologies) daily for 2 consecutive days. On day 3, E6 medium was replaced with pro-neural induction medium (PIM; Advanced DMEM/F12, 1× N2 supplement, 1.9 mM L-glutamine, 1× MEM-NEAA, 10% antibiotic-antimycotic [all Life Technologies]). Optic vesicles displaying neuroretinal epithelium were manually isolated using a needle under an EVOS XL microscope (Invitrogen) between days 25 and 35 and transitioned to 3D suspension culture in low-binding 96-well U-shaped plates and retinal differentiation medium (RDM; DMEM high glucose 68% v/v, Ham’s F-12 nutrient mix with GlutaMAX supplement 29% v/v, 1× B-27 supplement minus vitamin A, 10% antibiotic-antimycotic [All Life Technologies]). At day 42, RDM was replaced with RDM + factors (RDMF; DMEM high glucose 60% v/v, Ham’s F-12 nutrient mix with GlutaMAX supplement 26% v/v, 2× GlutaMAX supplement, 1× B-27 supplement minus vitamin A, 10% antibiotic-antimycotic) [all Life Technologies], FBS 10% v/v [Bovogen]). At day 70, retinal organoids were transferred into low-binding 24-well plates and the medium was replaced with ALT70 (Advanced DMEM/F-12 85% v/v, 10% FBS, 2× GlutaMAX supplement, 1× B-27 supplement minus vitamin A, 10% antibiotic-antimycotic [all Life Technologies], 100 μM taurine [Sigma Aldrich]) and supplemented with 1 μM all-*trans*-RA to enhance photoreceptor development. At day 90 and until the experimental endpoint, the medium was replaced with ALT90 (Advanced DMEM/F-12, 2× GlutaMAX supplement, 1× B-27 supplement minus vitamin A, 1× N2 supplement, 7 mM glucose, 10% antibiotic-antimycotic, 1× lipid mixture [all Life Technologies], 100 μM taurine [Sigma Aldrich], ±10% FBS [Bovogen]) and supplemented with 0.5 μM RA. The medium was replaced Monday, Wednesday, and Friday and the were cells maintained at 37°C in 5% CO_2_.

### Immunohistochemistry

Retinal organoids were washed with PBS, fixed for 40–60 min in 4% paraformaldehyde prior to incubation in 20% sucrose. After 210 days in culture, organoids were embedded in OCT, frozen in liquid nitrogen, and then cryosectioned at 14 μm thickness. Cryosections were blocked in 5% serum in blocking solution (1% bovine serum albumin in PBS with 0.1% Triton X) for 2 h. Primary antibody (Table S2) diluted in the blocking solution was incubated overnight at 4°C. Sections were washed with PBS and incubated with secondary antibody (Alexa Fluor 488, 546 secondary antibodies) at room temperature for 2 h. Sections were counterstained with DAPI.

## Author contributions

Conceptualization, H.J.K., A.G-C., and P.Y.; methodology, H.J.K., M.O-W., A.G-C., and P.Y.; investigation, H.J.K., D.K., A.G-C., and P.Y.; data generation, M.O-W., T.H.L., B.Y.L., R.V.J., A.G-C., and P.Y.; writing – original draft, H.J.K., M.O-W., A.G-C., and P.Y.; writing – review & editing, all authors; supervision, R.V.J., A.G-C., and P.Y.; funding acquisition, R.V.J., A.G-C., and P.Y.

## Supplemental information

Document S1. Figures S1–S6

Table S1. Cepo prioritised cell-type-specific cell identity genes of major retinal cell types

Table S2. Antibodies used for immunohistochemistry

Document S2. Article plus supplemental information
