# Supplementary material for: Comprehensive characterization of fetal and mature retinal cell identity to assess the fidelity of retinal organoids
Source: Stem Cell Reports. 2023 Jan 10;18(1):175–89. doi: 10.1016/j.stemcr.2022.12.002 (PMC9860116; doi:10.1016/j.stemcr.2022.12.002)
Supplement: Document S1. Figures S1–S6 [file mmc1.pdf]

Stem Cell Reports, Volume *18*

Supplemental Information

Comprehensive characterization of fetal and mature retinal cell identity to assess the fidelity of retinal organoids

Hani Jieun Kim, Michelle O'Hara-Wright, Daniel Kim, To Ha Loi, Benjamin Y. Lim, Robyn V. Jamieson, Anai Gonzalez-Cordero, and Pengyi Yang

Supplemental figures and legends

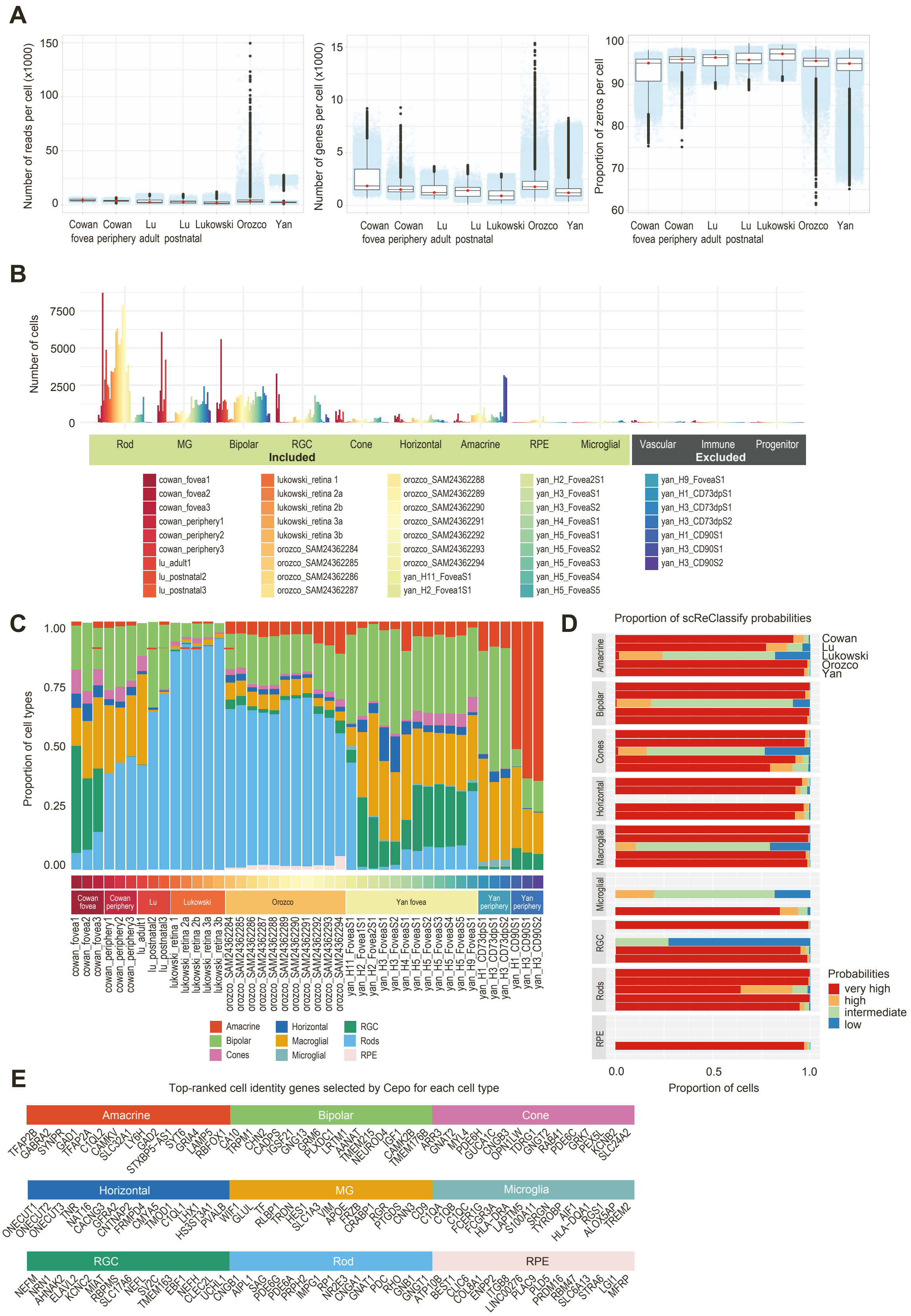

Figure S1. Quality control of retinal mature tissue scRNA-seq datasets. (A) Number of reads assigned, genes quantified, and proportion of zeros in each cell for each human retina dataset. **(B)**

Number of cells identified for each cell type in each dataset and batch. **(C)** Proportion of cell types (color coded) in each batch and dataset. Of note, samples from Yan et al. harvested from the periphery of the retina have been either depleted of rods (CD37dp) or depleted of rods and enriched for retinal ganglion cells (CD90). **(D)** Proportion of cells assigned with very high (>0.9), high (=< 0.9 and > 0.75), intermediate (<=0.75 and >0.5), or low (<= 0.5) probabilities according to scReClassify. scReClassify probabilities denote the confidence in cell type annotation where a higher probability denotes higher confidence. **(E)** Top ranked cell identity genes selected for each cell type by Cepo. Genes are ordered in the same order as the rows in the heatmap of **Figure1E**.

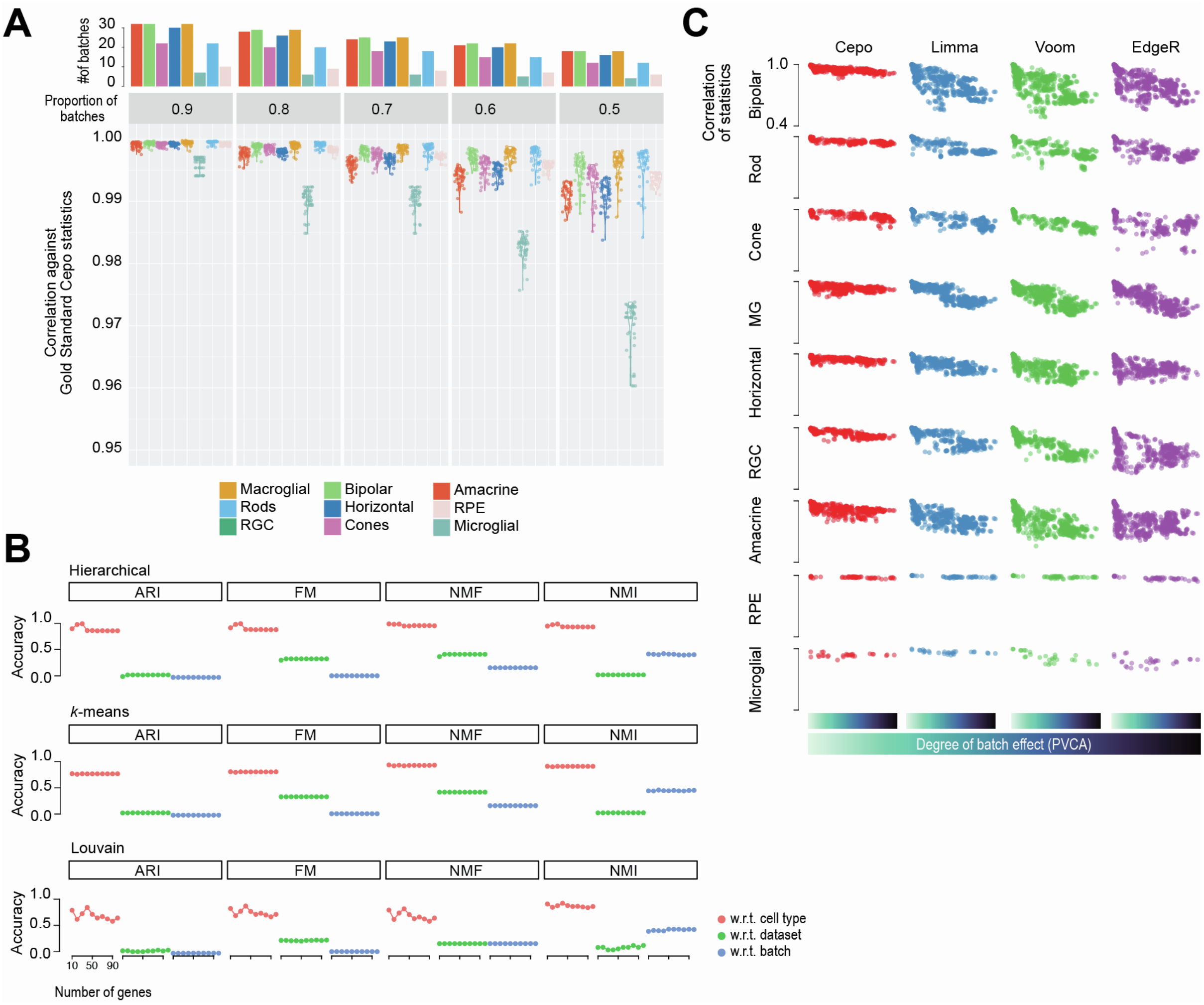

Figure S2. Assessment of batch effect on deriving cell-type-specific gene statistics. (A) Assessment of the stability of the gold standard Cepo statistics (i.e., the retinal cell identity map) with subsampling of the data at increasing rates (from 90 to 50% of the total number of batches). The Pearson's correlation coefficient of the correlation across overlapping genes (~15,000 genes) between the Cepo statistics from the full data and those derived from the subsampled data is plotted on the y-axis. For each subsampling study, the analyses were repeated 50 times using different seeds. **(B)** Clustering concordance quantified by adjusted rand index (ARI), Fowlkes Mallows index (FM), normalized mutual information (NMF), and normalized mutual information (NMI) with respect to cell type, dataset, and batch using hierarchical, Louvain, and k-means clustering algorithms. Number of genes used in clustering ranges from top 10 to 100 per cell type ranked by their Cepo statistics. **(C)** For each cell type, correlation of gene statistics calculated from Cepo, Limma, Voom, and EdgeR for each pair of datasets arranged by increasing batch effect as quantified by PVCA.

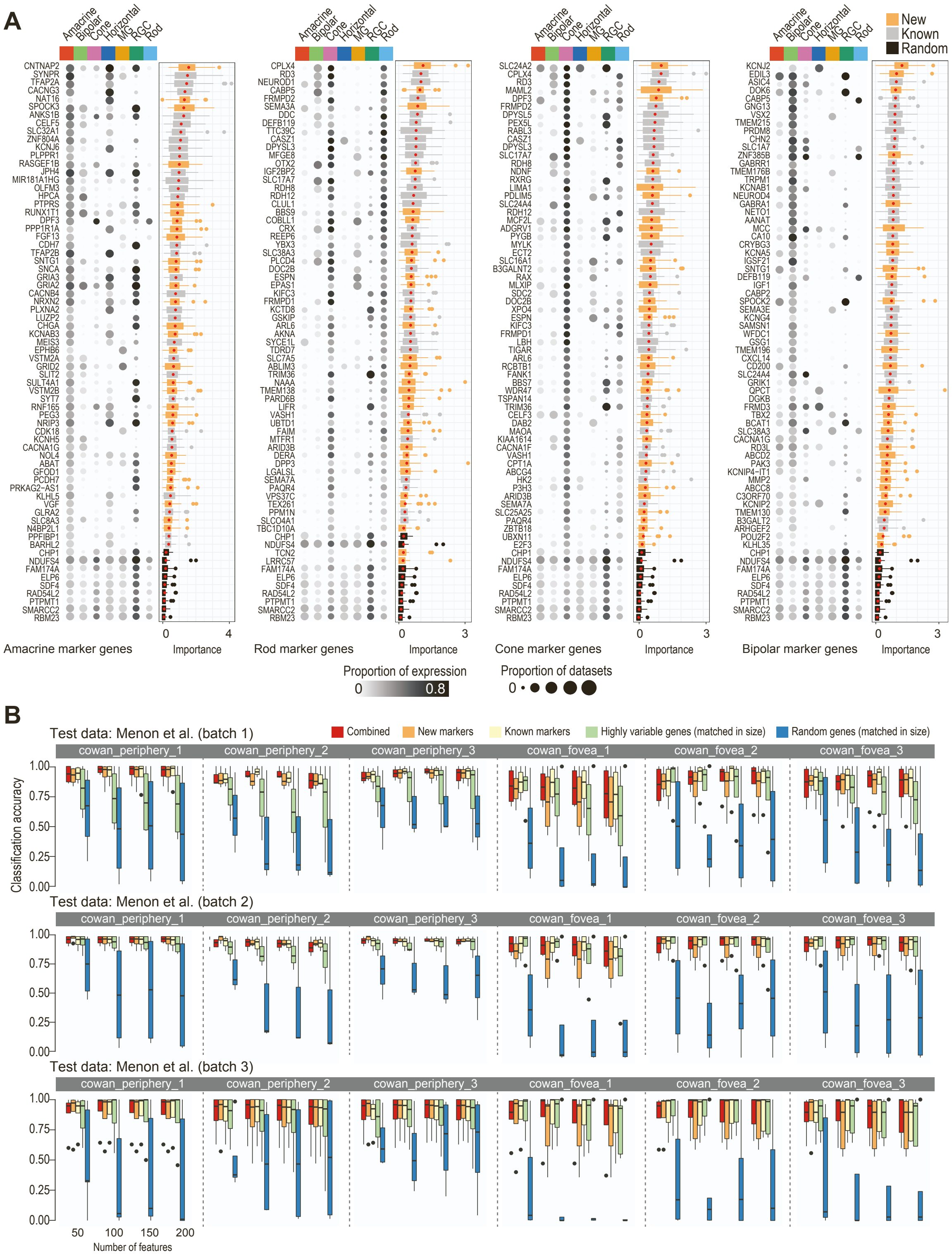

Figure S3. Cepo identification of cell-type-specific gene markers and their validation on external data. (A) Cell-type-specific gene markers identified by Cepo for Amacrine, Rods, Cone, and Bipolar cells. Proportion of cells expressing each marker in each cell type is represented by the gradient color and the proportion of datasets having each marker expressed is represented by the size of the balloons. Importance scores of gene markers are derived from random forest classification of cells using these markers. Novel markers are highlighted in orange and known markers are in gray.

Randomly selected genes (in black) are included as control. **(B)** Classification accuracy of each of the three batches of an independent test data (Menon et al.) from *k*NN classifiers trained on each of the five human retinal datasets and batches using various sets of gene markers (known, new, mixed, highly variable, and random).

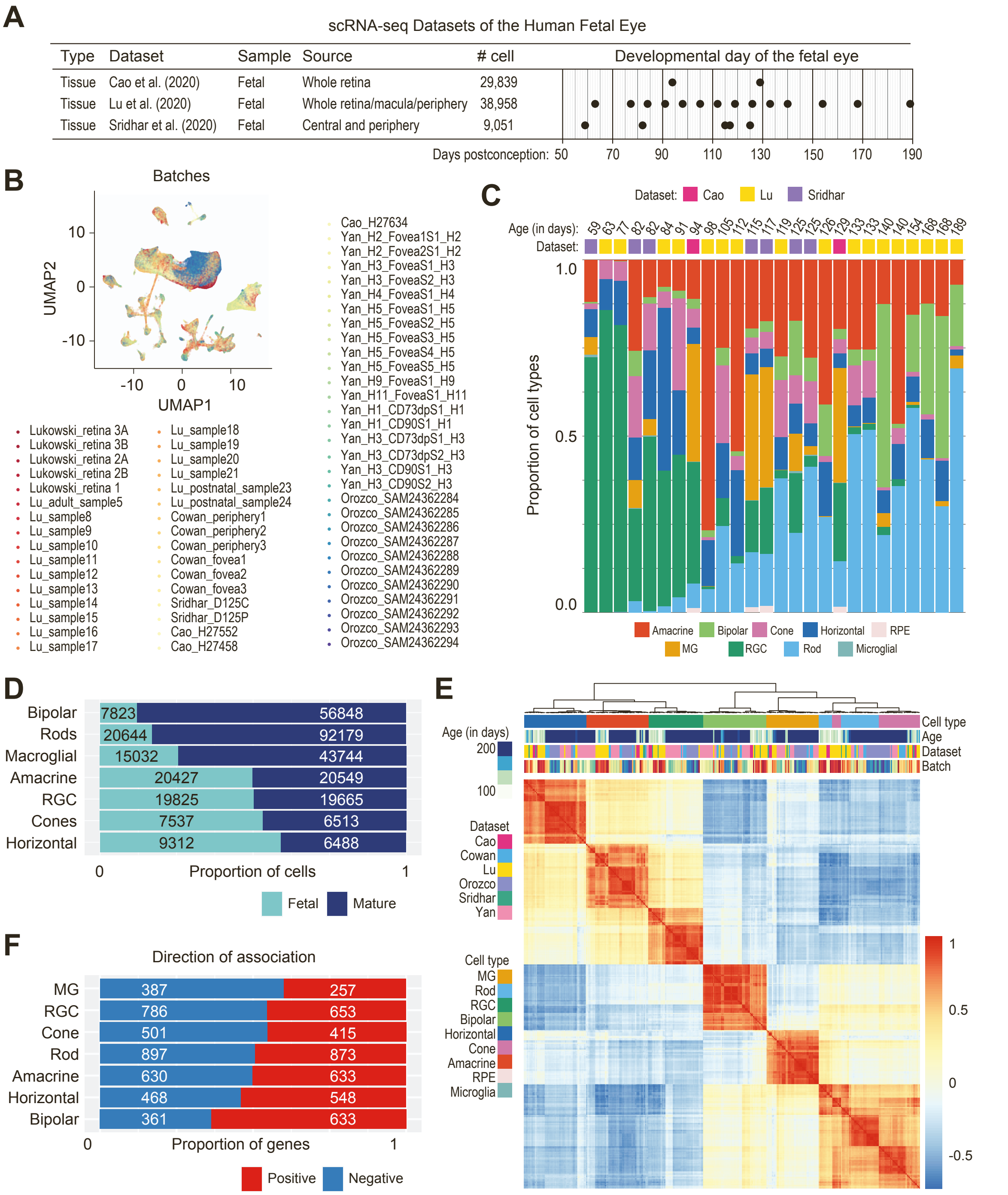

Figure S4. (A) Summary of scRNA-seq datasets collected from the retinal tissue. **(B)** UMAP representation of the transcriptomes of single cells from the mature and fetal atlas. Cells are coloured by their batch of origin. **(C)** Proportion of cell types (color coded) in each batch and dataset. **(D)** Proportion of mature and fetal cells (color coded) and their total number of cells in each cell type. **(E)** Correlation heatmap of cell-type-specific gene statistics generated from Cepo (Kim et al., 2021) for each cell type across datasets and batches. The heatmap is hierarchically clustered by the similarity of correlation profiles. **(F)** Proportional plot illustrating the direction of association among the significant genes (FDR-adjusted p-value < 0.05). The total number of genes in each set is noted in the plot.

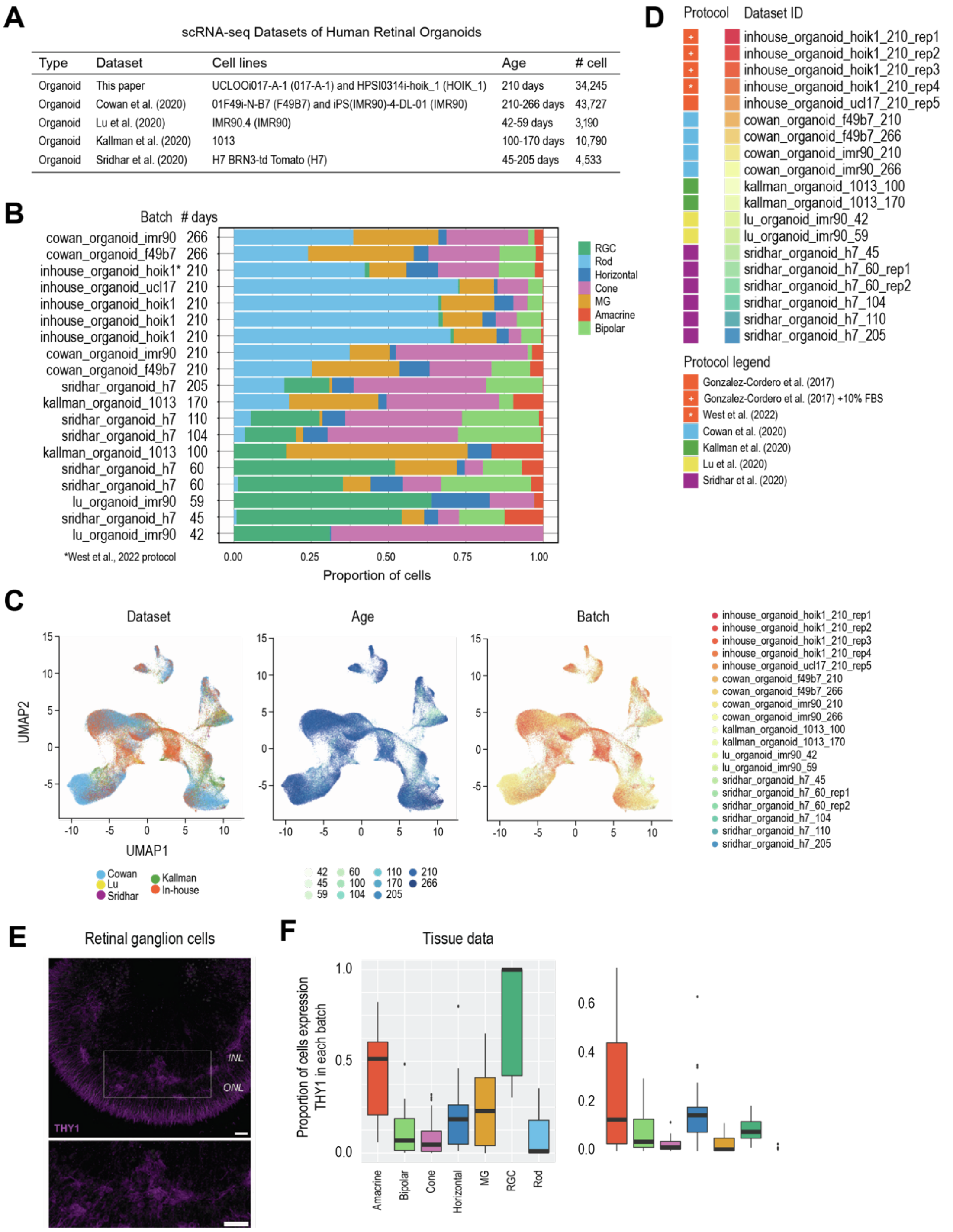

Figure S5. (A) Summary of scRNA-seq datasets collected from the retinal organoid studies. **(B)** Proportion of cell types (color-coded) and total number of cells in each batch and dataset. **(C)** UMAP representation of the transcriptomes of single cells from the organoid atlas. Cells are coloured by the dataset (left), the age until which the organoid was cultured (middle), and their origin of batch (right). **(D)** Legend showing the color annotations and dataset IDs of organoid scRNA-seq samples used in this study and their corresponding protocols. **(E)** Immunohistochemistry staining of Thy1, a marker of retinal ganglion cells, in a 210-day old organoid cultured using the West et al. 2022 protocol. **(F)** Boxplot showing the proportion of cells expressing Thy1 in each cell type (x-axis) and batch (individual data points) in the retina tissue samples (left panel) and organoids (right panel).

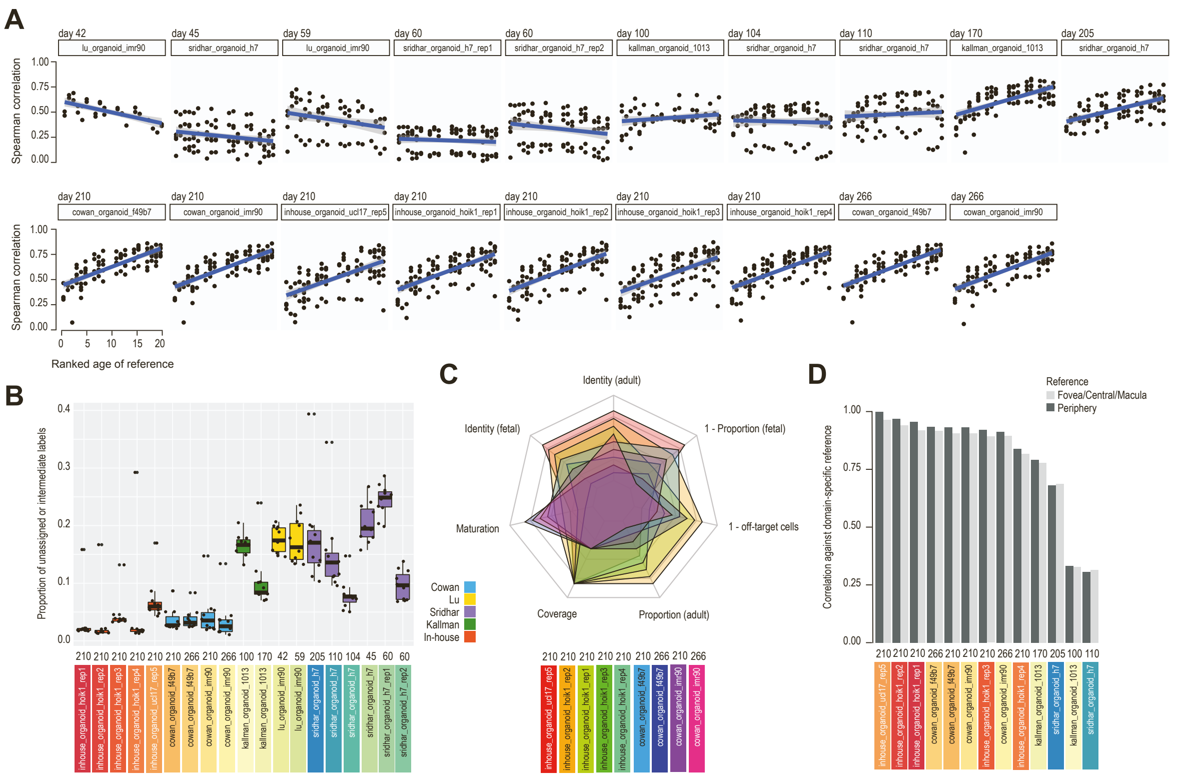

Figure S6. (**A**) Scatter plot of the correlation score of the cell identity scores of the organoid against each of the original reference scores from the tissue data. The y-axis denotes the Spearman correlation coefficient and the x-axis denotes the ranked developmental age of the original reference. The results from each organoid sample have been plotted separately. The scatter plot has been ordered from the youngest to the oldest organoid. (**B**) Boxplot showing the proportion of cells that were unassigned or showed hybrid cell-type annotations, indicative of off-target cells, in each organoid sample. Each datapoint denotes the replicates (n = 11) for each of the training data used in the scClassify framework. (**C**) Radar chart showing the order of ranks of organoids of each metric. (**D**) Barplot showing the correlation between the identities of the organoids (x-axis) and the domain-specific reference (Periphery or Fovea/Central/Macula). The average Spearman's correlation coefficient across the cell types is plotted on the y-axis. The barplots have been ordered by the highest to lowest correlation against the reference (from left to right).

Supplemental experimental procedures

QUANTIFICATION AND STATISTICAL ANALYSIS

Public single-cell RNA-seq datasets

Data collection

The raw gene-cell count matrices of scRNA-seq datasets were retrieved from the NCBI Gene Expression Omnibus (GEO) unless otherwise stated. The datasets are all related to the human retina, originating either from tissue or organoid and from mature or immature cells. Of note, we included only the datasets containing at least 10,000 cells to ensure enough cells in each batch and sample for the cell identity analysis.

Processing of scRNA-seq and filtering of cell types and suboptimal samples

All scRNA-seq libraries from human retinal tissue and organoids were integrated by Seurat (v4.0) (Hao et al., 2021) under the R (v4.1.1) environment. For each of the published datasets, the cell type labels from the original study were used. For datasets with unresolved cell type labels, such as "photoreceptors" and "AC/HC", we performed re-annotation of these subset of cells using scClassify (Lin et al., 2020) trained on the respective sample type (tissue or organoid) from the Lu et al. dataset (Lu et al., 2020), resolving them into either the Rods or Cones and Amacrine or Horizontal cells.

To establish a high-quality cell identity reference, we devised a framework to filter for suboptimal cells. Specifically, we performed a uniform cell type re-annotation procedure across all the tissue data using scReClassify (Kim et al., 2019), which is a semi-supervised learning method for assessing cell type annotation quality in the original classification from the scRNA-seq datasets. Only cells that received probabilities of correct assignment of greater than 0.9 were included in the study. Any datasets that resulted in greater than 50% of cells being mis-labeled were not included in the generation of the reference atlas.

Derivation of cell identity gene statistics and assessment of their concordance across batch

Computation of cell identity gene statistics

To derive the cell identity gene statistics for the human retina atlas, the count matrix of cell-gene variables were first log-transformed and normalized using the logNormCounts function from the scater package (McCarthy et al., 2017). The transformed and normalized data from each batch were subsequently analyzed using the Cepo package (Kim et al., 2021) for quantifying cell identity gene statistics for each major cell type based on the differential stability metric. For comparison, alternative methods (e.g., limma (Ritchie et al., 2015), voom (Law et al., 2014), edgeR (Robinson et al., 2010)) based on differential expression analysis were also used for calculating cell-type-specific gene statistics.

Evaluation of concordance of cell identity gene statistics after subsampling of the data

To evaluate the robustness of the cell identity statistics against subsampling of the data, we assessed the loss in Pearson's correlation between the cell identity statistics derived from the subsampled and those derived from the full data. The data was downsampled by randomly selecting 90% to 50% of the total number of batches. The analysis was repeated 50 times each with a different seed for each cell type.

Evaluation of concordance of cell identity gene statistics between batches

The evaluation of scRNA-seq datasets from multiple sources oftentimes requires technical noise to be removed. This so-called "batch effect" has been widely addressed through the development of several algorithms (Tran et al., 2020). However, the danger of overcorrection and restriction to a subset of features during the correction has hampered the widespread use of batch correction methods for downstream analysis. Therefore, an approach that bypasses the need to remove batch effects, does not compromise the biological signal, and retains many of the features is ideal to enable a comprehensive comparison between multiple atlases, often with a high degree of sparsity.

To this end, we assessed the fidelity of cell identity gene statistics between varying degrees of batch effect to determine the comparability of Cepo statistics across independent batches. A combinatorial assessment of cellular identity gene statistics concordance was performed between pairs of datasets. For all combinations of samples in the adult retinal tissue atlas, the extent of variance in principal component space contributed by batch and was quantified using the pvca package in R (Bushel,

2021). Briefly, principal variance component analysis fits a mixed linear model using the factor of interest, batch source, as an independent random effect to the selected principal components. Using the eigenvalues as weights, the associated variations of factors are standardized and the magnitude of the source of variability is presented as a proportion of the total variance. Therefore, a greater proportion of total variance denotes the presence of greater batch effect between the dataset pair.

To evaluate the concordance in cell identity gene statistics between the batches, the Cepo statistics were computed for each gene and for each cell type independently within each batch. Then for each batch pair, the concordance of cell identity was measured as the Pearson's coefficient of the correlation between the Cepo statistics. Finally, the relationship between the concordance and the degree of batch effect was examined. For comparison, cell-type-specific gene statistics from Limma, Voom, and edgeR were also analyzed using the same procedure.

Clustering of cell identity gene statistics and evaluation of batch mixing

To evaluate the consistency of the cell identity gene statistics derived for each cell type, clustering was performed on these statistics derived from different batches and datasets and then the degree of batch mixing was determined for three sources of variation: cell type, dataset source, and batch source. Ideally, cell identity gene statistics should reflect the biological identity of the cells and therefore the source of variation should solely arise from cell type identity and not from batch or dataset source.

To generate the cluster, three clustering algorithms (hierarchical clustering, k-means clustering, and Louvain clustering were performed. For each of the sources of variation, the resolution of the hierarchical and k-means clustering was set by controlling the parameter, number of trees or $centers$, respectively, to equal the number of cell types (7), the number of datasets (3), or the number of batches (14) present in the comparison. For the Louvain clustering wherein setting the exact cluster number is infeasible, the resolution was controlled by setting differing values of k when building the shared nearest neighbor graph (cell type, k = 5; dataset, k = 8; and batch, k = 2).

To assess the clustering performance of Cepo-derived cell identity gene statistics, we used the adjusted rand index (ARI) or the normalized mutual information (NMI) to evaluate the concordance of clustering results with respect to the cell type labels, batch, and dataset source, denoted respectively as either $ARI_{cell\ Types}$, $ARI_{dataset}$, and ARI_{batch} or $NMI_{cell\ Types}$, $NMI_{dataset}$, and NMI_{batch}. Considering that the Cepo-derived gene statistics are partitioned into different classes with respect to cell type labels, batch, or dataset source, let a be the number of pairs of samples partitioned into the same class by a clustering method, b be the number of pairs of samples partitioned into the same cluster but in fact belong to different classes, c be the number of pairs of samples partitioned into different clusters but belong to the same class, and d be the number of pairs of samples from different classes partitioned into different clusters. Then the ARI is calculated as follows:

$$ARI = 2(ad - bc)/(a+b)(b+d) + (a+c)(c+d)$$

Considering that the Cepo statistics are partitioned into different classes with respect to cell type labels, batch, or dataset source, let Y be the clustering outcome by a clustering method and C be the original labels of the different classes. Given that Y and C are the partitions of the same data, the overlaps between the two random variables can be counted and represented as a contingency table. Using information theory to measure agreement between the partitions and maximum likelihood estimation, the empirical joint distributions of clusterings Y and C are measured. Therefore, using the probabilities that an element falls into a given cluster, the entropy for clusterings Y and C, $H(Y)$ and $H(C)$, respectively, and the mutual information $I(Y;C)$ can be calculated. Then the NMI is calculated as follows:

$$NMI(Y,C) = 2 * I(Y;C)/H(Y) + H(C)$$

Identification of novel markers of cell type

Categorization of known and unknown cell-type markers

To systematically categorize a predicted marker gene as either already described in the literature or a new cell-type marker, an advanced PubMed query was performed using the R package easyPudMed (Fantini, 2019) for each of the top 500 marker genes. For each cell type, the advanced query consisted of three search terms: 1) the name of the candidate gene of interest; 2) the cell type of

interest; 3) and the term “RETINA”, all combined with the operator “AND”. The search terms covered “All Fields” of the possible search items. Any genes for which the PubMed search did not return a publication were categorized as a novel marker gene for the cell type of interest. In contrast, any genes for which the PubMed search did return at least one publication were considered known marker genes for the cell type of interest. One of the limitations of the PubMed query is that the individual publications returned as associated with a gene and cell type combination were not individually assessed for false positive result. Nevertheless, this approach provides a systematic and fast means to screen for novel markers, overcoming the need to manually survey the literature for all combinations of genes and cell types.

Quantification of feature importance

To demonstrate the importance of the potential marker genes, we performed feature selection analysis based on the random forest classifier (Breiman, 2001). The Gini index, which measures how important a selected feature is when training the random forest classifier, was used as a proxy for feature importance. To build the random forest classifier, the single-cell transcriptomes were subjected to random stratified sampling to 30% of its original size, and then pseudo-bulk transcriptomes for each cell type were generated by taking the mean expression of the genes. Approximately 350 pseudo-bulk transcriptomes were generated by repeating the sampling procedure 50 times. The random forest classifier was trained using these transcriptomes. As control, 10 cell-type invariant genes, as determined by their Cepo statistics, were included. A separate random forest classifier was built for each batch. Finally, the values for the mean decrease in Gini were extracted from the classifiers and visualized as a boxplot.

Classification of test data using known and novel gene markers

To further support the utility of the identified genes as novel markers of their respective cell types, we performed classification of single-cell transcriptomes of the human retina from an independent study (Menon et al., 2019), which was not included in the datasets used to derive the cell identity gene statistics. The *k*-nearest neighbor (*k*NN) classifiers were trained by varying the following four conditions:

Training data. Single-cell transcriptomes from six batches of data from the Cowan et al. (2021) study were used. The three batches of single-cell transcriptomes were each derived from either the periphery or the fovea.

Testing data. Single-cell transcriptomes from three batches of data from the Menon et al. (2019) study were used. The scRNA-seq data were derived from human retina tissue from either the macula in the central retina or a region of the mid-peripheral retina. Of note, the retina was mechanically separated from the retinal pigment epithelium-choroid.

Number of gene markers. The number of gene markers included in the training.

Category of gene markers. The top gene markers were derived from five categories: (1) gene markers categorized as known; (2) gene markers categorized as novel; (3) a gene set with both known and new markers; (4) highly variable genes; and (5) randomly selected genes. For gene sets 1-3, the top genes were ordered by Cepo statistics. For gene set 4, the top genes were ordered by the FDR-adjusted p-values computed from fitting a trend on the variance and mean of the log gene expression values.

Finally, the number of neighbors was set to k = 3 for all the kNN classifiers, and the final classification accuracy calculated for each cell type as follows:

$$Accuracy_{celltype} = Number\ of\ correct\ prediction_{celltype}\ /\ Total\ number\ of\ predictions_{celltype}$$

Identification of maturation-associated genes in retinal development

Determination of genes associated with maturation

To determine the genes associated with maturation, the cell-type-specific Cepo statistics derived for all the batches originating from the fetal and mature retinal atlas were used. Specifically, for each gene and each cell type, we computed the Spearman correlation coefficient as a function of the change in its Cepo statistics in that cell type over developmental age.

Measurement of similarity of maturation-association profiles between cell types

To investigate the similarity in the profile of maturation between cell types, we performed hierarchical clustering on the pairwise correlation matrix of the Spearman coefficient statistics on all the genes

found to be significantly associated with maturation (FDR-adjusted p-value < 0.05) in at least one cell type. The similarity is visualized as a clustered heatmap where 1 denotes complete positive correlation and -1 denotes complete negative correlation.

Gene set over-representation analysis of maturation-associated genes

Gene set over-representation analysis was performed on the genes significantly associated with maturation. Significance in association was determined as the FDR-adjusted p-value lower than 0.05. The gene set enrichment analysis was performed for each cell type and on either the gene sets positively or negatively associated with maturation. Using the GO terms related to biological processes from the C5 ontology gene set from the MSigDB collection (Liberzon et al., 2011), we assessed the over-representation of these gene sets among the maturation-associated genes using the fgsea R package (Korotkevich et al., 2022).

Analysis and benchmarking of retinal organoid protocols

Similarity of organoid protocols to one another

To evaluate the closeness of the organoid protocols to one another, we first performed Cepo analysis on the individual batches of the organoid datasets as in subsection “Computation of cell identity gene statistics” to derive cell identity gene statistics for each of the major cell types in each batch. Then, intersecting on the genes that are commonly found in all 19 batches, we aggregated the Cepo-derived gene statistics into a single vector. The similarity of cell identity profiles between the batches was evaluated in terms of the Pearson’s correlation coefficient between the aggregated statistics.

Evaluation of the fidelity of organoids to the human tissue

To evaluate the fidelity of organoids to the human tissue, we generated the following six evaluation metrics:

(1) **The cell-type-specific similarity against the cell identity retina reference**

The cell-type-specific similarity of the query organoid against the cell identity retina reference resolved by mature and developmental cells were computed. The average Cepo statistics from the mature and the developmental cell types were taken as the reference. Then, to

compute the cell-type-specific similarity of the query data, we calculated the Pearson's correlation coefficient between the Cepo statistics derived for the query data and each of the references for each cell type.

(2) The overall similarity against the cell identity retina reference

The averaged score of the cell-type-specific similarities from (1) were computed for each protocol across cell types to generate the overall cell identity score. As in (1), a higher score denotes stronger fidelity to the retinal reference and a lower score denotes weaker fidelity.

(3) The coverage of cell types

The coverage of cell types generated from the organoid protocol was calculated. The expected retinal cell types are cones, rods, Müller glial, ganglion, bipolar, horizontal, and amacrine cells. Protocols with the capacity to generate all cell types were assigned a score of 1, whereas those with nil capacity to generate the expected cell types were assigned a score of 0.

(4) Maturation profile

The developmental phase relevancy of the organoid was calculated by measuring the capacity of organoids to recapitulate the developmental change across time. Specifically, by computing the Spearman's correlation between the concordance between the cell identity scores of the organoid protocols and all the individual cell identity scores from the tissue adult and fetal data ordered by time, the final score reflects whether the organoids are more adult-like or fetal-like. This was performed for each cell type and final score was generated by averaging the results. A protocol with a high positive score denotes a more adult-like profile, whereas those with a high negative score denotes a more fetal-like profile.

(5) The concordance in cell-type proportion with the retina reference

The concordance against the proportion of cell types expected in the retina was measured using the averaged proportion profiles of the mature and fetal retina tissue datasets. To account for the differences in cell-type proportions that result from early and late-born cell types in early and late retinogenesis, the fetal proportional reference was sub-categorized into two time points (early [<97 days] and late [>97 days]). The intraclass correlation coefficient (ICC) for oneway models was used to compute an index of consistency of the proportions (Gamer et al.). A protocol with a high ICC reflects a high capacity to reciprocate the

proportions found in the real tissue, whereas those with a low ICC reflect those with a low capacity.

(6) **Proportion of off-target cells**

The proportion of potential off-target cells in the data was measured using scClassify (Lin et al., 2020). We first constructed a cell type hierarchical tree using HOPACH using the Orozco et al. samples as our reference dataset and used the weighted KNN classifier to assign each cell to a cell type. This procedure is repeated for the 11 individual batches in the reference data. The Limma package was used to select the top 50 features. A key feature of scClassify is that 1) it does not force a cell to be assigned to a cell type and 2) it allows cells to be annotated as an intermediate cell type (i.e., labelled as a hybrid cell from the non-terminal node of the hierarchy). Thus, any cells assigned by scClassify as an “unassigned” or “intermediate” cell type were considered potential off-target cells. A protocol with a low proportion of off-target cells reflects a high capacity to generate on-target cells, whereas those with a high proportion of off-target cells reflect low capacity to generate the in vivo cell types of the retina.

For each metric, except the proportion of off-targets, we then aggregated the results for all the benchmarked protocols and rescaled the score to a range of [0, 1]. Finally, equally weighting these metrics, the average of the overall cell identity (mature and fetal), the coverage, the cell-type proportion (mature), maturation, 1 minus proportion of off-targets, and 1 minus the cell-type proportion (fetal, early) was taken to generate a final score. This score was used to benchmark the retinal organoid protocols in terms of their fidelity to the human retinal tissue. Finally, whilst not included in the final metric, we generated the cell identity reference for each cell type for the central (including the fovea and macula) and periphery adult retina generated from the Cowan et al. and Yan et al. samples.

Single-cell RNA sequencing of retinal organoids and human retina

Dissociation of organoids into single cells

Five independent organoid batches were derived for both HPSI0314i-hoik_1 and UCLOOi017-A-1 hiPSC lines. One organoid was dissociated per sample. Retinal organoids were dissociated into

single cell suspension using the Neurosphere Dissociation Kit (P) (Miltenyi Biotec). Enzymatic digestion was performed as per manufacturer protocol for 10 minutes at 37°C with intermittent agitation, followed by gentle mechanical dissociation with a p1000 pipette, and a further 5 minute 37°C incubation. The cell suspension was passed through a p200 to ensure single cell dissociation before the enzymatic reaction was stopped by washing with HBSS. The cell suspension was filtered through MACS SmartStrainer 30µm (Miltenyi Biotec) before being pelleted by centrifugation at 400*g* for 10 minutes at room temperature. The cell pellet was resuspended in ALT90 and maintained on ice.

Single cell RNA-sequencing

Each single cell suspension of dissociated retinal organoid was assessed for viability using 0.4% Trypan Blue staining on a Countess II Automated Cell Counter (Invitrogen) and concentration was adjusted to 1000 cells/µl. Cell suspension was loaded on a single-cell-B Chip (10X Genomics) for a target output of 10,000 cells per sample. Single-cell droplet capture was performed on the Chromium Controller (10X Genomics). cDNA library preparation was performed in accordance with the Single-Cell 3' v3 protocol. Libraries were evaluated for fragment size and concentration using Agilent HSD5000 ScreenTape System. Samples were sequenced on an Illumina NovaSeq6000 instrument according to manufacturer's instructions (Illumina). Sequencing was carried out using 2×150 paired-end (PE) configuration with a sequencing depth of 40,000 reads per cell. The sequences were processed by GENEWIZ, China.

Analysis of in-house single-cell RNA sequencing data generated from retinal organoids

Read alignment and expression count table generation

From the sequencing results of the 10x Chromium experiments, the unique molecular identifiers, cell barcodes, and the genomic reads were extracted using Cell Ranger with default parameters (v3.1, 10x Genomics). The extracted reads were aligned against the annotated human genome, including the protein and non-coding transcripts (GRCh38, GENCODE v27). The reads with the same cell barcode and unique molecular identifier were collapsed to a unique transcript, generating the count matrix where columns correspond to single cells and rows correspond to transcripts. To remove potentially empty droplets with ambient RNA, the emptyDrops function from the DropletUtils package

was used. Droplets with significantly non-ambient profiles were called at a false discovery rate of 1%, applying the Benjamini-Hochberg correction to the Monte Carlo p-values to correct for multiple testing. Next, to remove potentially unhealthy or suboptimal cells, cell filtering was performed using the number of reads, the proportion of genes expressed, and the fraction of mitochondrial reads as criteria. Specifically, as cells with greater than 10,000 reads, 99% of genes not expressed, and 25% of mitochondrial gene expression were removed. Transcripts from mitochondrial- and ribosomal-protein coding genes were discarded for downstream analyses such as embedding and clustering, because they are typically known to be highly expressed irrespective of biological identity.

Doublet detection and filtering

The presence of multiplets in single-cell data can arise from incomplete dissociation of single cells meaning that more than one cell can be encapsulated in GEMs. DoubletFinder, an algorithm to detect multiplets in single-cell data, was used to remove potential doublets or multiplets from each biological batch at a threshold of 5.0% (McGinnis et al., 2019).

Integration and clustering

Embedding transcriptomes into a shared latent space

To embed the single-cell transcriptomes into a shared latent space, for each batch the count matrix was first normalized to the total number of reads and then factored by a 10,000 scaling factor. Then the top 2,000 features, among the top 1,000 highly variable features determined through variance stabilizing transformation, were prioritized by their variance across all the scRNA-seq batches. Next, the cell pairwise anchor correspondences between different single-cell transcriptome batches were identified with 30-dimensional spaces from reciprocal principal component analysis (Hao et al., 2021). Using these anchors, the scRNA-seq datasets were integrated and transformed into a shared space. Gene expression values were scaled for each gene across all integrated cells and used for principal component analysis (PCA). For the integration of the organoid datasets, $k.filter$ and $k.weight$ were set to 160 and 90, respectively, to accommodate the integration of datasets with fewer than 200 cells.

To generate the embeddings containing the single-cell transcriptomes derived from the mature tissue, the single cells were embedded into two-dimensional UMAP space by using the first 15 principal

components (PCs). To generate the embeddings containing the single-cell transcriptomes derived from the mature and development tissue combined, the single cells were embedded into two-dimensional UMAP space by using the first 30 principal components (PCs). Finally, to generate the embeddings containing the single-cell transcriptomes derived from the retinal organoids, the single cells were embedded into two-dimensional UMAP space by using the first 15 principal components (PCs).

Clustering and classification of in-house datasets

To cluster the single cells from the organoid datasets, the shared nearest neighbor graph was constructed on the first 30 PCs of the shared embedding using the default arguments of the FindNeighbors function in the Seurat package. Then the Louvain clustering algorithm with resolution equal to 1.1 was used to cluster the single cells. Classification of the single cells from the in-house datasets was performed by assigning them to the cell type that according to the labels of the public datasets that most dominate the cluster assigned to the cell of interest.

Development of the Eikon software

We implemented Eikon, an interactive web tool to facilitate the assessment of the fidelity of retinal organoids to the *in vivo* retinal tissue. Eikon accepts a SingleCellExperiment object (Lun et al., 2022). Multiple parameters can be specified in Eikon, including the assay, age of samples, and whether normalization is required. Users can customize the visualization plots using the provided options and all key visualizations are downloadable. Specifically, Eikon outputs three key visualizations, including several correlation heatmaps, reduced dimension plots, and an interactive table of Cepo statistics for each retinal cell type contained in the query data. The correlation heatmaps display the correlation cell identity scores between the query and reference datasets, allowing users to assess the fidelity of their data in a visually intuitive manner. Importantly, the query data can be compared to all or specific developmental stages of the reference dataset. PCA, UMAP, and t-SNE plots are also displayed and can be coloured by variables contained in the query data such as the proportion of zeroes and UMIs. Additionally, plots can be coloured according to the expression levels of a particular gene of interest which can be found using an interactive table displaying the Cepo statistics for each gene of each retinal cell type in the query data.

REFERENCES

Breiman, L. (2001). Random Forests. Machine Learning *45*, 5–32.

Fantini, D. (2019). easyPubMed: Search and Retrieve Scientific Publication Records from PubMed.

Gamer, M., Lemon, J., Fellows, I., and Puspendra, S. irr: Various Coefficients of Interrater Reliability and Agreement version 0.84.1 from CRAN.

Hao, Y., Hao, S., Andersen-Nissen, E., Mauck, W.M., Zheng, S., Butler, A., Lee, M.J., Wilk, A.J., Darby, C., Zager, M., et al. (2021). Integrated analysis of multimodal single-cell data. Cell *184*, 3573-3587.e29.

Kim, T., Lo, K., Geddes, T.A., Kim, H.J., Yang, J.Y.H., and Yang, P. (2019). scReClassify: post hoc cell type classification of single-cell rNA-seq data. BMC Genomics *20*, 913.

Korotkevich, G., Sukhov, V., Budin, N., and Sergushichev, A. (2022). fgsea: Fast Gene Set Enrichment Analysis (Bioconductor version: Release (3.15)).

Law, C.W., Chen, Y., Shi, W., and Smyth, G.K. (2014). voom: precision weights unlock linear model analysis tools for RNA-seq read counts. Genome Biology *15*, R29.

Liberzon, A., Subramanian, A., Pinchback, R., Thorvaldsdottir, H., Tamayo, P., and Mesirov, J.P. (2011). Molecular signatures database (MSigDB) 3.0. Bioinformatics *27*, 1739–1740.

Lin, Y., Cao, Y., Kim, H.J., Salim, A., Speed, T.P., Lin, D.M., Yang, P., and Yang, J.Y.H. (2020). scClassify: sample size estimation and multiscale classification of cells using single and multiple reference. Mol Syst Biol *16*, e9389.

Lu, Y., Shiau, F., Yi, W., Lu, S., Wu, Q., Pearson, J.D., Kallman, A., Zhong, S., Hoang, T., Zuo, Z., et al. (2020). Single-Cell Analysis of Human Retina Identifies Evolutionarily Conserved and Species-Specific Mechanisms Controlling Development. Dev Cell *53*, 473-491.e9.

Lun, A., Risso, D., Korthauer, K., and Rue-Albrecht, K. (2022). SingleCellExperiment: S4 Classes for Single Cell Data (Bioconductor version: Release (3.15)).

McCarthy, D.J., Campbell, K.R., Lun, A.T.L., and Wills, Q.F. (2017). Scater: pre-processing, quality control, normalization and visualization of single-cell RNA-seq data in R. Bioinformatics *33*, 1179–1186.

Menon, M., Mohammadi, S., Davila-Velderrain, J., Goods, B.A., Cadwell, T.D., Xing, Y., Stemmer-Rachamimov, A., Shalek, A.K., Love, J.C., Kellis, M., et al. (2019). Single-cell transcriptomic atlas of the human retina identifies cell types associated with age-related macular degeneration. Nat Commun *10*, 4902.

Ritchie, M.E., Phipson, B., Wu, D., Hu, Y., Law, C.W., Shi, W., and Smyth, G.K. (2015). limma powers differential expression analyses for RNA-sequencing and microarray studies. Nucleic Acids Research *43*, e47.

Robinson, M.D., McCarthy, D.J., and Smyth, G.K. (2010). edgeR: a Bioconductor package for differential expression analysis of digital gene expression data. Bioinformatics *26*, 139–140.

Tran, H.T.N., Ang, K.S., Chevrier, M., Zhang, X., Lee, N.Y.S., Goh, M., and Chen, J. (2020). A benchmark of batch-effect correction methods for single-cell RNA sequencing data. Genome Biology *21*, 12.
