# Supplementary material for: Comprehensive characterization of fetal and mature retinal cell identity to assess the fidelity of retinal organoids
Source: Stem Cell Reports. 2023 Jan 10;18(1):175–89. doi: 10.1016/j.stemcr.2022.12.002 (PMC9860116; doi:10.1016/j.stemcr.2022.12.002)
Supplement: Document S2. Article plus supplemental information [file mmc4.pdf]

Stem Cell Reports
Article

ISSCR
OPEN ACCESS

Comprehensive characterization of fetal and mature retinal cell identity to assess the fidelity of retinal organoids

Hani Jieun Kim,[1,2,3] Michelle O'Hara-Wright,[3,4] Daniel Kim,[1,3] To Ha Loi,[3,6] Benjamin Y. Lim,[4] Robyn V. Jamieson,[5,6] Anai Gonzalez-Cordero,[3,4,*] and Pengyi Yang[1,2,3,*]
[1]Computational Systems Biology Group, Children's Medical Research Institute, The University of Sydney, Westmead, NSW 2145, Australia
[2]School of Mathematics and Statistics, The University of Sydney, Sydney, NSW 2006, Australia
[3]School of Medical Sciences, Faculty of Medicine and Health, The University of Sydney, Camperdown, NSW 2006, Australia
[4]Stem Cell Medicine Group, Children's Medical Research Institute, The University of Sydney, Westmead, NSW 2145, Australia
[5]Specialty of Genomic Medicine, Faculty of Medicine and Health, University of Sydney, Westmead, NSW 2145, Australia
[6]Eye Genetics Research Unit, Children's Medical Research Institute, Sydney Children's Hospitals Network, Save Sight Institute, The University of Sydney, Westmead, NSW 2145, Australia
*Correspondence: agonzalez-cordero@cmri.org.au (A.G.-C.), pengyi.yang@sydney.edu.au (P.Y.)
https://doi.org/10.1016/j.stemcr.2022.12.002

SUMMARY

Characterizing cell identity in complex tissues such as the human retina is essential for studying its development and disease. While retinal organoids derived from pluripotent stem cells have been widely used to model development and disease of the human retina, there is a lack of studies that have systematically evaluated the molecular and cellular fidelity of the organoids derived from various culture protocols in recapitulating their *in vivo* counterpart. To this end, we performed an extensive meta-atlas characterization of cellular identities of the human eye, covering a wide range of developmental stages. The resulting map uncovered previously unknown biomarkers of major retinal cell types and those associated with cell-type-specific maturation. Using our retinal-cell-identity map from the fetal and adult tissues, we systematically assessed the fidelity of the retinal organoids in mimicking the human eye, enabling us to comprehensively benchmark the current protocols for retinal organoid generation.

INTRODUCTION

The human retina is a complex tissue and comprises various cell types that function together to convert light into biological signals. To understand the development and diseases of the human eye requires the characterization of molecular and cellular programs that define the identity of cells in the human retina. While access to the human retinal tissues is limited, human retinal organoids derived from pluripotent stem cells (PSCs) offer unprecedented opportunities to investigate early retinal development and therapeutic applications such as cell transplantation (Chahine Karam et al., 2022; Ribeiro et al., 2021; West et al., 2022). Early studies have used human embryonic stem cell (hESC)-derived embryoid bodies followed by two-dimensional (2D) culturing to generate retinal precursors, which were then isolated for directed and undirected differentiation into ganglion and amacrine cells and, to a lesser extent, photoreceptor precursor cells (Lamba et al., 2006). While the efficient production of the retinal progenitors under 2D conditions enabled useful initial applications in cell therapy studies (Lamba et al., 2006, 2010; Meyer et al., 2009; Osakada et al., 2008), these systems lack the capacity to recapitulate the three-dimensional (3D) features of the native retinal cells *in vivo*. This has led the field to develop advanced 3D *in vitro* structures that can recapitulate the physiological, morphological, and spatiotemporal patterns of the developing retina (Eiraku et al., 2011; Meyer et al., 2011; Nakano et al., 2012; Zhong et al., 2014).

The advances in the retinal organoid field have led to the development of state-of-the-art protocols that allow efficient and rapid formation of retinal organoids comprising all the retinal cell types: ganglion, amacrine, bipolar, horizontal, Müller glial, and photoreceptor (Afanasyeva et al., 2021). These organoids have been shown to generate mature features such as ribbon synapses (Artero Castro et al., 2019) and outer segments with physiological response to light stimuli (Wahlin et al., 2017; Zhong et al., 2014), showing remarkable functional similarity to the eye (Gonzalez-Cordero et al., 2017). A mixture of 2D and 3D protocols that do not require the addition of small molecules allows the generation of mature and light-sensitive photoreceptors with rudimentary outer segments (Zhong et al., 2014). Other protocols that involve a stepwise 2D-to-3D culture enable the formation of the embryoid body to be bypassed (Reichman et al., 2014). Other protocols have incorporated differentiation factors such as serum, retinoic acid (RA), taurine, and supplements N2 and B27 (Gonzalez-Cordero et al., 2017) and antioxidants and lipids (West et al., 2022) that have significantly improved the generation of photoreceptor outer segments. Most of these protocols share common medium components, such as BMP4 and IGF-1, but differ in their timing in the switch from 2D to 3D culture and/or the addition of certain molecules.

This is an open access article under the CC BY license (http://creativecommons.org/licenses/by/4.0/).

An increasing number of studies have begun profiling the human organoids derived from these protocols at single-cell resolution to investigate retinal development and disease (O'Hara-Wright and Gonzalez-Cordero, 2020). These studies have provided an unprecedented opportunity to investigate the heterogeneity of the retinal cell types and uncovered several new insights into retinal development, such as the discovery of a potentially novel regulator of cone fate (Kallman et al., 2020), a population of post-mitotic transitional cells (Sridhar et al., 2020), and the convergence of retinal organoid transcriptomes toward peripheral retinal cell types (Cowan et al., 2020). While these advancements have enhanced our understanding of retinal biology, in particular our understanding of the differences between retinal cell types between subdomains of human tissue (Voigt et al., 2021; Yan et al., 2020), the growing single-cell resource of the human retina and retinal organoids has yet been probed to systematically evaluate the state-of-the-art protocols for their capacity to produce organoids faithful to their *in vivo* counterpart.

Here, we performed an extensive curation of single-cell RNA-sequencing (scRNA-seq) datasets from human retinal tissue and organoids derived from a variety of differentiation protocols, generated a comprehensive map of retinal cellular identities of the mature and fetal eye, and benchmarked the fidelity of the human retinal organoid models in faithfully recapitulating the human eye. The extensive meta-atlas characterization of the retinal cellular identities enabled the discovery of an array of previously unknown marker genes of retinal cell types and those associated with cell-type-specific retinogenesis. Moreover, these cellular identities resolved by age were used to systematically benchmark the current protocols for their capacity to generate cell types that closely emulate their *in vivo* counterparts in terms of cell identity, cell-type proportion, and coverage. Finally, we developed a user-friendly application called Eikon (https://shiny.maths.usyd.edu.au/Eikon/) that helps users assess the fidelity of their retinal organoids.

RESULTS

Generating a cell-identity map of the human retina

Single-cell transcriptome profiling was applied to resolve the cellular identities in the retinal tissue (Figure 1A). To create a cell-identity map of the human retina, we began by compiling a collection of scRNA-seq datasets generated from the human retinal tissue in "mature" samples, including those from the postnatal stage and the adult retina (Figures 1B and S1A) (Cowan et al., 2020; Lu et al., 2020; Lukowski et al., 2019; Orozco et al., 2020; Yan et al., 2020).

We visualized the integrated datasets by cell type, dataset, and batch within each dataset using uniform manifold approximation projection (UMAP) (Figure 1C) and analyzed the number of cells in each cell type and their proportions in each dataset and batch (Figures 1D, S1B, and S1C). We observed that, while most of the major cell types are identified in the datasets and the proportions of cell types are largely consistent across batches within each dataset, the proportions of some cell types showed large variability across datasets (Figure S1C). Evaluating known retinal cell-type gene markers showed that their expression is highly cell-type specific (Figure 1E). To ensure the derivation of a high-quality reference, we performed re-annotation of all the cells in our retinal tissue meta-atlas using scReClassify, which is a semi-supervised learning method for assessing cell-type annotation accuracy in the original classification and for deriving classification accuracy probabilities (Kim et al., 2019). Keeping only the cells that had been correctly assigned their original annotations, we found that most cells in the reference were annotated with very high confidence (Figure S1D). To generate the gold standard reference for use in our downstream analyses, we included only the cells annotated with "very high" confidence (probabilities greater than 0.9, where 1 denotes the highest level of confidence). We show that, in most datasets (except Lukowski et al., 2019), greater than 90% of cells were assigned with high to very high confidence, enabling us to retain the majority of the cells for downstream analyses. Together, this large resource of scRNA-seq datasets profiling the human eye forms the basis for the characterization of the human retina cell types and for assessing the retinal cell identity and the fidelity of human retinal organoids in mimicking the *in vivo* identities.

Deriving robust cell-identity scores of genes for retina cell types

To resolve genes that underlie retinal cellular identity, we computed a cell-type-specific cell-identity score for each gene by dataset and batch using Cepo, a computational method for detecting cell-identity genes (Kim et al., 2021). The clustering of samples from across datasets and batches using Pearson's correlation of Cepo-derived gene statistics shows strong grouping by cell type irrespective of the origin of dataset and batch (Figure 2A).

To systematically quantify the influence of the total number of batches and the batch source on Cepo-derived cell-identity gene statistics, we conducted three assessments. First, we evaluated the stability of the Cepo statistics by randomly subsampling from 50% to 90% of all data batches for inclusion in the generation of the averaged Cepo scores. We then calculated Pearson's correlation between these scores against those generated from the entire batches. Our findings show that even with subsampling of up to 50% of the batches, the Cepo statistics remain highly reproducible and stable, showing only 0.01 loss in

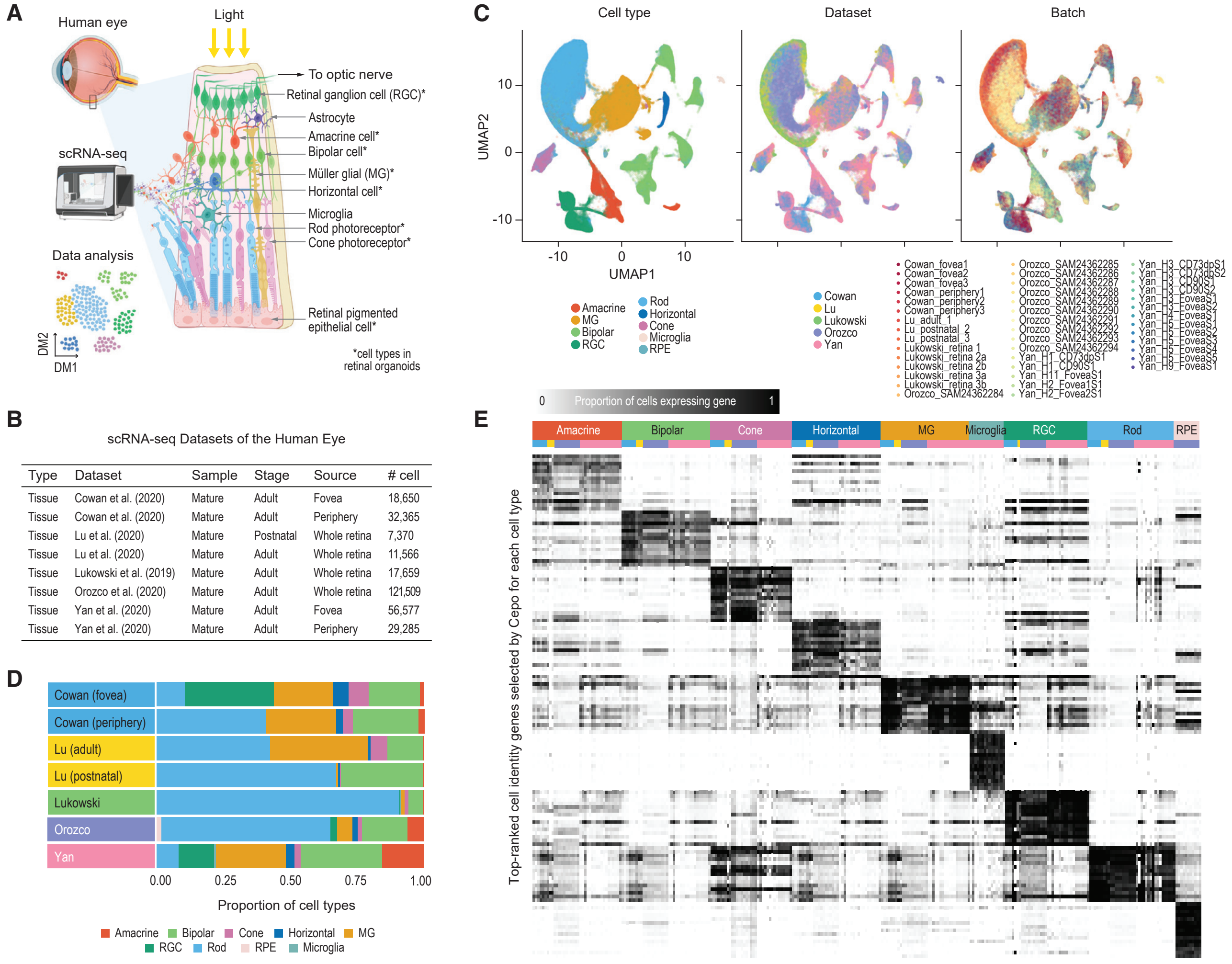

Type	Dataset	Sample	Stage	Source	# cell
Tissue	Cowan et al. (2020)	Mature	Adult	Fovea	18,650
Tissue	Cowan et al. (2020)	Mature	Adult	Periphery	32,365
Tissue	Lu et al. (2020)	Mature	Postnatal	Whole retina	7,370
Tissue	Lu et al. (2020)	Mature	Adult	Whole retina	11,566
Tissue	Lukowski et al. (2019)	Mature	Adult	Whole retina	17,659
Tissue	Orozco et al. (2020)	Mature	Adult	Whole retina	121,509
Tissue	Yan et al. (2020)	Mature	Adult	Fovea	56,577
Tissue	Yan et al. (2020)	Mature	Adult	Periphery	29,285

Figure 1. Curation of scRNA-seq datasets generated from the mature retinal tissue
(A) Schematic of single-cell transcriptomic profiling by scRNA-seq and the major cell types of the human retina.
(B) Summary of scRNA-seq datasets collected from the mature retinal tissues.
(C) UMAP representation of the transcriptomes of single cells. Cells are colored by their type (left), dataset of origin (middle), and batch in each dataset (right). Abbreviations: MG, Muller glial; RGC, retinal ganglion cell; RPE, retinal pigment epithelium.
(D) Proportions of cell types (color coded) and total number of cells in each batch and dataset.
(E) Expression patterns of known retinal cell-type marker genes across datasets and batches. As in (C), the color annotation denotes dataset and batch information.

correlation across the cell types (Figure S2A). Next, we applied different clustering methods on the samples and compared their concordance with respect to three sources of variation: cell type, dataset, or batch (see supplemental information). We found that the Cepo-derived cell-identity gene statistics enabled accurate clustering of samples by their cell type label, whereas both dataset and batch source had minimal influence on the clustering (Figure S2B). These findings were consistent across a varying number of genes (Figure S2B) and demonstrate the high reproducibility of the cell-type identity statistics calculated using Cepo for genes across our retinal resource.

Finally, we performed principal variance component analysis (Li et al., 2009) on all pairs of batches across datasets to quantify the degree of variance contributed by batch source (Figure 2B). Computing the degree of batch effect present in all pairs of batches generates a set of batch pairs ranging from those that exhibit low batch effect to those that exhibit high batch effect. As expected, batch pairs with both originating from the same dataset demonstrate lower batch effect, while those originating from different datasets demonstrate higher batch effect. We then computed the concordance of Cepo-derived gene statistics between the dataset pairs for each of the cell types and then evaluated these statistics against the

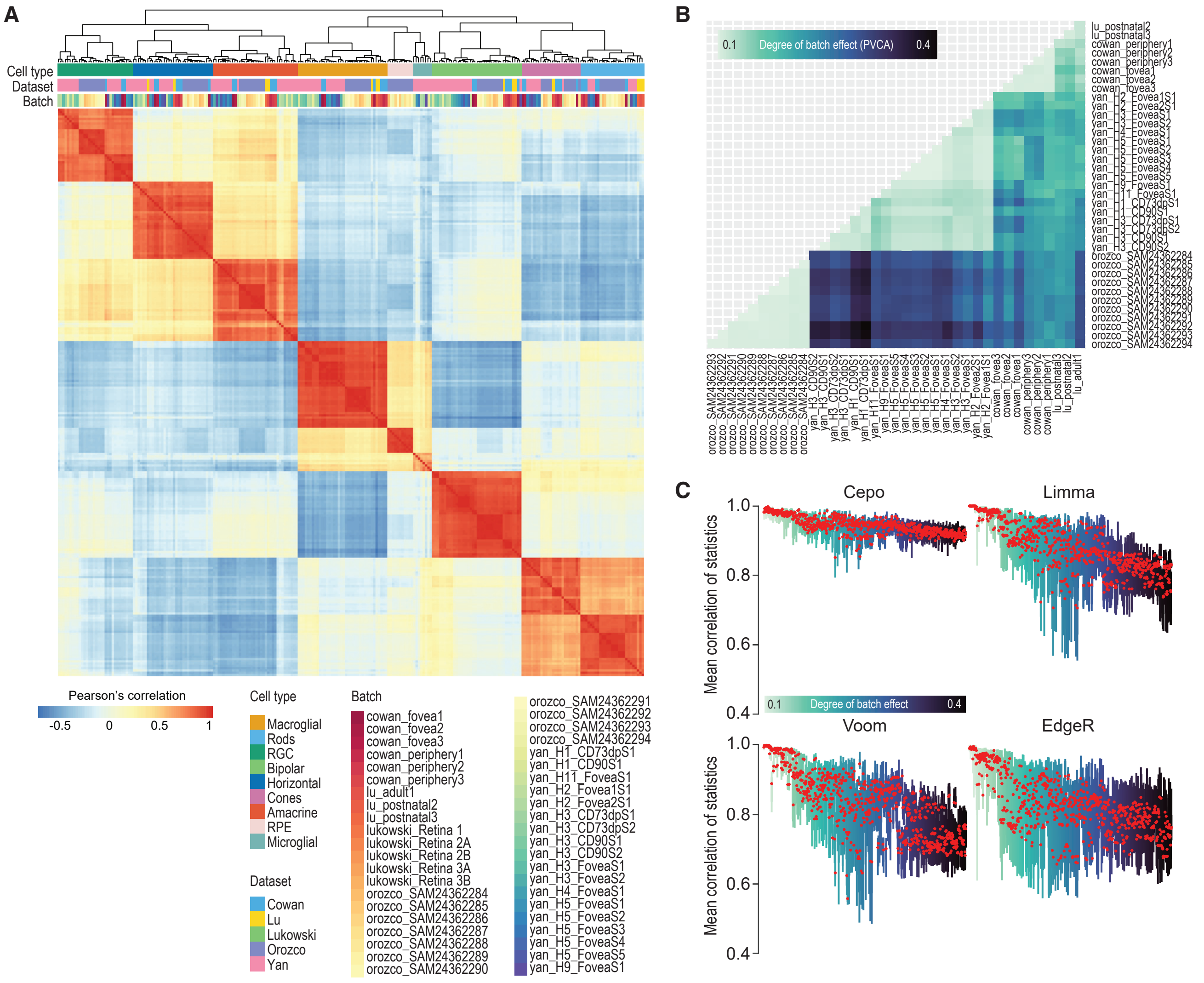

Figure 2. Comparative analysis of cell-type-specific gene statistics across datasets and batches

(A) Correlation heatmap of cell-identity gene statistics generated from Cepo (Kim et al., 2021) for each cell type across datasets and batches. The heatmap is hierarchically clustered by the similarity of correlation profiles.

(B) Pairwise assessment of batch effect using principal variance component analysis (PVCA). The proportion of variance contributed by batch in each pair of datasets is visualized. A darker color denotes stronger batch effect.

(C) Boxplots of mean correlation of gene statistics from all retinal cell types for each pair of datasets illustrated in (B) using Cepo, Limma, Voom, and EdgeR statistics.

degree of batch effect present in the data (Figures 2C and S2C). We found that the concordance in Cepo-derived gene statistics between the same cell types was largely retained across increasing batch effects (Figure S2C, first column). When comparing the robustness of other measures of cell-type-specific gene statistics (Limma, Voom, and EdgeR) against batch effect, we found that there was a gradual loss in concordance with increasing presence of batch effect across most cell types in these methods, with the most pronounced decrease in the statistics for the amacrine and bipolar cells (Figures 2 and S2C). Collectively, these results strongly support the high reproducibility of the Cepo-derived cell-identity gene statistics across the retina scRNA-seq datasets, highlighting their robustness against batch effect.

The retinal-cell-identity map uncovers novel cell-identity genes

To uncover potential new markers of cell types, we performed a systematic query search on PubMed on the genes highly ranked by Cepo (see supplemental information). We considered a gene as "known" if its query returned any

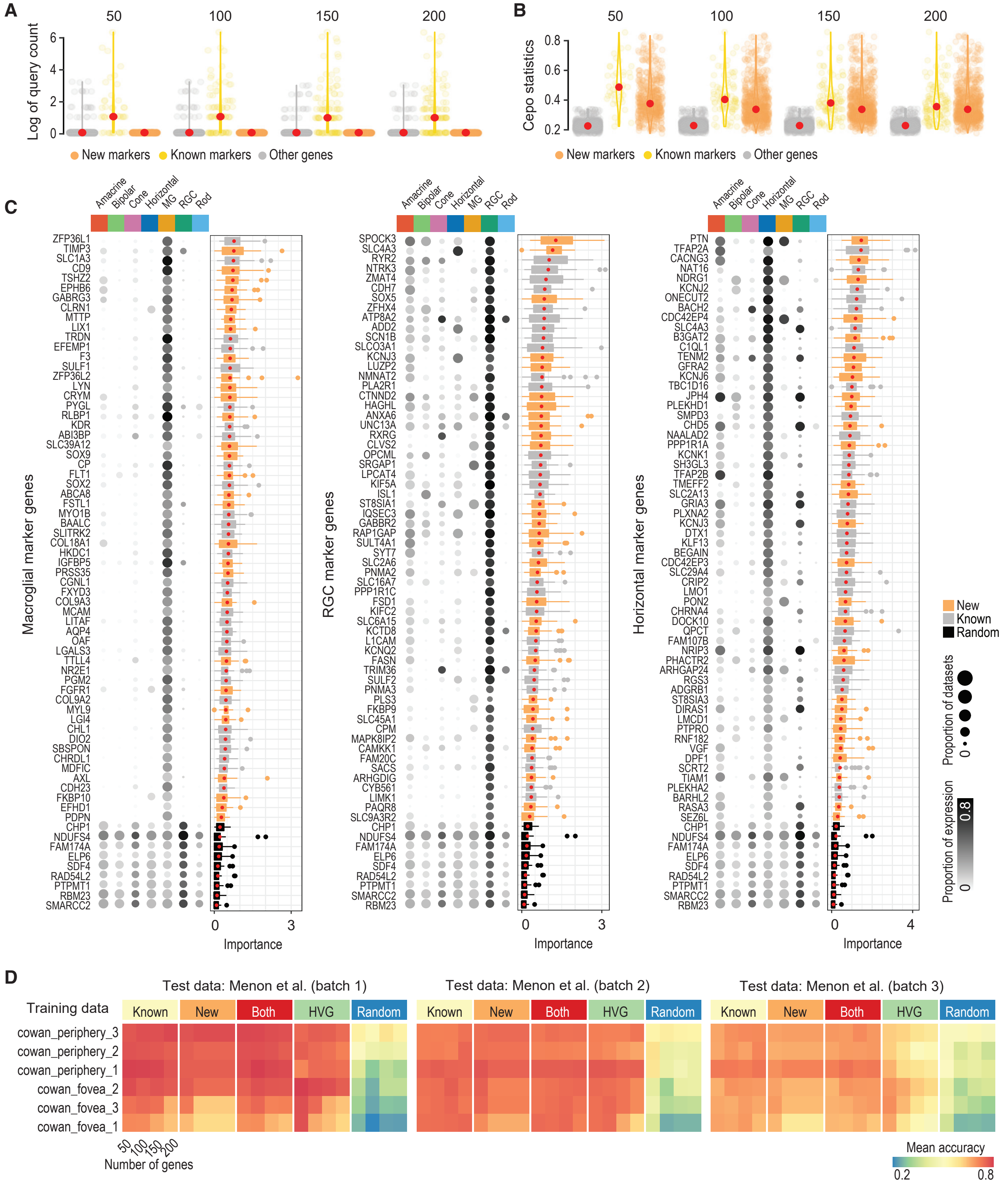

(legend on next page)

publication or as "novel" if its query returned no searches (Figure 3A). Genes found to be neither a known nor a novel gene were defined as "others." We found that, while many cell-identity genes uncovered by Cepo are known gene markers for their respective cell type, many genes that were not previously associated with the retinal cell types were ranked highly by their Cepo statistics (Figure 3B). Indeed, both known and new cell-type markers uncovered by Cepo showed expression specific to that cell type, whereas the randomly selected stable genes showed non-specific expression across all cell types, as demonstrated by the exclusive expression patterns of the known and new markers in Figures 3C and S3A. In agreement, known and new gene markers, but not the randomly selected stable genes, showed utility in classifying their respective cell types as indicated by their feature importance score computed from the random forest algorithm (Yang et al., 2021) (Figures 3A and S3B).

To further evaluate the utility of known and novel cell-identity genes in delineating the retinal cell types, we performed cell-type classification by training a *k*-nearest neighbor (*k*NN) classifier on different numbers of top genes ranked by Cepo and classifying an independent retina scRNA-seq dataset (Menon et al., 2019) (Figures 3D and S3B). We also assessed the classification accuracy using highly variable and randomly selected genes. We found that the known and new marker genes led to similar classification accuracy of cell types with an average >0.8. As expected, highly variable genes showed better than random classification accuracy but were much lower than Cepo-selected cell-identity genes. Last, the combination of known and new marker genes resulted in the best classification in most cases across all three batches of the data (Figure S3B). Taken together, these analyses support the discovery of new gene markers for each of the major retinal cell types (Table S1) and demonstrate their efficacy in delineating their respective cell types in the retina.

Identifying genes associated with human retina maturation

Several recent studies have profiled the developing human eye using scRNA-seq (Cao et al., 2020; Lu et al., 2020). To characterize these fetal samples and identify genes that are associated with maturation of each retina cell type (Figure 4A), we curated these datasets, each profiling a wide range of developmental stages (Figure S4A), and combined this resource with the mature retinal atlas. The UMAP revealed that the major cell types of the retina clustered together (Figures 4B and S4B). In line with the developmental birth order of retinal neurons and the Müller glial, we observed that fetal samples from up to approximately 100 days post-conception contained high proportions of retinal ganglion cells, horizontal cells, and cones (in relation to rod cells) (Figure S4C) (Shiau et al., 2021). Rods, amacrine, bipolar, and Müller cells demonstrated increased proportions after the 100-day time point, in agreement with the knowledge that these cell types are late-born cells. The final proportions of the fetal and mature cells in the combined retinal atlas demonstrate the inclusion of both age groups in all the cell types (Figure S4D).

To discover genes associated with human retinal maturation, we investigated the correlation of cell-type-specific Cepo gene statistics and the developmental age of the retinal samples. This analysis therefore enabled the generation of cell-type-specific scores that denote whether a gene exhibits a gain or a loss in cell-type-specific expression over time and the discovery of genes associated with maturation (Figure 4C), many of which have been established in the literature as associated with retinal development and disease (Azadi et al., 2010; Sapkota et al., 2014; Sinha et al., 2016; Zhu et al., 2002). Cepo statistics were computed as previously described for each cell type and batch, and the clustering results from their pairwise correlation relationships were visualized as a heatmap (Figure S4E). We found that the cell types were associated with varying numbers and proportions of maturation-related genes (Figures 4D and S4F) and that these maturation profiles are highly cell-type specific. These findings not only demonstrate the presence of cell-type-specific maturation programs but also highlight the capacity of our Cepo statistics to identify highly cell-type-specific genes (Figures 4E and 4F). Collectively, these findings reveal that substantive re-wiring occurs during development, whereby the re-wiring leads to distinct gene expression patterns between cell types.

Figure 3. Identification and validation of novel cell-type-specific gene markers of the retina
(A) Scatter violin plots of log of query count of the top 50, 100, 150, and 200 genes categorized into known or new genes. The scatterplot visualizes the PubMed queries for the results from all cell types and those from non-marker genes for comparison.
(B) Scatter violin plots of the same query results as in (A) but of the respective Cepo statistics.
(C) Cell-type-specific gene markers identified by Cepo. Proportion of cells expressing each marker in each cell type is represented by the gradient color and the proportion of datasets having each marker expressed is represented by the size of the balloons. Importance scores of gene markers are derived from random forest classification of cells using these markers. Novel markers are highlighted in orange and known markers are in gray. Randomly selected genes (in black) are included as controls.
(D) Classification accuracy of independent test data (Menon et al., 2019) from *k*NN classifiers trained on each of the Cowan datasets using known or new gene markers or their combination, highly variable genes (HVG), and randomly selected genes.

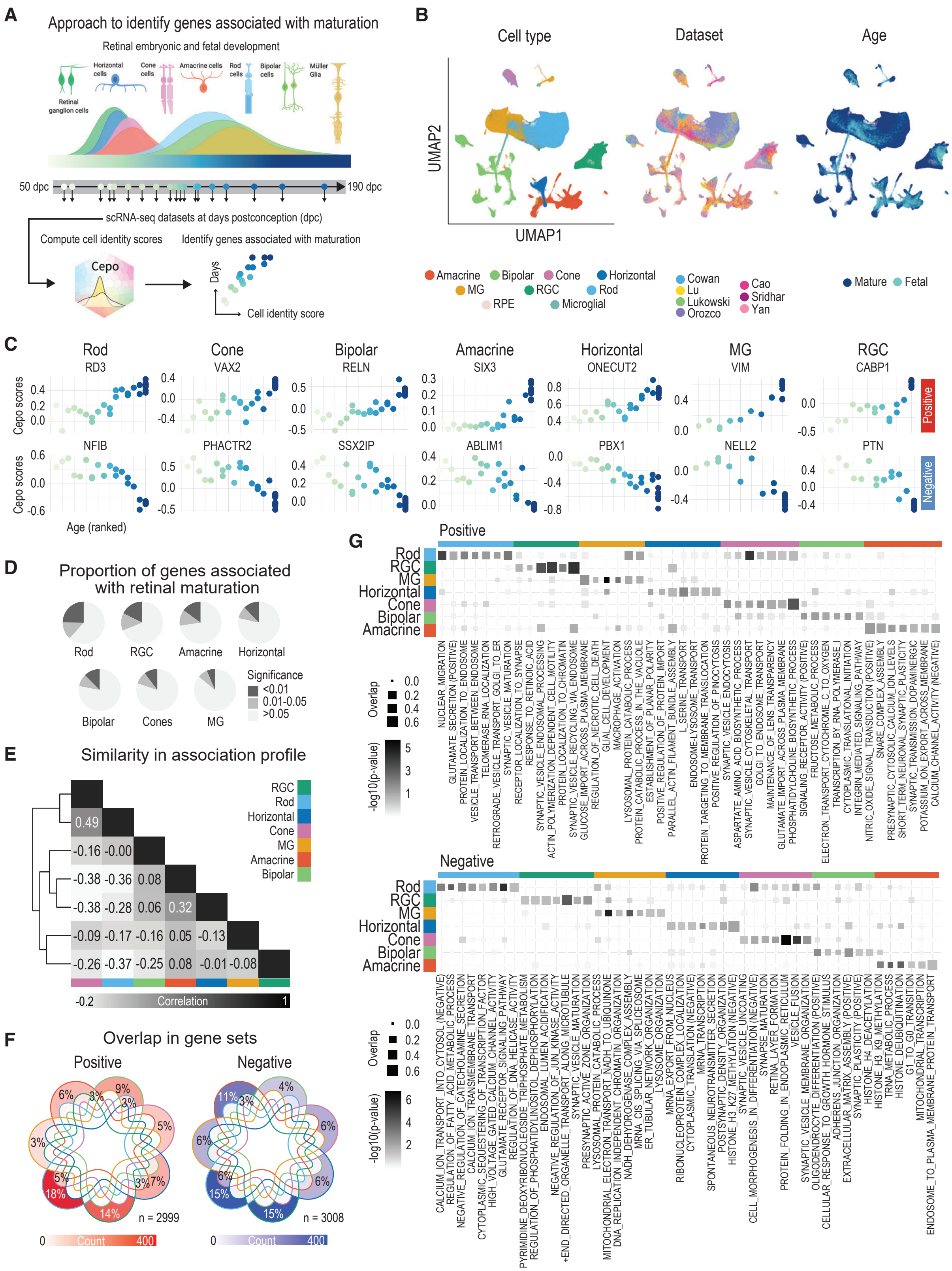

(legend on next page)

Next, we asked whether the genes associated with maturation of the major cell types of the retina represented those relevant to their respective cell types. To test this, we performed overrepresentation analyses using the gene sets shown in Figure S4F. Consistent with the neuronal identity of many of the retinal cell types, we observed that the most enriched pathways among the gene sets positively associated with retinal maturation were related to the formation and regulation of the synapses (Figure 4G). For example, consistent with the fact that amacrine cells are the dopaminergic neurons of the eye, a positive amacrine maturation profile was strongly enriched for the "regulation of synaptic transmission dopaminergic" pathway (Dacey, 1990).

Benchmarking framework to evaluate diverse retinal organoid differentiation protocols

Recent advances have led to the development of several state-of-the-art protocols for generating human retinal organoids that largely resembles the endogenous retina (Figure 5) (Afanasyeva et al., 2021; Berber et al., 2021; Llonch et al., 2018; Mellough et al., 2019; Völkner et al., 2016; Zhang et al., 2021). We curated public single-cell transcriptomics datasets obtained from these protocols (Cowan et al., 2020; Kallman et al., 2020; Lu et al., 2020; Sridhar et al., 2020), as well as generating our in-house data (Gonzalez-Cordero et al., 2017; West et al., 2022) (Figures 6A and S5A–S5D). While functional and molecular studies have evaluated the efficacy of these organoid protocols for efficient and robust generation of retinal cell types, no studies have systematically evaluated these protocols by comparing their global cellular and molecular profiles with human retinal tissue.

To address this gap, we devised a framework to systematically assess these retinal organoid protocols. In brief, leveraging the mature and fetal retinal atlas, we derived references denoting the cell-identity scores, maturation, the cell-type proportions, and the cell-type coverage aspired for the organoids. Importantly, we implemented six metrics based on the retinal atlas that measure the capacity of the protocols (1) to mimic the cellular identities of the mature and (2) the fetal retinal tissue; (3) to recapitulate the developmental stage; (4) to generate the cell-type proportions found in the tissue; (5) to generate all the major cell types in the retina; and finally (6) to generate the smallest amount of off-target cells (Figure 6B). The full description of how the references were generated and the metrics were computed can be found in the supplemental information.

Benchmarking the fidelity of human retinal organoids to the human tissue

Using this benchmarking framework, we evaluated the capacity of each protocol to generate cell types that closely mimic the human eye. We first evaluated the similarity between the protocols by comparing the cell-type identity scores generated for each dataset and batch. We found that our in-house-generated retinal organoids (Gonzalez-Cordero et al., 2017; West et al., 2022) and those of Cowan et al. (2020) demonstrated the highest degree of similarity and also the highest level of consistency between batches (Figure 6C). Next, for each of the datasets we computed the cell-identity metric to assess the fidelity of each cell type of the organoids against the mature and fetal references. Ordering the samples by their similarity to the reference revealed that the organoids derived from our in-house protocol closely followed by the Cowan protocol, consistently outperforming those at similar ages derived from other protocols in terms of achieving a high fidelity in cellular identity against both references (Figure 6D). Immunohistochemistry analysis indeed confirmed the presence of all the major retinal cell types in day 210 human retinal organoids (Figure 6E, n = 15 organoids; N = 3 differentiation batches; generated using West et al., 2022).

To perform a cross-laboratory and cross-protocol benchmarking of the human retinal organoids, we applied the six fidelity metrics to the organoid datasets. The cell-identity metric was computed as the average of the cell-type-specific scores described in Figure 6D. Among organoids grown for 100 days or more, the overall score showed that our protocols (Gonzalez-Cordero et al., 2017; West

Figure 4. The retinal cell-identity scores uncover genes associated with retinal maturation

(A) Schematic workflow to derive cell-type-specific maturation-associated genes. Scaling by day post-conception (dpc).

(B) UMAP of cell transcriptome profiles combining both adult and fetal tissues. Cells are colored by their type (left), dataset (middle), and sample type (right).

(C) Scatterplots of developmental age (x axis) and Cepo statistics (y axis). Points denote individual samples and are colored by their ranked developmental time point. The top and bottom show genes that are positively and negatively associated with age, respectively.

(D) Proportions of highly significant, significant, and insignificant maturation-associated genes for each cell type. Highly significant, false discovery rate [FDR]-adjusted $p < 0.01$; significant, FDR-adjusted p between 0.01 and 0.05; and insignificant, FDR-adjusted $p > 0.05$.

(E) Similarity in maturation association profiles between the cell types in terms of Pearson's correlation coefficient.

(F) Overlap among the positively (left) and negatively (right) significant genes (FDR-adjusted $p < 0.05$). The total percentage of overlap is highlighted for intersections greater than 2% of overlaps. The color scale denotes the absolute number of genes in each gene set.

(G) Enrichment of gene sets positively and negatively correlated with age.

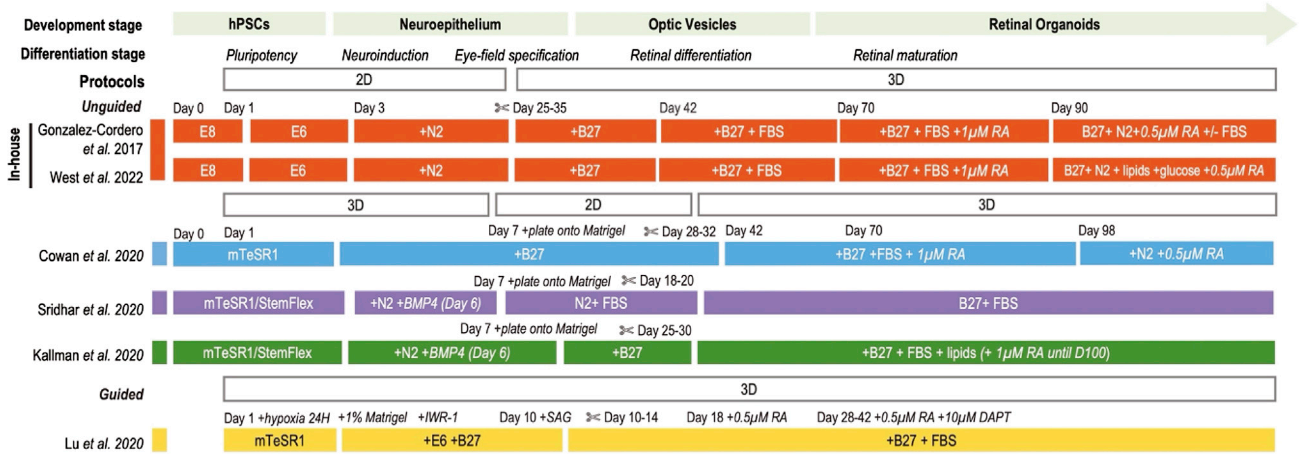

Figure 5. Protocols of human retinal organoids

Overview of the culture systems for generating human retinal organoids. The schematic illustrates the timeline of retinal induction, differentiation, and maturation steps, outlining 2D and 3D stages, key supplements, and factors added to culture conditions across the published protocols. Abbreviations: hPSCs, human pluripotent stem cells; E8, Essential 8; E6, Essential 6; RA, retinoic acid; H, hour; DAPT, N-[N-(3,5-difluorophenacetyl)-*l*-alanyl]-S-phenyl glycine t-butyl ester) γ-secretase inhibitor; ✂, dissection/dislodgment.

et al., 2022) generated retinal cell types most faithful to both mature and fetal tissue, closely followed by those from the Cowan protocol. The organoids from the Kallman and Sridhar protocols at similar culture periods (grown until 170 and 205 days, respectively) performed less in terms of the cell-identity metrics. While it is not appropriate to directly compare the organoids from the early and late culture stages, it is worth noting that these organoids show minimal resemblance to the fetal reference, suggesting either that progenitor cells present at these early time points of organoid are not captured in our fetal reference due to difference in age or that these organoids do not robustly recapitulate the fetal retinal cell types.

Next, the coverage metric was computed as the proportion of the major cell types of the retina present in the organoid data. We observed that our protocols achieved the highest coverage of the major cell types of the retina consistently across five batches that differed in organoid cell line, organoid batch, and in-house protocols (West et al., 2022; Gonzalez-Cordero et al., 2017, ±FBS) (Figure 6F). This includes retinal ganglion cells (RGCs), which are known to decrease in numbers with extended organoid culturing. We observed RGCs in our cultures, albeit in small numbers, making a small proportion of the total cells (Figure S5B). This was confirmed by the presence of THY1 (CD90)- and NeuN-positive cells in the 210-day-old organoids (Figures 6E and S6E). Furthermore, gene expression of *THY1* confirmed the specificity of this marker to RGCs (Figure S6F).

To investigate the developmental relevancy of the organoids, the maturation metric computed from all the individual cell-identity scores from the mature and fetal data was used. Briefly, we assessed the capacity of the organoids to either positively (more adult-like) or negatively (more fetal-like) correlate with the time-resolved cell-identity profiles as a proxy for their developmental relevancy (Figure S6A). Our findings show that young organoids (≤ 60 days) demonstrate a strong negative correlation against the age-ranked references, whereas organoids older than 170 days showed a strong correlation against the age-ranked references. Thus, the overall scores, which have been averaged for each cell type, show either a strong adult- or a strong fetal-like profile of organoids at the bookends of the protocols (Figure 6F).

The metric—the proportion of cell types—was computed from the mature and fetal reference, the latter of which is also subdivided into two references denoting early and late fetal maturation. We found that, again, our protocols excelled among the protocols in generating cell-type proportions that represent those found in the mature human eye and not the fetal eye (Figure 6F). Intriguingly, even though the early-stage organoids poorly recapitulated the cellular identities of the fetal tissue, we found that their cell-type proportions better reflected those of the fetal eye (Figure 6F).

The final metric, the proportion of off-targets, was computed for all the samples using scClassify, a multi-scale classification algorithm that identifies intermediate and unassigned cells (Lin et al., 2020). The average proportion of cells classified into either of these categories was used as the proxy for the generation of off-target cells, which is important to minimize when generating cells for transplantation. We show that, as expected, the mature organoids generated fewer off-target cells (<10%) than

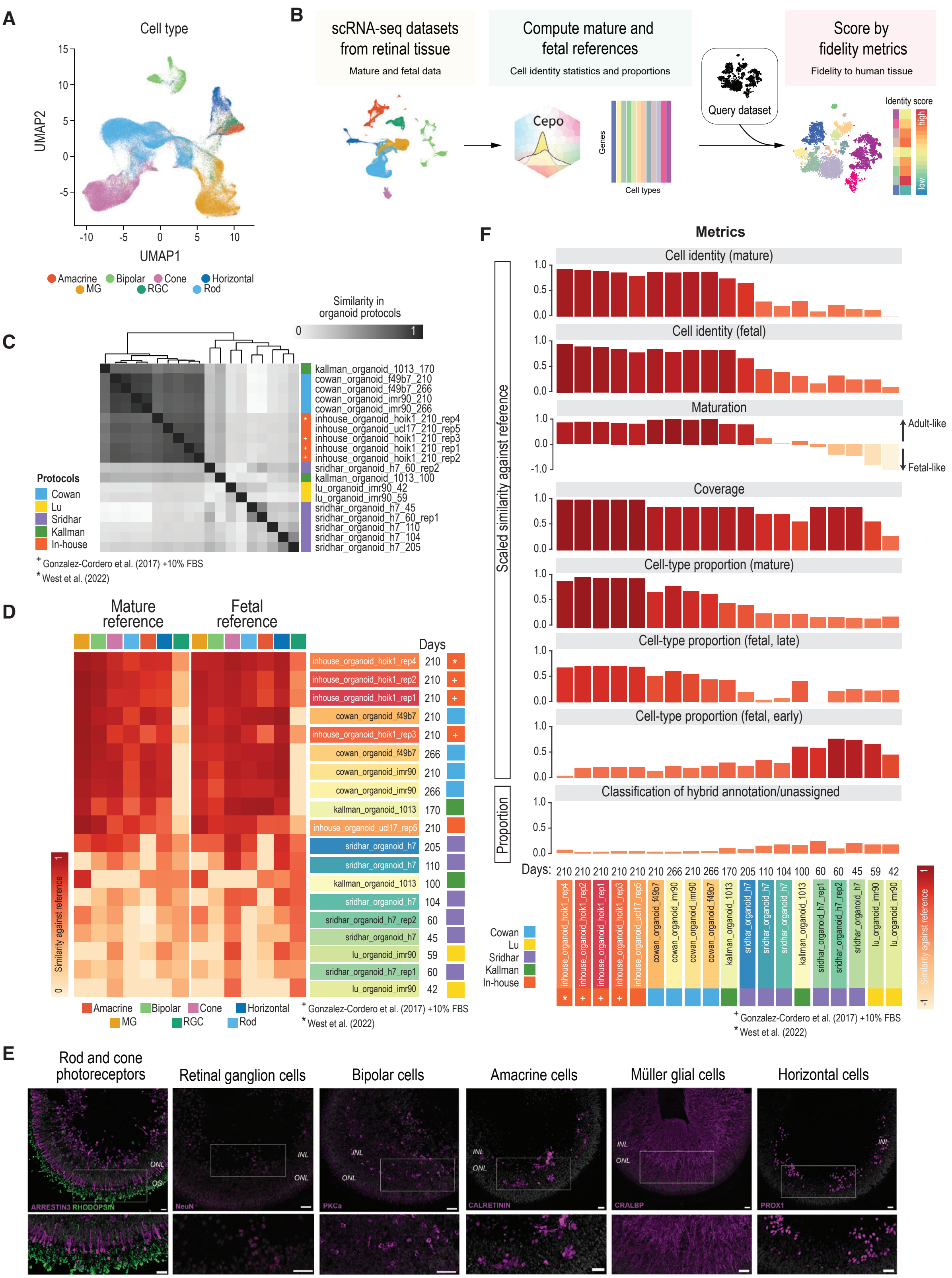

(legend on next page)

younger organoids, which may consist of cells undergoing development (Figure S6B), and that most protocols demonstrated a high capacity to generate on-target cells (Figure 6F).

Overall, our benchmarking study revealed that our in-house protocols (Gonzalez-Cordero et al., 2017; West et al., 2022) generate cell types of the retina with the highest fidelity to the human adult retina in terms of their cellular identity, cell-type proportion, and coverage (Figures 6F and S6C). To facilitate future benchmarking of PSC-derived human retinal organoids generated from various differentiation protocols, we have implemented our retinal cell-type identity map and the benchmarking framework as an online resource (https://shiny.maths.usyd.edu.au/Eikon/) where the fidelity and quality of organoids can be explored, assessed, and scored.

DISCUSSION

A key contribution of this study is the discovery of potential novel markers of the major retinal cell types and of retinogenesis. To elucidate this array of cell-type-specific markers, we integratively analyzed the scRNA-seq datasets of the fetal and the mature retina. This is now possible with the advent of single-cell technologies, which has enabled the extensive exploration of retinogenesis at a single-cell resolution. For example, recent studies have begun elucidating the trajectories of retinal-derived photoreceptor differentiation, highlighting markers that distinguish rod versus cone specification during development (Kallman et al., 2020). Our study takes advantage of the rich resource of scRNA-seq datasets of the developing and mature retina to interrogate genes that are associated with retinogenesis. We uncovered intrinsic differences in the maturation profile between all the major cell types of the retina and showed that these specification profiles were largely cell-type specific. While we anticipate that these genes can be used as a resource for further studies to investigate retinogenesis in development and disease, we note that future studies are required to validate these findings in *in vivo* and *in vitro* systems.

Another contribution is our effort to benchmark human retinal organoids generated by diverse differentiation protocols against the *in vivo* human retina. This has revealed that unguided human retinal organoids generated using our in-house differentiation protocol and that of Cowan et al. were top performers (Cowan et al., 2020; Gonzalez-Cordero et al., 2017; West et al., 2022). Notably, these two protocols have similar time courses of RA addition and supplementation. All differentiation protocols showed efficient generation of organoids; however, our 2D-3D is able to generate organoids from PSC confluence in a simpler workflow (Figure 5) (Gonzalez-Cordero et al., 2017; Reichman et al., 2014; West et al., 2022). Furthermore, we have recently shown that optimization of late-stage culture conditions with lipid supplementation enhances maturation of photoreceptor cells (West et al., 2022). Among the benchmarked protocols, our protocol generates cell types of the retina with the highest fidelity to the human retinal tissue across various evaluation metrics (Figures 6D–6F). These culture improvements possibly explain the presence of a small number of RGCs in the mature in-house organoids only.

While our benchmarking study highlighted that some of the protocols can successfully develop retinal organoids closely resembling many aspects of the human retina, it also highlighted remaining challenges. We observe that the organoids, in particular those collected at earlier maturation stages (50–170 days), do not fully recapitulate the developmental stages of the human retina (Figures 6D and 6F): a feature to be taken into consideration when investigating human eye development and retinogenesis using human retinal organoids. Furthermore, none of the protocols can currently generate retinal pigment epithelium (RPEs), which

Figure 6. Benchmarking the human retinal organoid protocols in terms of their fidelity to the retinal tissue

(A) UMAP of cell transcriptome profiles from human retinal organoids.

(B) Schematic of the benchmarking procedure and evaluation metrics.

(C) Similarity in cell-identity profiles between the scRNA-seq datasets generated from various organoid protocols. Similarity is measured in terms of Pearson's correlation coefficient.

(D) Benchmarking results of the fidelity of the individual cell types to either the mature or the fetal reference. The cell-identity metric gives a scaled score between 0 and 1, where 1 denotes a high capacity and 1 denotes a low capacity of the protocol to generate cell types that closely match their tissue counterparts.

(E) Representative images of retinal cell types in human retinal organoids at 210 days of age. Abbreviations: ONL, outer nuclear layer; INL, inner nuclear layer; OS, outer segments. Scale bars: 20 μm.

(F) Final benchmarking results of the human retinal organoid protocols ranked by the combined score from six evaluation metrics: cell identity (mature), cell identity (fetal), maturation, coverage, cell-type proportion (mature), and proportion of potential off-target cells. The scores, except the maturation and proportion off-targets, have been scaled from 0 to 1 within each evaluation metric, where a value of 1 denotes a high capacity of the protocol to mimic the reference and 0 denotes low capacity.

is a clear shortcoming of the protocols. Future benchmarking efforts will require the incorporation of studies that generate single-cell transcriptomic profiles of RPEs across development (Hu et al., 2019; Petrus-Reurer et al., 2022).

One of the key limitations of the study is that the fidelity metrics do not address important features of the retina, such as the profiles of the other omics layers, spatial patterning, retinal organoid functionality, and domain-specific characteristics. Recently, a few studies have begun profiling the retinal organoid using multi-modal single-cell technologies (Thomas et al., 2022; Wahle et al., 2022). The study by Thomas et al. mapped the *cis*-regulatory elements of the developing and mature human retina and showed that human retinal organoids are capable of emulating the DNA accessibility of the human retina (Thomas et al., 2022). The spatial organization of the retina is an important component to evaluate for tissues, like the retina, that have highly complex, ordered, and specialized structures. Toward this end, spatial transcriptomics that can profile the transcriptomes while retaining the spatial coordinates of the single cells would enable us to assess the capacity of protocols to generate organoids that are spatially organized to closely emulate that of *in vivo* tissue (Rao et al., 2021). Therefore, future studies will be required to develop computational methods that can quantify the reference spatial organization for each cell type of the retina and measure the fidelity of organoids to conform to the reference.

EXPERIMENTAL PROCEDURES

Resource availability

Corresponding author

Further information and requests for recourses and reagents should be directed to and will be fulfilled by the corresponding authors, Anai Gonzalez-Cordero (agonzalez-cordero@cmri.org.au) and Pengyi Yang (pengyi.yang@sydney.edu.au).

Materials availability

This study did not generate new unique reagents.

Data and code availability

The sequencing data generated in this study have been deposited in the Gene Expression Omnibus (GEO) under accession no. GSE201356. The code generated during this study is available upon reasonable request to one of the corresponding authors. Eikon (https://shiny.maths.usyd.edu.au/Eikon/) is available as an interactive web application to explore the fidelity of retinal organoids.

Cell culture and retinal organoid generation

Human induced pluripotent stem cells

HPSI0314i-hoik_1 (RRID:CVCL_AE82) was obtained from ECCAC. UCLOOi017-A-1 was derived from healthy donor peripheral blood mononuclear cells (PBMCs) as described previously (Fernando et al., 2022). PBMCs were isolated using density gradient centrifugation. Briefly, 25 mL of whole blood diluted 1:1 with PBS was layered on top of 15 mL of Ficoll-Paque Premium and centrifuged with brake and accelerator off at 500*g* for 30 min, and the cloudy interphase containing PBMCs was collected. Two million cells were cultured for 6 days in hematopoietic expansion medium StemSpan H3000, with the addition of EPO, IL-3, dexamethasone, ascorbic acid, SCF, and IGF-1. Following expansion, 200,000 cells were nucleofected using an Amaxa 4D nucleofector with Addgene plasmids. The nucleofected cells were plated in a well of a six-well plate coated with Geltrex matrix and transitioned to Essential 8 medium.

Human induced pluripotent stem cell maintenance and differentiation

Cells were incubated at 37°C in 5% CO_2. Human induced PSCs (hiPSCs) were grown and expanded under feeder-free conditions using Essential 8 medium (E8; Life Technologies) on six-well plates coated with Geltrex (Invitrogen) at a concentration of 1:100. The medium was replaced daily and cells were passaged at 70% confluency via 5–10 min of 37°C incubation with Versene solution (0.48 mM) (Life Technologies) to detach clumps of cells. Cell clumps were resuspended at a ratio of 1:6–1:12 in E8 with 10 μM ROCK inhibitor (Y-27632 dihydrochloride; Tocris) and seeded in fresh Geltrex-coated six-well plates. For differentiation, hiPSCs were grown to 90%–100% confluency.

Generation of retinal organoids from hiPSCs

Retinal organoids were differentiated as previously described (Gonzalez-Cordero et al., 2017; West et al., 2022) with some modifications. Briefly, at 90%–100% confluency (denotated day 1), hiPSC medium was replaced with Essential 6 (E6; Life Technologies) daily for 2 consecutive days. On day 3, E6 medium was replaced with pro-neural induction medium (PIM; Advanced DMEM/F12, 1× N2 supplement, 1.9 mM L-glutamine, 1× MEM-NEAA, 10% antibiotic-antimycotic [all Life Technologies]). Optic vesicles displaying neuroretinal epithelium were manually isolated using a needle under an EVOS XL microscope (Invitrogen) between days 25 and 35 and transitioned to 3D suspension culture in low-binding 96-well U-shaped plates and retinal differentiation medium (RDM; DMEM high glucose 68% v/v, Ham's F-12 nutrient mix with GlutaMAX supplement 29% v/v, 1× B-27 supplement minus vitamin A, 10% antibiotic-antimycotic [All Life Technologies]). At day 42, RDM was replaced with RDM + factors (RDMF; DMEM high glucose 60% v/v, Ham's F-12 nutrient mix with GlutaMAX supplement 26% v/v, 2× GlutaMAX supplement, 1× B-27 supplement minus vitamin A, 10% antibiotic-antimycotic) [all Life Technologies], FBS 10% v/v [Bovogen]). At day 70, retinal organoids were transferred into low-binding 24-well plates and the medium was replaced with ALT70 (Advanced DMEM/F-12 85% v/v, 10% FBS, 2× GlutaMAX supplement, 1× B-27 supplement minus vitamin A, 10% antibiotic-antimycotic [all Life Technologies], 100 μM taurine [Sigma Aldrich]) and supplemented with 1 μM all-*trans*-RA to enhance photoreceptor development. At day 90 and until the experimental endpoint, the medium was replaced with ALT90 (Advanced DMEM/F-12, 2× GlutaMAX supplement, 1× B-27 supplement minus vitamin A, 1× N2 supplement, 7 mM glucose, 10% antibiotic-antimycotic, 1× lipid mixture [all Life Technologies], 100 μM taurine [Sigma Aldrich], ±10% FBS [Bovogen]) and supplemented with 0.5 μM RA. The medium was replaced Monday, Wednesday, and Friday and the were cells maintained at 37°C in 5% CO_2.

Immunohistochemistry

Retinal organoids were washed with PBS, fixed for 40–60 min in 4% paraformaldehyde prior to incubation in 20% sucrose. After 210 days in culture, organoids were embedded in OCT, frozen in liquid nitrogen, and then cryosectioned at 14 μm thickness. Cryosections were blocked in 5% serum in blocking solution (1% bovine serum albumin in PBS with 0.1% Triton X) for 2 h. Primary antibody (Table S2) diluted in the blocking solution was incubated overnight at 4°C. Sections were washed with PBS and incubated with secondary antibody (Alexa Fluor 488, 546 secondary antibodies) at room temperature for 2 h. Sections were counterstained with DAPI.

SUPPLEMENTAL INFORMATION

Supplemental information can be found online at https://doi.org/10.1016/j.stemcr.2022.12.002.

AUTHOR CONTRIBUTIONS

Conceptualization, H.J.K., A.G-C., and P.Y.; methodology, H.J.K., M.O-W., A.G-C., and P.Y.; investigation, H.J.K., D.K., A.G-C., and P.Y.; data generation, M.O-W., T.H.L., B.Y.L., R.V.J., A.G-C., and P.Y.; writing – original draft, H.J.K., M.O-W., A.G-C., and P.Y.; writing – review & editing, all authors; supervision, R.V.J., A.G-C., and P.Y.; funding acquisition, R.V.J., A.G-C., and P.Y.

ACKNOWLEDGMENTS

We are grateful for the intellectual feedback from the Embryology Unit at Children's Medical Research Institute (CMRI) and engagement of the colleagues at the School of Mathematics and Statistics, The University of Sydney, and Sydney Precision Bioinformatics Alliance. We acknowledge the CMRI Single Cell Analytics, Advanced Microscopy Centre and the Australian Cancer Research Foundation Telomere Analysis Centre for supporting this work. This work is funded by a National Health and Medical Research Council (NHMRC) Investigator Grant (1173469) to P.Y., the Ophthalmic Research Institute of Australia, and the Luminesce Alliance (PPM1 K5116/RD274). The Luminesce Alliance is a not-for-profit joint venture between CMRI, the Sydney Children's Hospitals Network, and the Children's Cancer Institute and is affiliated with the University of Sydney and the University of NSW. The graphical abstract was generated using BioRender (https://biorender.com).

CONFLICTS OF INTEREST

The authors declare no competing interests.

Received: April 29, 2022
Revised: December 7, 2022
Accepted: December 7, 2022
Published: January 10, 2023

REFERENCES

Afanasyeva, T.A.V., Corral-Serrano, J.C., Garanto, A., Roepman, R., Cheetham, M.E., and Collin, R.W.J. (2021). A look into retinal organoids: methods, analytical techniques, and applications. Cell. Mol. Life Sci. *78*, 6505–6532.

Artero Castro, A., Rodríguez Jimenez, F.J., Jendelova, P., and Erceg, S. (2019). Deciphering retinal diseases through the generation of three dimensional stem cell-derived organoids: concise Review. Stem Cells *37*, 1496–1504.

Azadi, S., Molday, L.L., and Molday, R.S. (2010). RD3, the protein associated with Leber congenital amaurosis type 12, is required for guanylate cyclase trafficking in photoreceptor cells. Proc. Natl. Acad. Sci. USA *107*, 21158–21163.

Berber, P., Milenkovic, A., Michaelis, L., and Weber, B.H.F. (2021). Retinal organoid differentiation methods determine organoid cellular composition. J. Transl. Genet. Genom. *5*, 292–303.

Cao, J., O'Day, D.R., Pliner, H.A., Kingsley, P.D., Deng, M., Daza, R.M., Zager, M.A., Aldinger, K.A., Blecher-Gonen, R., Zhang, F., et al. (2020). A human cell atlas of fetal gene expression. Science *370*. eaba7721.

Chahine Karam, F., Loi, T.H., Ma, A., Nash, B.M., Grigg, J.R., Parekh, D., Riley, L.G., Farnsworth, E., Bennetts, B., Gonzalez-Cordero, A., et al. (2022). Human iPSC-derived retinal organoids and retinal pigment epithelium for novel intronic RPGR variant assessment for therapy suitability. J. Pers. Med. *12*, 502.

Cowan, C.S., Renner, M., De Gennaro, M., Gross-Scherf, B., Goldblum, D., Hou, Y., Munz, M., Rodrigues, T.M., Krol, J., Szikra, T., et al. (2020). Cell types of the human retina and its organoids at single-cell resolution. Cell *182*, 1623–1640.e34.

Dacey, D.M. (1990). The dopaminergic amacrine cell. J. Comp. Neurol. *301*, 461–489.

Eiraku, M., Takata, N., Ishibashi, H., Kawada, M., Sakakura, E., Okuda, S., Sekiguchi, K., Adachi, T., and Sasai, Y. (2011). Self-organizing optic-cup morphogenesis in three-dimensional culture. Nature *472*, 51–56.

Fernando, M., Lee, S., Wark, J.R., Xiao, D., Lim, B.Y., O'Hara-Wright, M., Kim, H.J., Smith, G.C., Wong, T., Teber, E.T., et al. (2022). Differentiation of brain and retinal organoids from confluent cultures of pluripotent stem cells connected by nerve-like axonal projections of optic origin. Stem Cell Rep. *17*, 1476–1492.

Gonzalez-Cordero, A., Kruczek, K., Naeem, A., Fernando, M., Kloc, M., Ribeiro, J., Goh, D., Duran, Y., Blackford, S.J.I., Abelleira-Hervas, L., et al. (2017). Recapitulation of human retinal development from human pluripotent stem cells generates transplantable populations of cone photoreceptors. Stem Cell Rep. *9*, 820–837.

Hu, Y., Wang, X., Hu, B., Mao, Y., Chen, Y., Yan, L., Yong, J., Dong, J., Wei, Y., Wang, W., et al. (2019). Dissecting the transcriptome landscape of the human fetal neural retina and retinal pigment epithelium by single-cell RNA-seq analysis. PLoS Biol. *17*. e3000365.

Kallman, A., Capowski, E.E., Wang, J., Kaushik, A.M., Jansen, A.D., Edwards, K.L., Chen, L., Berlinicke, C.A., Joseph Phillips, M., Pierce, E.A., et al. (2020). Investigating cone photoreceptor development using patient-derived NRL null retinal organoids. Commun. Biol. *3*, 82.

Kim, H.J., Wang, K., Chen, C., Lin, Y., Tam, P.P.L., Lin, D.M., Yang, J.Y.H., and Yang, P. (2021). Uncovering cell identity through differential stability with Cepo. Nat. Comput. Sci. *1*, 784–790.

Kim, T., Lo, K., Geddes, T.A., Kim, H.J., Yang, J.Y.H., and Yang, P. (2019). scReClassify: post hoc cell type classification of single-cell rNA-seq data. BMC Genom. *20*, 913.

Lamba, D.A., Karl, M.O., Ware, C.B., and Reh, T.A. (2006). Efficient generation of retinal progenitor cells from human embryonic stem cells. Proc. Natl. Acad. Sci. USA *103*, 12769–12774.

Lamba, D.A., McUsic, A., Hirata, R.K., Wang, P.-R., Russell, D., and Reh, T.A. (2010). Generation, purification and transplantation of photoreceptors derived from human induced pluripotent stem cells. PLoS One *5*. e8763.

Li, J., Bushel, P.R., Chu, T.-M., and Wolfinger, R.D. (2009). Principal variance components analysis: estimating batch effects in microarray gene expression data. In Batch Effects and Noise in Microarray Experiments (John Wiley & Sons, Ltd), pp. 141–154.

Lin, Y., Cao, Y., Kim, H.J., Salim, A., Speed, T.P., Lin, D.M., Yang, P., and Yang, J.Y.H. (2020). scClassify: sample size estimation and multiscale classification of cells using single and multiple reference. Mol. Syst. Biol. *16*. e9389.

Llonch, S., Carido, M., and Ader, M. (2018). Organoid technology for retinal repair. Dev. Biol. *433*, 132–143.

Lu, Y., Shiau, F., Yi, W., Lu, S., Wu, Q., Pearson, J.D., Kallman, A., Zhong, S., Hoang, T., Zuo, Z., et al. (2020). Single-cell analysis of human retina identifies evolutionarily conserved and species-specific mechanisms controlling development. Dev. Cell *53*, 473–491.e9.

Lukowski, S.W., Lo, C.Y., Sharov, A.A., Nguyen, Q., Fang, L., Hung, S.S., Zhu, L., Zhang, T., Grünert, U., Nguyen, T., et al. (2019). A single-cell transcriptome atlas of the adult human retina. EMBO J. *38*. e100811.

Mellough, C.B., Collin, J., Queen, R., Hilgen, G., Dorgau, B., Zerti, D., Felemban, M., White, K., Sernagor, E., and Lako, M. (2019). Systematic comparison of retinal organoid differentiation from human pluripotent stem cells reveals stage specific, cell line, and methodological differences. Stem Cells Transl. Med. *8*, 694–706.

Menon, M., Mohammadi, S., Davila-Velderrain, J., Goods, B.A., Cadwell, T.D., Xing, Y., Stemmer-Rachamimov, A., Shalek, A.K., Love, J.C., Kellis, M., and Hafler, B.P. (2019). Single-cell transcriptomic atlas of the human retina identifies cell types associated with age-related macular degeneration. Nat. Commun. *10*, 4902.

Meyer, J.S., Shearer, R.L., Capowski, E.E., Wright, L.S., Wallace, K.A., McMillan, E.L., Zhang, S.-C., and Gamm, D.M. (2009). Modeling early retinal development with human embryonic and induced pluripotent stem cells. Proc. Natl. Acad. Sci. USA *106*, 16698–16703.

Meyer, J.S., Howden, S.E., Wallace, K.A., Verhoeven, A.D., Wright, L.S., Capowski, E.E., Pinilla, I., Martin, J.M., Tian, S., Stewart, R., et al. (2011). Optic vesicle-like structures derived from human pluripotent stem cells facilitate a customized approach to retinal disease treatment. Stem Cells *29*, 1206–1218.

Nakano, T., Ando, S., Takata, N., Kawada, M., Muguruma, K., Sekiguchi, K., Saito, K., Yonemura, S., Eiraku, M., and Sasai, Y. (2012). Self-formation of optic cups and storable stratified neural retina from human ESCs. Cell Stem Cell *10*, 771–785.

O'Hara-Wright, M., and Gonzalez-Cordero, A. (2020). Retinal organoids: a window into human retinal development. Development *147*. dev189746.

Orozco, L.D., Chen, H.-H., Cox, C., Katschke, K.J., Arceo, R., Espiritu, C., Caplazi, P., Nghiem, S.S., Chen, Y.-J., Modrusan, Z., et al. (2020). Integration of eQTL and a single-cell atlas in the human eye identifies causal genes for age-related macular degeneration. Cell Rep. *30*, 1246–1259.e6.

Osakada, F., Ikeda, H., Mandai, M., Wataya, T., Watanabe, K., Yoshimura, N., Akaike, A., and Takahashi, M. (2008). Toward the generation of rod and cone photoreceptors from mouse, monkey and human embryonic stem cells. Nat. Biotechnol. *26*, 215–224.

Petrus-Reurer, S., Lederer, A.R., Baqué-Vidal, L., Douagi, I., Pannagel, B., Khven, I., Aronsson, M., Bartuma, H., Wagner, M., Wrona, A., et al. (2022). Molecular profiling of stem cell-derived retinal pigment epithelial cell differentiation established for clinical translation. Stem Cell Rep. *17*, 1458–1475.

Rao, A., Barkley, D., França, G.S., and Yanai, I. (2021). Exploring tissue architecture using spatial transcriptomics. Nature *596*, 211–220.

Reichman, S., Terray, A., Slembrouck, A., Nanteau, C., Orieux, G., Habeler, W., Nandrot, E.F., Sahel, J.-A., Monville, C., and Goureau, O. (2014). From confluent human iPS cells to self-forming neural retina and retinal pigmented epithelium. Proc. Natl. Acad. Sci. USA *111*, 8518–8523.

Ribeiro, J., Procyk, C.A., West, E.L., O'Hara-Wright, M., Martins, M.F., Khorasani, M.M., Hare, A., Basche, M., Fernando, M., Goh, D., et al. (2021). Restoration of visual function in advanced disease after transplantation of purified human pluripotent stem cell-derived cone photoreceptors. Cell Rep. *35*, 109022.

Sapkota, D., Chintala, H., Wu, F., Fliesler, S.J., Hu, Z., and Mu, X. (2014). Onecut1 and Onecut2 redundantly regulate early retinal cell fates during development. Proc. Natl. Acad. Sci. USA *111*, E4086–E4095.

Shiau, F., Ruzycki, P.A., and Clark, B.S. (2021). A single-cell guide to retinal development: cell fate decisions of multipotent retinal progenitors in scRNA-seq. Dev. Biol. *478*, 41–58.

Sinha, R., Lee, A., Rieke, F., and Haeseleer, F. (2016). Lack of CaBP1/caldendrin or CaBP2 leads to altered ganglion cell responses. eNeuro *3*. ENEURO.0099-16.2016.

Sridhar, A., Hoshino, A., Finkbeiner, C.R., Chitsazan, A., Dai, L., Haugan, A.K., Eschenbacher, K.M., Jackson, D.L., Trapnell, C., Bermingham-McDonogh, O., et al. (2020). Single-cell transcriptomic comparison of human fetal retina, hPSC-derived retinal organoids, and long-term retinal cultures. Cell Rep. *30*, 1644–1659.e4.

Thomas, E.D., Timms, A.E., Giles, S., Harkins-Perry, S., Lyu, P., Hoang, T., Qian, J., Jackson, V.E., Bahlo, M., Blackshaw, S., et al. (2022). Cell-specific cis-regulatory elements and mechanisms of non-coding genetic disease in human retina and retinal organoids. Dev. Cell *57*, 820–836.e6.

Voigt, A.P., Mullin, N.K., Whitmore, S.S., DeLuca, A.P., Burnight, E.R., Liu, X., Tucker, B.A., Scheetz, T.E., Stone, E.M., and Mullins, R.F. (2021). Human photoreceptor cells from different macular

subregions have distinct transcriptional profiles. Hum. Mol. Genet. *30*, 1543–1558.

Völkner, M., Zschätzsch, M., Rostovskaya, M., Overall, R.W., Busskamp, V., Anastassiadis, K., and Karl, M.O. (2016). Retinal organoids from pluripotent stem cells efficiently recapitulate retinogenesis. Stem Cell Rep. *6*, 525–538.

Wahle, P., Brancati, G., Harmel, C., He, Z., Gut, G., Santos, A., Yu, Q., Noser, P., Fleck, J.S., Gjeta, B., et al. (2022). Multimodal spatiotemporal phenotyping of human organoid development. Preprint at bioRxiv. https://doi.org/10.1101/2022.03.16.484396.

Wahlin, K.J., Maruotti, J.A., Sripathi, S.R., Ball, J., Angueyra, J.M., Kim, C., Grebe, R., Li, W., Jones, B.W., and Zack, D.J. (2017). Photoreceptor outer segment-like structures in long-term 3D retinas from human pluripotent stem cells. Sci. Rep. *7*, 766.

West, E.L., Majumder, P., Naeem, A., Fernando, M., O'Hara-Wright, M., Lanning, E., Kloc, M., Ribeiro, J., Ovando-Roche, P., Shum, I.O., et al. (2022). Antioxidant and lipid supplementation improve the development of photoreceptor outer segments in pluripotent stem cell-derived retinal organoids. Stem Cell Rep. *17*, 775–788.

Yan, W., Peng, Y.-R., van Zyl, T., Regev, A., Shekhar, K., Juric, D., and Sanes, J.R. (2020). Cell atlas of the human fovea and peripheral retina. Sci. Rep. *10*, 9802.

Yang, P., Huang, H., and Liu, C. (2021). Feature selection revisited in the single-cell era. Genome Biol. *22*, 321.

Zhang, X., Wang, W., and Jin, Z.-B. (2021). Retinal organoids as models for development and diseases. Cell Regen. *10*, 33.

Zhong, X., Gutierrez, C., Xue, T., Hampton, C., Vergara, M.N., Cao, L.-H., Peters, A., Park, T.-S., Zambidis, E.T., Meyer, J.S., et al. (2014). Generation of three dimensional retinal tissue with functional photoreceptors from human iPSCs. Nat. Commun. *5*, 4047.

Zhu, C.C., Dyer, M.A., Uchikawa, M., Kondoh, H., Lagutin, O.V., and Oliver, G. (2002). Six3-mediated auto repression and eye development requires its interaction with members of the Groucho-related family of co-repressors. Development *129*, 2835–2849.

Stem Cell Reports, Volume *18*

Supplemental Information

Comprehensive characterization of fetal and mature retinal cell identity to assess the fidelity of retinal organoids

Hani Jieun Kim, Michelle O'Hara-Wright, Daniel Kim, To Ha Loi, Benjamin Y. Lim, Robyn V. Jamieson, Anai Gonzalez-Cordero, and Pengyi Yang

Supplemental figures and legends

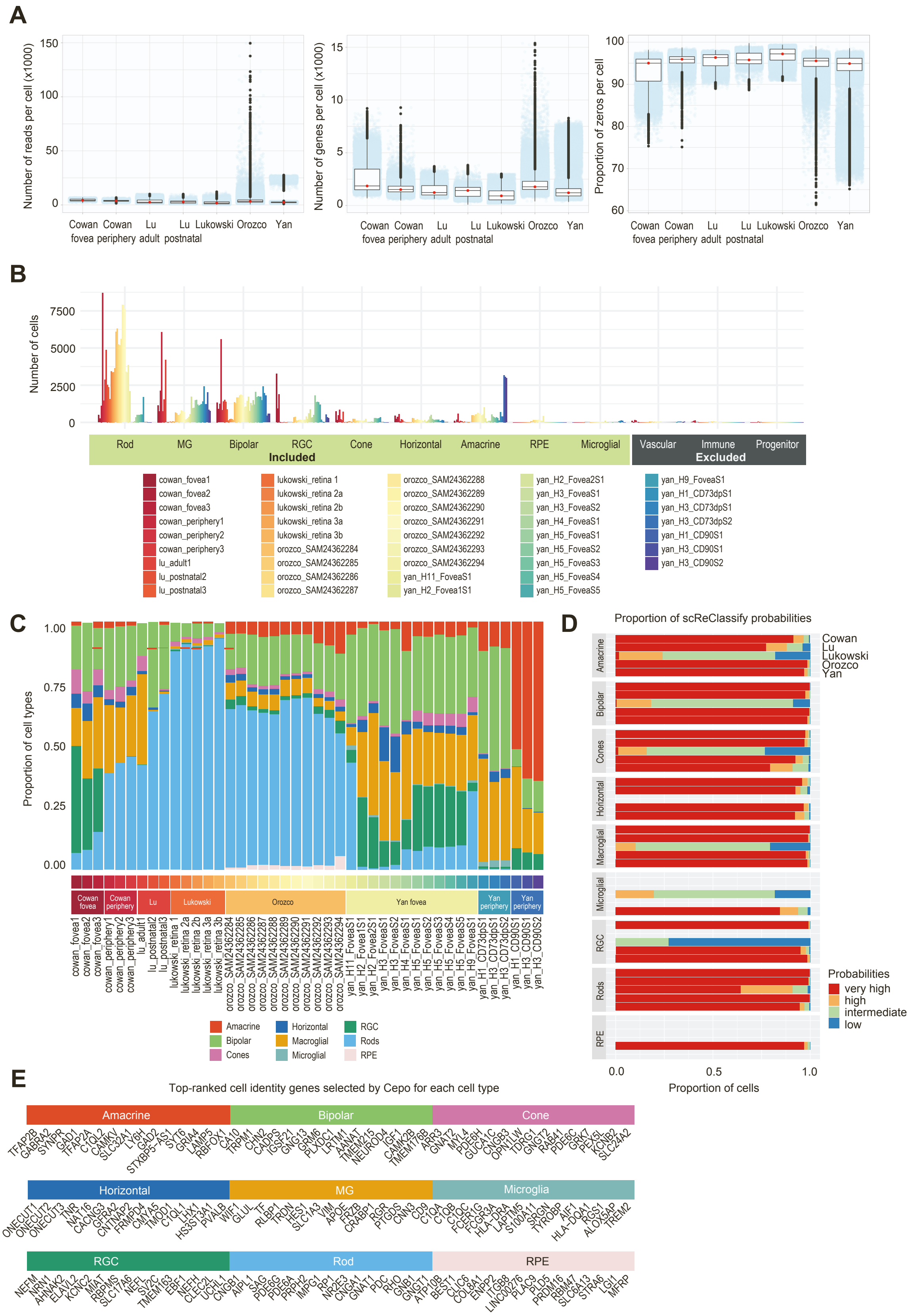

Figure S1. Quality control of retinal mature tissue scRNA-seq datasets. (A) Number of reads assigned, genes quantified, and proportion of zeros in each cell for each human retina dataset. **(B)**

Number of cells identified for each cell type in each dataset and batch. **(C)** Proportion of cell types (color coded) in each batch and dataset. Of note, samples from Yan et al. harvested from the periphery of the retina have been either depleted of rods (CD37dp) or depleted of rods and enriched for retinal ganglion cells (CD90). **(D)** Proportion of cells assigned with very high (>0.9), high (=< 0.9 and > 0.75), intermediate (<=0.75 and >0.5), or low (<= 0.5) probabilities according to scReClassify. scReClassify probabilities denote the confidence in cell type annotation where a higher probability denotes higher confidence. **(E)** Top ranked cell identity genes selected for each cell type by Cepo. Genes are ordered in the same order as the rows in the heatmap of **Figure1E**.

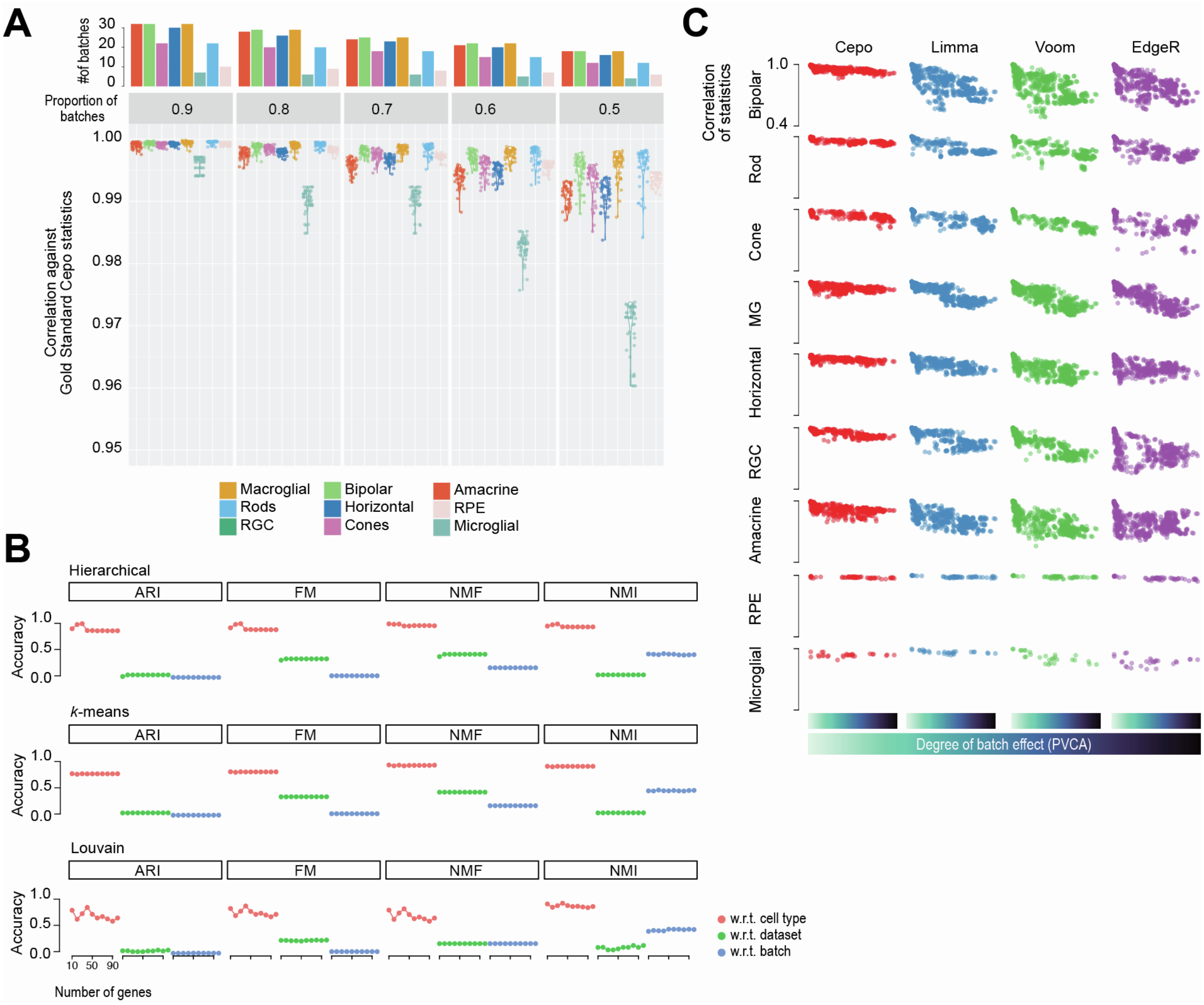

Figure S2. Assessment of batch effect on deriving cell-type-specific gene statistics. (A) Assessment of the stability of the gold standard Cepo statistics (i.e., the retinal cell identity map) with subsampling of the data at increasing rates (from 90 to 50% of the total number of batches). The Pearson's correlation coefficient of the correlation across overlapping genes (~15,000 genes) between the Cepo statistics from the full data and those derived from the subsampled data is plotted on the y-axis. For each subsampling study, the analyses were repeated 50 times using different seeds. **(B)** Clustering concordance quantified by adjusted rand index (ARI), Fowlkes Mallows index (FM), normalized mutual information (NMF), and normalized mutual information (NMI) with respect to cell type, dataset, and batch using hierarchical, Louvain, and k-means clustering algorithms. Number of genes used in clustering ranges from top 10 to 100 per cell type ranked by their Cepo statistics. **(C)** For each cell type, correlation of gene statistics calculated from Cepo, Limma, Voom, and EdgeR for each pair of datasets arranged by increasing batch effect as quantified by PVCA.

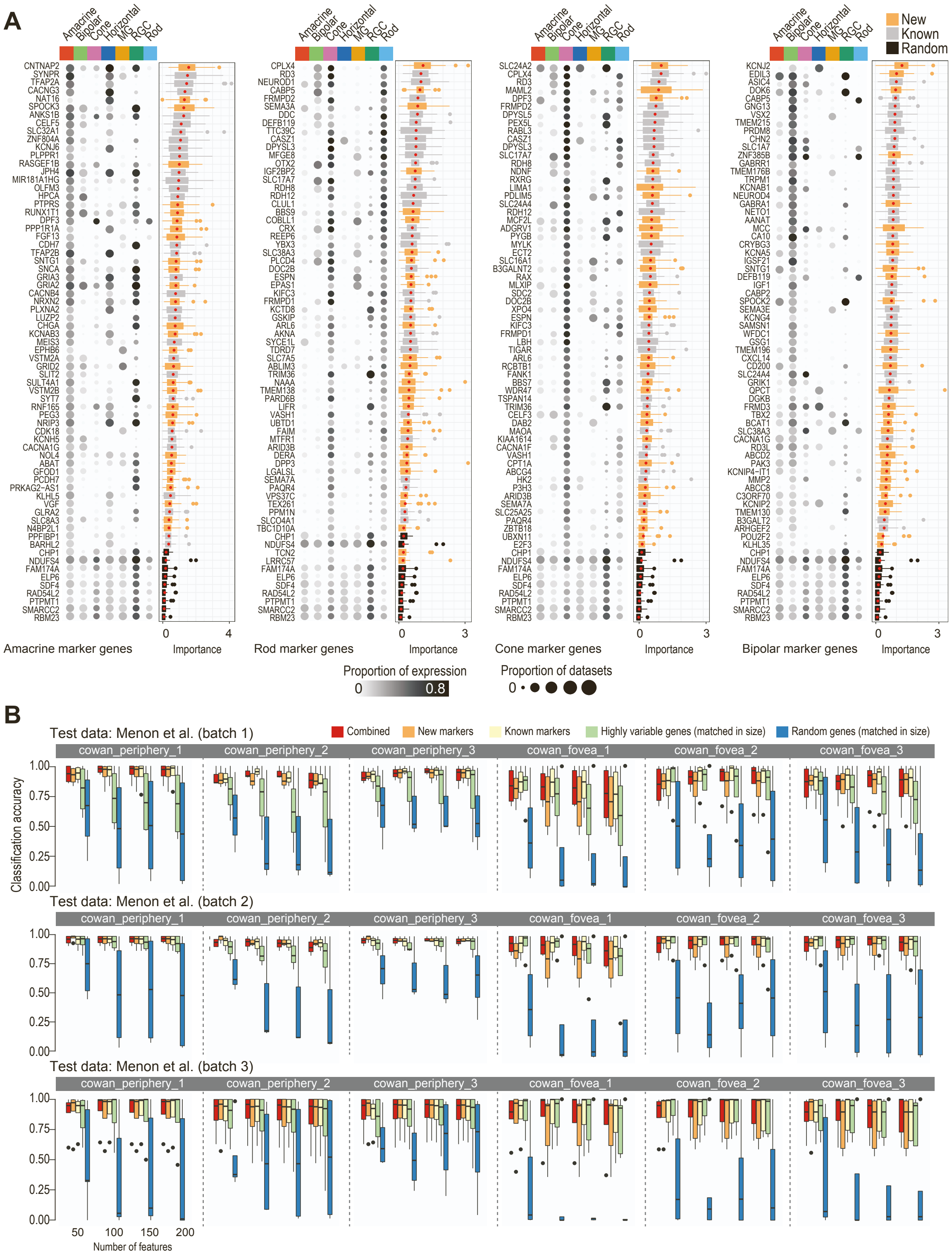

Figure S3. Cepo identification of cell-type-specific gene markers and their validation on external data. (A) Cell-type-specific gene markers identified by Cepo for Amacrine, Rods, Cone, and Bipolar cells. Proportion of cells expressing each marker in each cell type is represented by the gradient color and the proportion of datasets having each marker expressed is represented by the size of the balloons. Importance scores of gene markers are derived from random forest classification of cells using these markers. Novel markers are highlighted in orange and known markers are in gray.

Randomly selected genes (in black) are included as control. **(B)** Classification accuracy of each of the three batches of an independent test data (Menon et al.) from *k*NN classifiers trained on each of the five human retinal datasets and batches using various sets of gene markers (known, new, mixed, highly variable, and random).

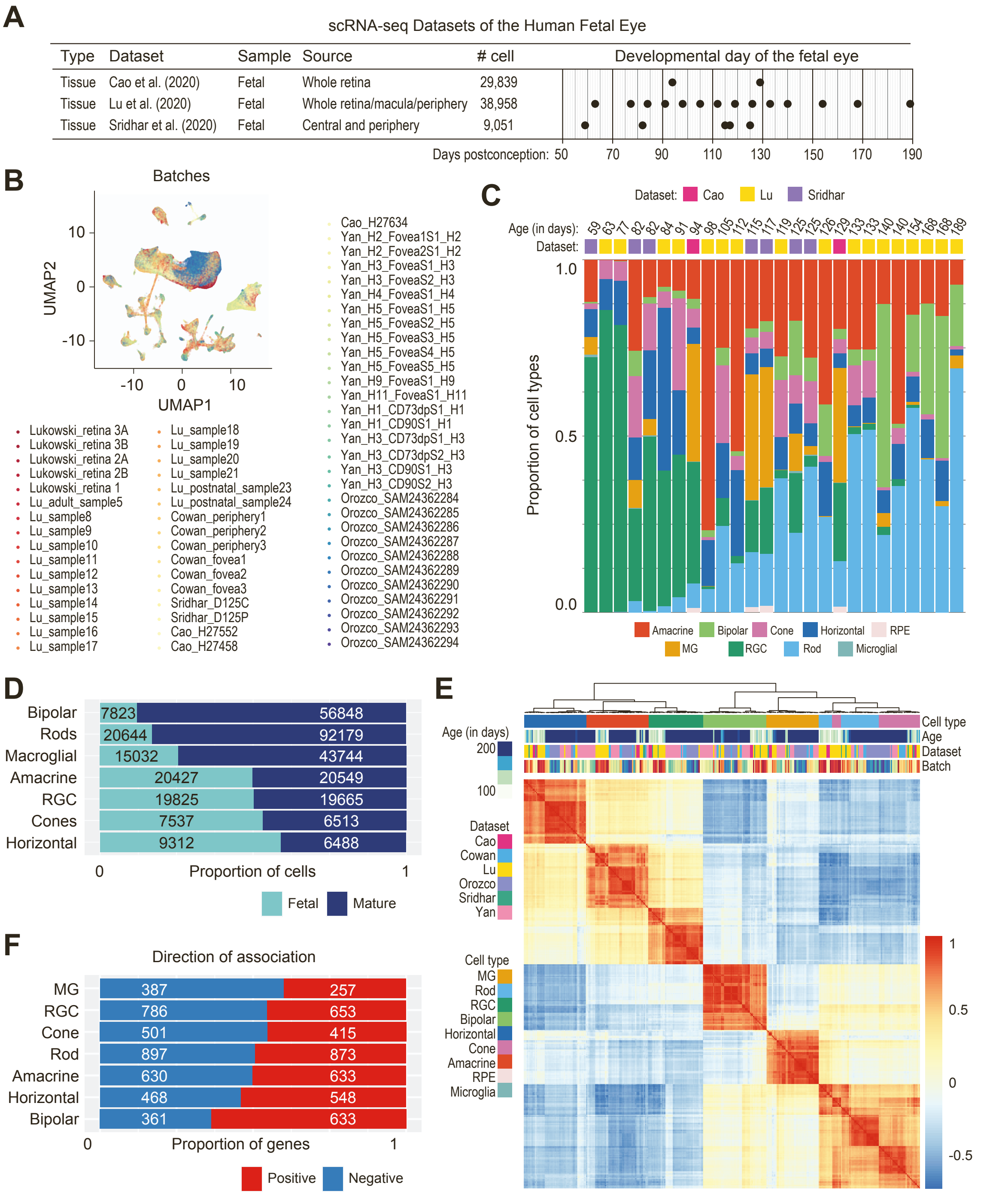

Figure S4. (A) Summary of scRNA-seq datasets collected from the retinal tissue. **(B)** UMAP representation of the transcriptomes of single cells from the mature and fetal atlas. Cells are coloured by their batch of origin. **(C)** Proportion of cell types (color coded) in each batch and dataset. **(D)** Proportion of mature and fetal cells (color coded) and their total number of cells in each cell type. **(E)** Correlation heatmap of cell-type-specific gene statistics generated from Cepo (Kim et al., 2021) for each cell type across datasets and batches. The heatmap is hierarchically clustered by the similarity of correlation profiles. **(F)** Proportional plot illustrating the direction of association among the significant genes (FDR-adjusted p-value < 0.05). The total number of genes in each set is noted in the plot.

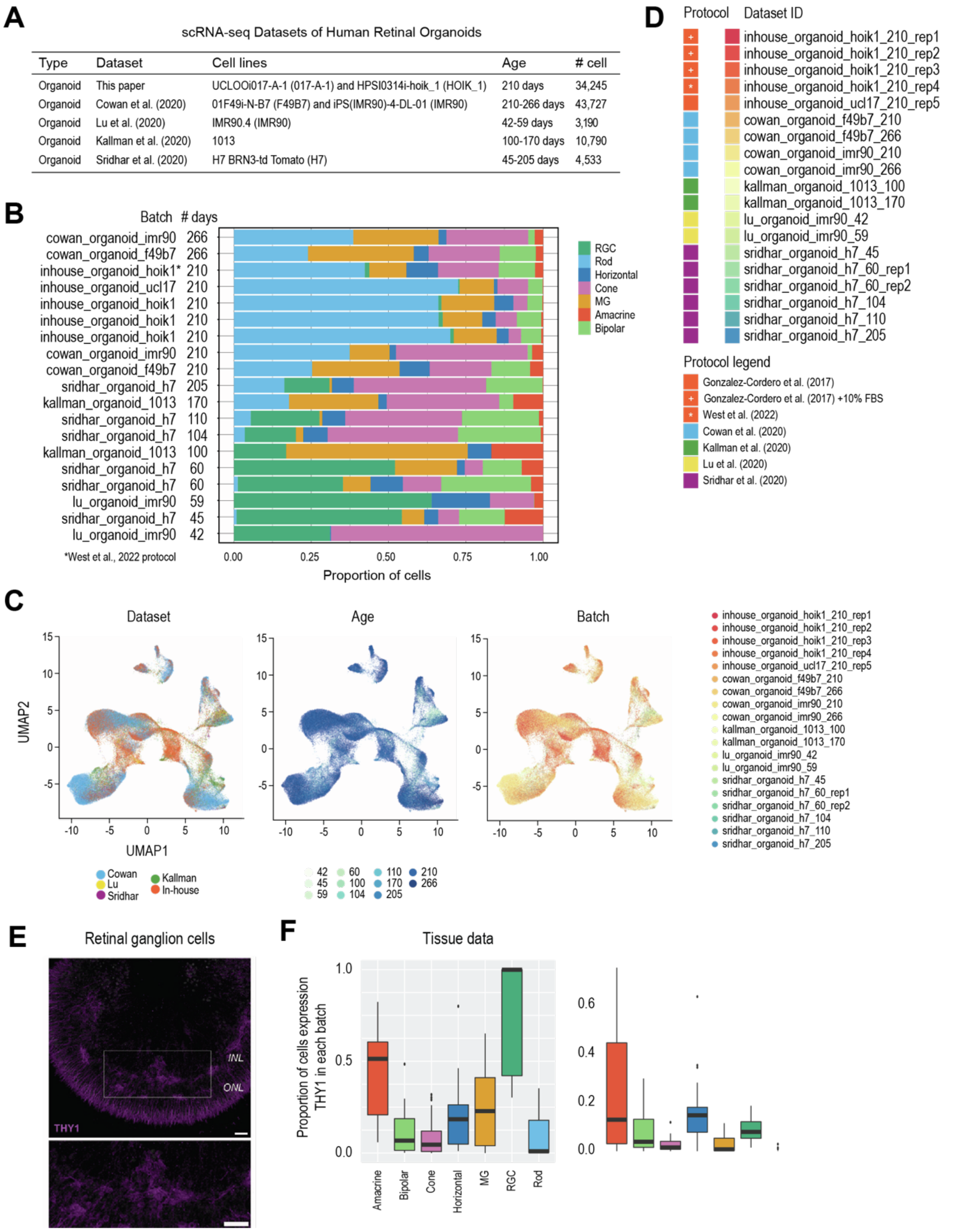

scRNA-seq Datasets of Human Retinal Organoids

Type	Dataset	Cell lines	Age	# cell
Organoid	This paper	UCLOOi017-A-1 (017-A-1) and HPSI0314i-hoik_1 (HOIK_1)	210 days	34,245
Organoid	Cowan et al. (2020)	01F49i-N-B7 (F49B7) and iPS(IMR90)-4-DL-01 (IMR90)	210-266 days	43,727
Organoid	Lu et al. (2020)	IMR90.4 (IMR90)	42-59 days	3,190
Organoid	Kallman et al. (2020)	1013	100-170 days	10,790
Organoid	Sridhar et al. (2020)	H7 BRN3-td Tomato (H7)	45-205 days	4,533

Figure S5. (A) Summary of scRNA-seq datasets collected from the retinal organoid studies. **(B)** Proportion of cell types (color-coded) and total number of cells in each batch and dataset. **(C)** UMAP representation of the transcriptomes of single cells from the organoid atlas. Cells are coloured by the dataset (left), the age until which the organoid was cultured (middle), and their origin of batch (right). **(D)** Legend showing the color annotations and dataset IDs of organoid scRNA-seq samples used in this study and their corresponding protocols. **(E)** Immunohistochemistry staining of Thy1, a marker of retinal ganglion cells, in a 210-day old organoid cultured using the West et al. 2022 protocol. **(F)** Boxplot showing the proportion of cells expressing Thy1 in each cell type (x-axis) and batch (individual data points) in the retina tissue samples (left panel) and organoids (right panel).

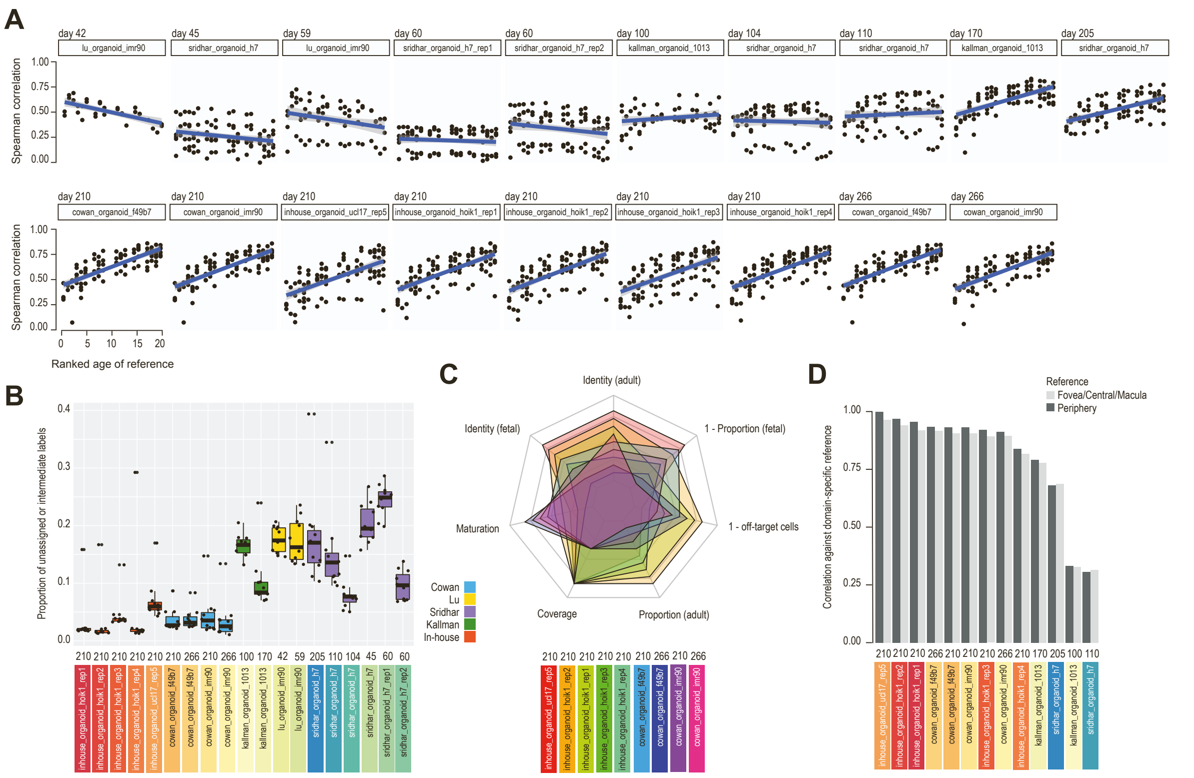

Figure S6. (**A**) Scatter plot of the correlation score of the cell identity scores of the organoid against each of the original reference scores from the tissue data. The y-axis denotes the Spearman correlation coefficient and the x-axis denotes the ranked developmental age of the original reference. The results from each organoid sample have been plotted separately. The scatter plot has been ordered from the youngest to the oldest organoid. (**B**) Boxplot showing the proportion of cells that were unassigned or showed hybrid cell-type annotations, indicative of off-target cells, in each organoid sample. Each datapoint denotes the replicates (n = 11) for each of the training data used in the scClassify framework. (**C**) Radar chart showing the order of ranks of organoids of each metric. (**D**) Barplot showing the correlation between the identities of the organoids (x-axis) and the domain-specific reference (Periphery or Fovea/Central/Macula). The average Spearman's correlation coefficient across the cell types is plotted on the y-axis. The barplots have been ordered by the highest to lowest correlation against the reference (from left to right).

Supplemental experimental procedures

QUANTIFICATION AND STATISTICAL ANALYSIS

Public single-cell RNA-seq datasets

Data collection

The raw gene-cell count matrices of scRNA-seq datasets were retrieved from the NCBI Gene Expression Omnibus (GEO) unless otherwise stated. The datasets are all related to the human retina, originating either from tissue or organoid and from mature or immature cells. Of note, we included only the datasets containing at least 10,000 cells to ensure enough cells in each batch and sample for the cell identity analysis.

Processing of scRNA-seq and filtering of cell types and suboptimal samples

All scRNA-seq libraries from human retinal tissue and organoids were integrated by Seurat (v4.0) (Hao et al., 2021) under the R (v4.1.1) environment. For each of the published datasets, the cell type labels from the original study were used. For datasets with unresolved cell type labels, such as "photoreceptors" and "AC/HC", we performed re-annotation of these subset of cells using scClassify (Lin et al., 2020) trained on the respective sample type (tissue or organoid) from the Lu et al. dataset (Lu et al., 2020), resolving them into either the Rods or Cones and Amacrine or Horizontal cells.

To establish a high-quality cell identity reference, we devised a framework to filter for suboptimal cells. Specifically, we performed a uniform cell type re-annotation procedure across all the tissue data using scReClassify (Kim et al., 2019), which is a semi-supervised learning method for assessing cell type annotation quality in the original classification from the scRNA-seq datasets. Only cells that received probabilities of correct assignment of greater than 0.9 were included in the study. Any datasets that resulted in greater than 50% of cells being mis-labeled were not included in the generation of the reference atlas.

Derivation of cell identity gene statistics and assessment of their concordance across batch

Computation of cell identity gene statistics

To derive the cell identity gene statistics for the human retina atlas, the count matrix of cell-gene variables were first log-transformed and normalized using the logNormCounts function from the scater package (McCarthy et al., 2017). The transformed and normalized data from each batch were subsequently analyzed using the Cepo package (Kim et al., 2021) for quantifying cell identity gene statistics for each major cell type based on the differential stability metric. For comparison, alternative methods (e.g., limma (Ritchie et al., 2015), voom (Law et al., 2014), edgeR (Robinson et al., 2010)) based on differential expression analysis were also used for calculating cell-type-specific gene statistics.

Evaluation of concordance of cell identity gene statistics after subsampling of the data

To evaluate the robustness of the cell identity statistics against subsampling of the data, we assessed the loss in Pearson's correlation between the cell identity statistics derived from the subsampled and those derived from the full data. The data was downsampled by randomly selecting 90% to 50% of the total number of batches. The analysis was repeated 50 times each with a different seed for each cell type.

Evaluation of concordance of cell identity gene statistics between batches

The evaluation of scRNA-seq datasets from multiple sources oftentimes requires technical noise to be removed. This so-called "batch effect" has been widely addressed through the development of several algorithms (Tran et al., 2020). However, the danger of overcorrection and restriction to a subset of features during the correction has hampered the widespread use of batch correction methods for downstream analysis. Therefore, an approach that bypasses the need to remove batch effects, does not compromise the biological signal, and retains many of the features is ideal to enable a comprehensive comparison between multiple atlases, often with a high degree of sparsity.

To this end, we assessed the fidelity of cell identity gene statistics between varying degrees of batch effect to determine the comparability of Cepo statistics across independent batches. A combinatorial assessment of cellular identity gene statistics concordance was performed between pairs of datasets. For all combinations of samples in the adult retinal tissue atlas, the extent of variance in principal component space contributed by batch and was quantified using the pvca package in R (Bushel,

2021). Briefly, principal variance component analysis fits a mixed linear model using the factor of interest, batch source, as an independent random effect to the selected principal components. Using the eigenvalues as weights, the associated variations of factors are standardized and the magnitude of the source of variability is presented as a proportion of the total variance. Therefore, a greater proportion of total variance denotes the presence of greater batch effect between the dataset pair.

To evaluate the concordance in cell identity gene statistics between the batches, the Cepo statistics were computed for each gene and for each cell type independently within each batch. Then for each batch pair, the concordance of cell identity was measured as the Pearson's coefficient of the correlation between the Cepo statistics. Finally, the relationship between the concordance and the degree of batch effect was examined. For comparison, cell-type-specific gene statistics from Limma, Voom, and edgeR were also analyzed using the same procedure.

Clustering of cell identity gene statistics and evaluation of batch mixing

To evaluate the consistency of the cell identity gene statistics derived for each cell type, clustering was performed on these statistics derived from different batches and datasets and then the degree of batch mixing was determined for three sources of variation: cell type, dataset source, and batch source. Ideally, cell identity gene statistics should reflect the biological identity of the cells and therefore the source of variation should solely arise from cell type identity and not from batch or dataset source.

To generate the cluster, three clustering algorithms (hierarchical clustering, k-means clustering, and Louvain clustering were performed. For each of the sources of variation, the resolution of the hierarchical and k-means clustering was set by controlling the parameter, number of trees or $centers$, respectively, to equal the number of cell types (7), the number of datasets (3), or the number of batches (14) present in the comparison. For the Louvain clustering wherein setting the exact cluster number is infeasible, the resolution was controlled by setting differing values of k when building the shared nearest neighbor graph (cell type, k = 5; dataset, k = 8; and batch, k = 2).

To assess the clustering performance of Cepo-derived cell identity gene statistics, we used the adjusted rand index (ARI) or the normalized mutual information (NMI) to evaluate the concordance of clustering results with respect to the cell type labels, batch, and dataset source, denoted respectively as either $ARI_{cell\ Types}$, $ARI_{dataset}$, and ARI_{batch} or $NMI_{cell\ Types}$, $NMI_{dataset}$, and NMI_{batch}. Considering that the Cepo-derived gene statistics are partitioned into different classes with respect to cell type labels, batch, or dataset source, let a be the number of pairs of samples partitioned into the same class by a clustering method, b be the number of pairs of samples partitioned into the same cluster but in fact belong to different classes, c be the number of pairs of samples partitioned into different clusters but belong to the same class, and d be the number of pairs of samples from different classes partitioned into different clusters. Then the ARI is calculated as follows:

$$ARI = 2(ad - bc)/(a + b)(b + d) + (a + c)(c + d)$$

Considering that the Cepo statistics are partitioned into different classes with respect to cell type labels, batch, or dataset source, let Y be the clustering outcome by a clustering method and C be the original labels of the different classes. Given that Y and C are the partitions of the same data, the overlaps between the two random variables can be counted and represented as a contingency table. Using information theory to measure agreement between the partitions and maximum likelihood estimation, the empirical joint distributions of clusterings Y and C are measured. Therefore, using the probabilities that an element falls into a given cluster, the entropy for clusterings Y and C, $H(Y)$ and $H(C)$, respectively, and the mutual information $I(Y;C)$ can be calculated. Then the NMI is calculated as follows:

$$NMI(Y, C) = 2 * I(Y;C)/H(Y) + H(C)$$

Identification of novel markers of cell type

Categorization of known and unknown cell-type markers

To systematically categorize a predicted marker gene as either already described in the literature or a new cell-type marker, an advanced PubMed query was performed using the R package easyPudMed (Fantini, 2019) for each of the top 500 marker genes. For each cell type, the advanced query consisted of three search terms: 1) the name of the candidate gene of interest; 2) the cell type of

interest; 3) and the term “RETINA”, all combined with the operator “AND”. The search terms covered “All Fields” of the possible search items. Any genes for which the PubMed search did not return a publication were categorized as a novel marker gene for the cell type of interest. In contrast, any genes for which the PubMed search did return at least one publication were considered known marker genes for the cell type of interest. One of the limitations of the PubMed query is that the individual publications returned as associated with a gene and cell type combination were not individually assessed for false positive result. Nevertheless, this approach provides a systematic and fast means to screen for novel markers, overcoming the need to manually survey the literature for all combinations of genes and cell types.

Quantification of feature importance

To demonstrate the importance of the potential marker genes, we performed feature selection analysis based on the random forest classifier (Breiman, 2001). The Gini index, which measures how important a selected feature is when training the random forest classifier, was used as a proxy for feature importance. To build the random forest classifier, the single-cell transcriptomes were subjected to random stratified sampling to 30% of its original size, and then pseudo-bulk transcriptomes for each cell type were generated by taking the mean expression of the genes. Approximately 350 pseudo-bulk transcriptomes were generated by repeating the sampling procedure 50 times. The random forest classifier was trained using these transcriptomes. As control, 10 cell-type invariant genes, as determined by their Cepo statistics, were included. A separate random forest classifier was built for each batch. Finally, the values for the mean decrease in Gini were extracted from the classifiers and visualized as a boxplot.

Classification of test data using known and novel gene markers

To further support the utility of the identified genes as novel markers of their respective cell types, we performed classification of single-cell transcriptomes of the human retina from an independent study (Menon et al., 2019), which was not included in the datasets used to derive the cell identity gene statistics. The *k*-nearest neighbor (*k*NN) classifiers were trained by varying the following four conditions:

Training data. Single-cell transcriptomes from six batches of data from the Cowan et al. (2021) study were used. The three batches of single-cell transcriptomes were each derived from either the periphery or the fovea.

Testing data. Single-cell transcriptomes from three batches of data from the Menon et al. (2019) study were used. The scRNA-seq data were derived from human retina tissue from either the macula in the central retina or a region of the mid-peripheral retina. Of note, the retina was mechanically separated from the retinal pigment epithelium-choroid.

Number of gene markers. The number of gene markers included in the training.

Category of gene markers. The top gene markers were derived from five categories: (1) gene markers categorized as known; (2) gene markers categorized as novel; (3) a gene set with both known and new markers; (4) highly variable genes; and (5) randomly selected genes. For gene sets 1-3, the top genes were ordered by Cepo statistics. For gene set 4, the top genes were ordered by the FDR-adjusted p-values computed from fitting a trend on the variance and mean of the log gene expression values.

Finally, the number of neighbors was set to *k* = 3 for all the *k*NN classifiers, and the final classification accuracy calculated for each cell type as follows:

$$Accuracy_{celltype} = Number\ of\ correct\ prediction_{celltype}\ /\ Total\ number\ of\ predictions_{celltype}$$

Identification of maturation-associated genes in retinal development

Determination of genes associated with maturation

To determine the genes associated with maturation, the cell-type-specific Cepo statistics derived for all the batches originating from the fetal and mature retinal atlas were used. Specifically, for each gene and each cell type, we computed the Spearman correlation coefficient as a function of the change in its Cepo statistics in that cell type over developmental age.

Measurement of similarity of maturation-association profiles between cell types

To investigate the similarity in the profile of maturation between cell types, we performed hierarchical clustering on the pairwise correlation matrix of the Spearman coefficient statistics on all the genes

found to be significantly associated with maturation (FDR-adjusted p-value < 0.05) in at least one cell type. The similarity is visualized as a clustered heatmap where 1 denotes complete positive correlation and -1 denotes complete negative correlation.

Gene set over-representation analysis of maturation-associated genes

Gene set over-representation analysis was performed on the genes significantly associated with maturation. Significance in association was determined as the FDR-adjusted p-value lower than 0.05. The gene set enrichment analysis was performed for each cell type and on either the gene sets positively or negatively associated with maturation. Using the GO terms related to biological processes from the C5 ontology gene set from the MSigDB collection (Liberzon et al., 2011), we assessed the over-representation of these gene sets among the maturation-associated genes using the fgsea R package (Korotkevich et al., 2022).

Analysis and benchmarking of retinal organoid protocols

Similarity of organoid protocols to one another

To evaluate the closeness of the organoid protocols to one another, we first performed Cepo analysis on the individual batches of the organoid datasets as in subsection "Computation of cell identity gene statistics" to derive cell identity gene statistics for each of the major cell types in each batch. Then, intersecting on the genes that are commonly found in all 19 batches, we aggregated the Cepo-derived gene statistics into a single vector. The similarity of cell identity profiles between the batches was evaluated in terms of the Pearson's correlation coefficient between the aggregated statistics.

Evaluation of the fidelity of organoids to the human tissue

To evaluate the fidelity of organoids to the human tissue, we generated the following six evaluation metrics:

(1) **The cell-type-specific similarity against the cell identity retina reference**

The cell-type-specific similarity of the query organoid against the cell identity retina reference resolved by mature and developmental cells were computed. The average Cepo statistics from the mature and the developmental cell types were taken as the reference. Then, to

compute the cell-type-specific similarity of the query data, we calculated the Pearson's correlation coefficient between the Cepo statistics derived for the query data and each of the references for each cell type.

(2) The overall similarity against the cell identity retina reference

The averaged score of the cell-type-specific similarities from (1) were computed for each protocol across cell types to generate the overall cell identity score. As in (1), a higher score denotes stronger fidelity to the retinal reference and a lower score denotes weaker fidelity.

(3) The coverage of cell types

The coverage of cell types generated from the organoid protocol was calculated. The expected retinal cell types are cones, rods, Müller glial, ganglion, bipolar, horizontal, and amacrine cells. Protocols with the capacity to generate all cell types were assigned a score of 1, whereas those with nil capacity to generate the expected cell types were assigned a score of 0.

(4) Maturation profile

The developmental phase relevancy of the organoid was calculated by measuring the capacity of organoids to recapitulate the developmental change across time. Specifically, by computing the Spearman's correlation between the concordance between the cell identity scores of the organoid protocols and all the individual cell identity scores from the tissue adult and fetal data ordered by time, the final score reflects whether the organoids are more adult-like or fetal-like. This was performed for each cell type and final score was generated by averaging the results. A protocol with a high positive score denotes a more adult-like profile, whereas those with a high negative score denotes a more fetal-like profile.

(5) The concordance in cell-type proportion with the retina reference

The concordance against the proportion of cell types expected in the retina was measured using the averaged proportion profiles of the mature and fetal retina tissue datasets. To account for the differences in cell-type proportions that result from early and late-born cell types in early and late retinogenesis, the fetal proportional reference was sub-categorized into two time points (early [<97 days] and late [>97 days]). The intraclass correlation coefficient (ICC) for oneway models was used to compute an index of consistency of the proportions (Gamer et al.). A protocol with a high ICC reflects a high capacity to reciprocate the

proportions found in the real tissue, whereas those with a low ICC reflect those with a low capacity.

(6) **Proportion of off-target cells**

The proportion of potential off-target cells in the data was measured using scClassify (Lin et al., 2020). We first constructed a cell type hierarchical tree using HOPACH using the Orozco et al. samples as our reference dataset and used the weighted KNN classifier to assign each cell to a cell type. This procedure is repeated for the 11 individual batches in the reference data. The Limma package was used to select the top 50 features. A key feature of scClassify is that 1) it does not force a cell to be assigned to a cell type and 2) it allows cells to be annotated as an intermediate cell type (i.e., labelled as a hybrid cell from the non-terminal node of the hierarchy). Thus, any cells assigned by scClassify as an “unassigned” or “intermediate” cell type were considered potential off-target cells. A protocol with a low proportion of off-target cells reflects a high capacity to generate on-target cells, whereas those with a high proportion of off-target cells reflect low capacity to generate the in vivo cell types of the retina.

For each metric, except the proportion of off-targets, we then aggregated the results for all the benchmarked protocols and rescaled the score to a range of [0, 1]. Finally, equally weighting these metrics, the average of the overall cell identity (mature and fetal), the coverage, the cell-type proportion (mature), maturation, 1 minus proportion of off-targets, and 1 minus the cell-type proportion (fetal, early) was taken to generate a final score. This score was used to benchmark the retinal organoid protocols in terms of their fidelity to the human retinal tissue. Finally, whilst not included in the final metric, we generated the cell identity reference for each cell type for the central (including the fovea and macula) and periphery adult retina generated from the Cowan et al. and Yan et al. samples.

Single-cell RNA sequencing of retinal organoids and human retina

Dissociation of organoids into single cells

Five independent organoid batches were derived for both HPSI0314i-hoik_1 and UCLOOi017-A-1 hiPSC lines. One organoid was dissociated per sample. Retinal organoids were dissociated into

single cell suspension using the Neurosphere Dissociation Kit (P) (Miltenyi Biotec). Enzymatic digestion was performed as per manufacturer protocol for 10 minutes at 37°C with intermittent agitation, followed by gentle mechanical dissociation with a p1000 pipette, and a further 5 minute 37°C incubation. The cell suspension was passed through a p200 to ensure single cell dissociation before the enzymatic reaction was stopped by washing with HBSS. The cell suspension was filtered through MACS SmartStrainer 30µm (Miltenyi Biotec) before being pelleted by centrifugation at 400*g* for 10 minutes at room temperature. The cell pellet was resuspended in ALT90 and maintained on ice.

Single cell RNA-sequencing

Each single cell suspension of dissociated retinal organoid was assessed for viability using 0.4% Trypan Blue staining on a Countess II Automated Cell Counter (Invitrogen) and concentration was adjusted to 1000 cells/µl. Cell suspension was loaded on a single-cell-B Chip (10X Genomics) for a target output of 10,000 cells per sample. Single-cell droplet capture was performed on the Chromium Controller (10X Genomics). cDNA library preparation was performed in accordance with the Single-Cell 3' v3 protocol. Libraries were evaluated for fragment size and concentration using Agilent HSD5000 ScreenTape System. Samples were sequenced on an Illumina NovaSeq6000 instrument according to manufacturer's instructions (Illumina). Sequencing was carried out using 2×150 paired-end (PE) configuration with a sequencing depth of 40,000 reads per cell. The sequences were processed by GENEWIZ, China.

Analysis of in-house single-cell RNA sequencing data generated from retinal organoids

Read alignment and expression count table generation

From the sequencing results of the 10x Chromium experiments, the unique molecular identifiers, cell barcodes, and the genomic reads were extracted using Cell Ranger with default parameters (v3.1, 10x Genomics). The extracted reads were aligned against the annotated human genome, including the protein and non-coding transcripts (GRCh38, GENCODE v27). The reads with the same cell barcode and unique molecular identifier were collapsed to a unique transcript, generating the count matrix where columns correspond to single cells and rows correspond to transcripts. To remove potentially empty droplets with ambient RNA, the emptyDrops function from the DropletUtils package

was used. Droplets with significantly non-ambient profiles were called at a false discovery rate of 1%, applying the Benjamini-Hochberg correction to the Monte Carlo p-values to correct for multiple testing. Next, to remove potentially unhealthy or suboptimal cells, cell filtering was performed using the number of reads, the proportion of genes expressed, and the fraction of mitochondrial reads as criteria. Specifically, as cells with greater than 10,000 reads, 99% of genes not expressed, and 25% of mitochondrial gene expression were removed. Transcripts from mitochondrial- and ribosomal-protein coding genes were discarded for downstream analyses such as embedding and clustering, because they are typically known to be highly expressed irrespective of biological identity.

Doublet detection and filtering

The presence of multiplets in single-cell data can arise from incomplete dissociation of single cells meaning that more than one cell can be encapsulated in GEMs. DoubletFinder, an algorithm to detect multiplets in single-cell data, was used to remove potential doublets or multiplets from each biological batch at a threshold of 5.0% (McGinnis et al., 2019).

Integration and clustering

Embedding transcriptomes into a shared latent space

To embed the single-cell transcriptomes into a shared latent space, for each batch the count matrix was first normalized to the total number of reads and then factored by a 10,000 scaling factor. Then the top 2,000 features, among the top 1,000 highly variable features determined through variance stabilizing transformation, were prioritized by their variance across all the scRNA-seq batches. Next, the cell pairwise anchor correspondences between different single-cell transcriptome batches were identified with 30-dimensional spaces from reciprocal principal component analysis (Hao et al., 2021). Using these anchors, the scRNA-seq datasets were integrated and transformed into a shared space. Gene expression values were scaled for each gene across all integrated cells and used for principal component analysis (PCA). For the integration of the organoid datasets, $k.filter$ and $k.weight$ were set to 160 and 90, respectively, to accommodate the integration of datasets with fewer than 200 cells.

To generate the embeddings containing the single-cell transcriptomes derived from the mature tissue, the single cells were embedded into two-dimensional UMAP space by using the first 15 principal

components (PCs). To generate the embeddings containing the single-cell transcriptomes derived from the mature and development tissue combined, the single cells were embedded into two-dimensional UMAP space by using the first 30 principal components (PCs). Finally, to generate the embeddings containing the single-cell transcriptomes derived from the retinal organoids, the single cells were embedded into two-dimensional UMAP space by using the first 15 principal components (PCs).

Clustering and classification of in-house datasets

To cluster the single cells from the organoid datasets, the shared nearest neighbor graph was constructed on the first 30 PCs of the shared embedding using the default arguments of the FindNeighbors function in the Seurat package. Then the Louvain clustering algorithm with resolution equal to 1.1 was used to cluster the single cells. Classification of the single cells from the in-house datasets was performed by assigning them to the cell type that according to the labels of the public datasets that most dominate the cluster assigned to the cell of interest.

Development of the Eikon software

We implemented Eikon, an interactive web tool to facilitate the assessment of the fidelity of retinal organoids to the *in vivo* retinal tissue. Eikon accepts a SingleCellExperiment object (Lun et al., 2022). Multiple parameters can be specified in Eikon, including the assay, age of samples, and whether normalization is required. Users can customize the visualization plots using the provided options and all key visualizations are downloadable. Specifically, Eikon outputs three key visualizations, including several correlation heatmaps, reduced dimension plots, and an interactive table of Cepo statistics for each retinal cell type contained in the query data. The correlation heatmaps display the correlation cell identity scores between the query and reference datasets, allowing users to assess the fidelity of their data in a visually intuitive manner. Importantly, the query data can be compared to all or specific developmental stages of the reference dataset. PCA, UMAP, and t-SNE plots are also displayed and can be coloured by variables contained in the query data such as the proportion of zeroes and UMIs. Additionally, plots can be coloured according to the expression levels of a particular gene of interest which can be found using an interactive table displaying the Cepo statistics for each gene of each retinal cell type in the query data.

REFERENCES

Breiman, L. (2001). Random Forests. Machine Learning *45*, 5–32.

Fantini, D. (2019). easyPubMed: Search and Retrieve Scientific Publication Records from PubMed.

Gamer, M., Lemon, J., Fellows, I., and Puspendra, S. irr: Various Coefficients of Interrater Reliability and Agreement version 0.84.1 from CRAN.

Hao, Y., Hao, S., Andersen-Nissen, E., Mauck, W.M., Zheng, S., Butler, A., Lee, M.J., Wilk, A.J., Darby, C., Zager, M., et al. (2021). Integrated analysis of multimodal single-cell data. Cell *184*, 3573-3587.e29.

Kim, T., Lo, K., Geddes, T.A., Kim, H.J., Yang, J.Y.H., and Yang, P. (2019). scReClassify: post hoc cell type classification of single-cell rNA-seq data. BMC Genomics *20*, 913.

Korotkevich, G., Sukhov, V., Budin, N., and Sergushichev, A. (2022). fgsea: Fast Gene Set Enrichment Analysis (Bioconductor version: Release (3.15)).

Law, C.W., Chen, Y., Shi, W., and Smyth, G.K. (2014). voom: precision weights unlock linear model analysis tools for RNA-seq read counts. Genome Biology *15*, R29.

Liberzon, A., Subramanian, A., Pinchback, R., Thorvaldsdottir, H., Tamayo, P., and Mesirov, J.P. (2011). Molecular signatures database (MSigDB) 3.0. Bioinformatics *27*, 1739–1740.

Lin, Y., Cao, Y., Kim, H.J., Salim, A., Speed, T.P., Lin, D.M., Yang, P., and Yang, J.Y.H. (2020). scClassify: sample size estimation and multiscale classification of cells using single and multiple reference. Mol Syst Biol *16*, e9389.

Lu, Y., Shiau, F., Yi, W., Lu, S., Wu, Q., Pearson, J.D., Kallman, A., Zhong, S., Hoang, T., Zuo, Z., et al. (2020). Single-Cell Analysis of Human Retina Identifies Evolutionarily Conserved and Species-Specific Mechanisms Controlling Development. Dev Cell *53*, 473-491.e9.

Lun, A., Risso, D., Korthauer, K., and Rue-Albrecht, K. (2022). SingleCellExperiment: S4 Classes for Single Cell Data (Bioconductor version: Release (3.15)).

McCarthy, D.J., Campbell, K.R., Lun, A.T.L., and Wills, Q.F. (2017). Scater: pre-processing, quality control, normalization and visualization of single-cell RNA-seq data in R. Bioinformatics *33*, 1179–1186.

Menon, M., Mohammadi, S., Davila-Velderrain, J., Goods, B.A., Cadwell, T.D., Xing, Y., Stemmer-Rachamimov, A., Shalek, A.K., Love, J.C., Kellis, M., et al. (2019). Single-cell transcriptomic atlas of the human retina identifies cell types associated with age-related macular degeneration. Nat Commun *10*, 4902.

Ritchie, M.E., Phipson, B., Wu, D., Hu, Y., Law, C.W., Shi, W., and Smyth, G.K. (2015). limma powers differential expression analyses for RNA-sequencing and microarray studies. Nucleic Acids Research *43*, e47.

Robinson, M.D., McCarthy, D.J., and Smyth, G.K. (2010). edgeR: a Bioconductor package for differential expression analysis of digital gene expression data. Bioinformatics *26*, 139–140.

Tran, H.T.N., Ang, K.S., Chevrier, M., Zhang, X., Lee, N.Y.S., Goh, M., and Chen, J. (2020). A benchmark of batch-effect correction methods for single-cell RNA sequencing data. Genome Biology *21*, 12.
